# Perspectives on the methods of a large systematic mapping of maternal health interventions

**DOI:** 10.1186/s12992-016-0191-7

**Published:** 2016-08-25

**Authors:** Matthew Chersich, Victor Becerril-Montekio, Francisco Becerra-Posada, Mari Dumbaugh, Josephine Kavanagh, Duane Blaauw, Siphiwe Thwala, Elinor Kern, Loveday Penn-Kekana, Emily Vargas, Langelihle Mlotshwa, Ashar Dhana, Priya Mannava, Anayda Portela, Mario Tristan, Helen Rees, Leon Bijlmakers

**Affiliations:** 1 Wits Reproductive Health and HIV Institute, Faculty of Health Sciences, University of Witwatersrand, Johannesburg, South Africa; 2 Centre for Health Policy and MRC Health Policy Research Group, Faculty of Health Sciences, University of Witwatersrand, Johannesburg, South Africa; 3 Centre for Health Systems Research, National Institute of Public Health (Instituto Nacional de Salud Pública), Cuernavaca, Mexico; 4 Pan American Health Organization, Washington D.C, USA; 5 Department of Epidemiology and Public Health, Society, Gender and Health Unit, Swiss Tropical and Public Health Institute, Basel, Switzerland; 6 University of Basel, Basel, Switzerland; 7 Department of Infectious Disease Epidemiology, London School of Hygiene and Tropical Medicine, London, United Kingdom; 8 Innovation in Public Health Department, National Institute of Health, Bogotá D.C, Colombia; 9 Centre for International Health, Burnet Institute, Melbourne, Victoria Australia; 10 Department of Maternal, Newborn, Child and Adolescent Health, World Health Organization, Geneva, Switzerland; 11 IHCAI Foundation, San Jose, Costa Rica; 12 London School of Hygiene & Tropical Medicine, London, United Kingdom; 13 Radboud University Medical Center, Radboud Institute for Health Sciences, Nijmegen, The Netherlands

**Keywords:** Scoping review, Research methodology, Mapping of research, Systematic mapping, Maternal health, Health systems

## Abstract

**Background:**

Mapping studies describe a broad body of literature, and differ from classical systematic reviews, which assess more narrowly-defined questions and evaluate the quality of the studies included in the review. While the steps involved in mapping studies have been described previously, a detailed qualitative account of the methodology could inform the design of future mapping studies.

**Objectives:**

Describe the perspectives of a large research team on the methods used and collaborative experiences in a study that mapped the literature published on maternal health interventions in low- and middle-income countries (2292 full text articles included, after screening 35,048 titles and abstracts in duplicate).

**Methods:**

Fifteen members of the mapping team, drawn from eight countries, provided their experiences and perspectives of the study in response to a list of questions and probes. The responses were collated and analysed thematically following a grounded theory approach.

**Results:**

The objectives of the mapping evolved over time, posing difficulties in ensuring a uniform understanding of the purpose of the mapping among the team members. Ambiguity of some study variables and modifications in data extraction codes were the main threats to the quality of data extraction. The desire for obtaining detailed information on a few topics needed to be weighed against the benefits of collecting more superficial data on a wider range of topics. Team members acquired skills in systematic review methodology and software, and a broad knowledge of maternal health literature. Participation in analysis and dissemination was lower than during the screening of articles for eligibility and data coding. Though all respondents believed the workload involved was high, study outputs were viewed as novel and important contributions to evidence. Overall, most believed there was a favourable balance between the amount of work done and the project’s outputs.

**Conclusions:**

A large mapping of literature is feasible with a committed team aiming to build their research capacity, and with a limited, simplified set of data extraction codes. In the team’s view, the balance between the time spent on the review, and the outputs and skills acquired was favourable. Assessments of the value of a mapping need, however, to take into account the limitations inherent in such exercises, especially the exclusion of grey literature and of assessments of the quality of the studies identified.

**Electronic supplementary material:**

The online version of this article (doi:10.1186/s12992-016-0191-7) contains supplementary material, which is available to authorized users.

## Background

A systematic mapping of a body of literature explicitly sets out to examine the studies done on a topic or research area, as a means of describing a broad research field [1]. Naturally, the focus and extent of a mapping of literature varies with its aims, but can include the syntheses of research findings on particular topics, or of research methodologies, study settings, or even of characteristics such as authorship and funding of specific research fields [2]. Mapping studies use data extracted from full text publications or bibliometric methodology [3], to systematically identify and summarise a body of literature. The methodology shares some features with ‘scoping reviews’, or rapid non-systematic syntheses of literature [4, 5]. These are commonly done as part of the initial steps in the planning of a systematic review, or even to help make a decision about whether or not to undertake a systematic review. Text mining technologies are a relatively new alternative to classic screening methods and will expedite mapping and scoping reviews in future [6].

Much of the methods used in a systematic mapping of literature are consistent with the first steps in a systematic review [7, 8], such as searching databases to locate a body of literature, screening articles for eligibility and extracting data from full text articles. A mapping, however, unlike systematic reviews, often does not assess the quality of the included studies, or extract data on the outcomes of interventions that are studied. A mapping of a body of literature can, however, serve to identify articles on several specific topics, which may then be followed by a series of reviews on these topics. The maps can thus inform subsequent systematic reviews on one or more narrowly defined research questions [9]. Finally, mapping can be used to address research questions that are difficult to answer through classic systematic reviews. For example, using classic review methods, it would be difficult to devise a rigorous search strategy for identifying articles on health systems interventions in maternal health, or studies that report effects of interventions on specific sub-groups, while these are possible in a mapping.

The mapping study which is the topic of this paper aimed to synthesize research published on maternal health in low- and middle-income countries (LMICs) from 2000 to 2012, with a specific focus on interventions related to health inequities and systems. The year 2000 was selected because that was the onset of the Millennium Development Goals period, while the end date reflected the time that the review began. Studies had to include health systems, health promotion or community-based interventions; or interventions on one of five clinical tracer conditions: haemorrhage, hypertension, HIV, sexually transmitted infections other than HIV, or malaria. A sensitive search strategy combining controlled vocabulary and free-text terms was developed, following several exploratory searches and piloting. In the final search, terms for maternal health were combined with terms for LMICs, using the ‘AND’ function of search engines. The study also aimed to build capacity and reinforce collaboration across several research institutions and global regions.

This paper provides a qualitative critique of the mapping methods and outputs from the perspectives of the study team. As an overarching question, this article attempts to answer the question: ‘Did the number and quality of outputs match the time-intensive and repetitive nature of the work?’ Such information has not been provided previously in similar methodological assessments of mapping [4, 8, 9]. This paper discusses the perspectives of the review team about the methodology, and their overall experience of the project gathered through an unstructured, self-administered questionnaire. We also present the collaborative and learning experiences of the team, so as to assess the extent to which the study aims around collaboration and capacity building have been achieved.

## Methods

**Fig. 1:**
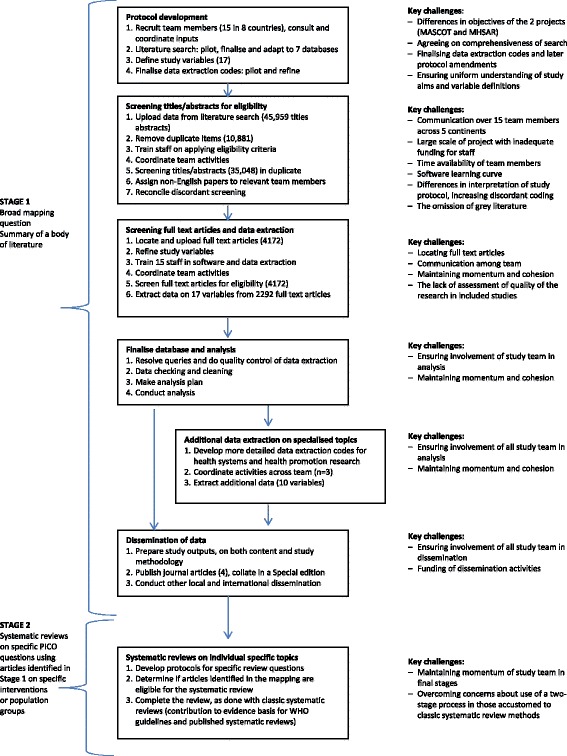
Review process and key challenges

Between October and November 2015, the study coordinator (MFC) emailed all members of the mapping team a list of open-ended questions and probes (Additional file 1). Those who had initially joined the team, but later discontinued participation, were also invited to offer their views. The data collection tool focused on the team’s perspectives on the strengths and weaknesses of various phases of the review, in particular the identification of relevant literature, especially non-English articles; data extraction from full text articles; and the data analysis and dissemination of the findings. Figure 1 shows the two-staged review process. The first stage involved screening of literature, data extraction, analysis and dissemination of a mapping that sums a body of literature on maternal health (Stage 1). This was followed by a number of systematic reviews on specific PICO questions (Population, Intervention Comparator Outcomes) for certain interventions or target populations, using the articles identified on these in the previous stage (Stage 2). The enquiry among team members focused on factors influencing the quality of the mapping procedures and how that could be improved. The questions and probes also encompassed a broader set of themes, specifically on whether the review’s objectives were clear and how these had changed over time; the perceived level of participation, collaboration and communication among the team; and the effectiveness of the capacity building activities of the study. Finally, views were elicited on the overall experience of the study; and whether it was considered worth the time and financial investments incurred.

At least two attempts were made to obtain responses from team members. Responses, emailed to the study coordinator, were not anonymised. Inputs were received from 15 of 18 researchers invited to participate. The responses were coded thematically, following a grounded-theory approach [10]. Key themes were identified and used to structure this report. The results text was reviewed by all team members for completeness and accuracy of interpretation. Illustrative quotes are included, together with a description of the respondents, where relevant.

The findings are grouped into three major themes, beginning with a critique of the different phases of the study and the factors in each phase that may have influenced the quality of the review. Thereafter, we assess the collaborative experiences of the team, as well as the success of the project in achieving its capacity building objectives. Finally, we consider the balance between the resources spent on the study and the project’s outputs.

## Factors influencing the quality of the review

### Identification of relevant literature

The search covered seven databases, including a regional database for South and Central America (Literatura Latino-Americana e do Caribe em Ciências da Saúde, Additional file 2). The search was considered as having adequately captured the literature, some team members even felt that the search criteria were not tight enough, resulting in too many articles to sort through. The volume was indeed large: 45,959 abstracts were uploaded, 10,881 duplicates removed, and 35,078 titles and abstracts screened independently by two reviewers. Differences between reviewers were resolved by a third, more senior reviewer. Details of reasons for excluding articles at each stage are provided elsewhere [2, 11].

A few team members felt that the search strategy could have included more specific “health system” terms like governance and accountability, so as to avoid missing articles on these topics. The exclusion of “grey literature” particularly concerned one team member with a health systems background, who noted that such literature documents the vast majority of implementation experiences and of high-quality research [12]. Of interest, a researcher who was involved in developing systematic reviews on individual review questions (Stage 2 articles using the MASCOT/MHSAR database), felt that others involved in those reviews ‘*may have felt uncomfortable with using a study method that was foreign’.* These concerns stemmed from fears that the mapping approach, which aims to identify articles on several different topics at one time, might not have located all relevant studies on the topics at hand, unlike with classic systematic reviews where separate searches are done for each individual review question.

Though several non-English databases were searched, and no language restrictions were employed, some felt that additional efforts could have been made to locate this literature. They were concerned that much of this research is published in journals which are not indexed by the major biomedical databases. An information specialist commented that: ‘*perhaps sources of information from other countries* [not found in the literature review] *could have been pursued more vigorously’* and a senior researcher from Costa Rica wondered if ‘*this work* [non-English literature] *was not fully used’.* Including publications in multiple languages and allocating them to native speakers was more complex than anticipated, with potential pitfalls at each stage of the review. Partners in non-English countries were especially important for locating full text articles. The South African who led the location and uploading of full text articles noted that: *‘tremendous efforts were made by participants all over the world to find the articles missing in each other’s libraries.’* Full text papers were located for 93 % of the abstracts classified as eligible for full text screening (4,175/4,472).

Finally, some external data sources were used and variables from these were merged into the database for particular analyses, allowing for comparison between our data and broader variables. These data included the journal’s Impact Factor [13]); Gross Domestic Product of each LMIC [14]; total number of health articles published per country [15]; and the number of maternal deaths [16] and of women with HIV infection in each LMIC [17].

### Extraction of data from full text articles

Defining the variables was technically the hardest part of the study, and it is fair to say that the coordinators underestimated how much discussion and time was required to finalise the coding system. The 17 study variables, operationalised as data extraction codes, covered the following: country where the study was done; country of affiliation of the first author; study design; intervention topic; whether the study examined health inequalities, health systems or health promotion; intervention recipient; period of pregnancy targeted; type of health or health systems outcomes reported; and research funder. Actual outcomes were not captured and, as in most similar mapping studies [1, 8], we did not assess the quality of the research methodology in individual studies. Some variables were harder to define than others. Even what constitutes an ‘intervention’ was debated vigorously; the boundary between provision of routine services and an intervention was not always easy to delineate. As a further example, the provision of medical supplies or commodities is one of the six WHO Health System Building Blocks [18] and studies in which medical supplies are provided as part of routine service delivery might therefore, strictly speaking, be classified as health system interventions. Given these complexities, some months into the study, a decision was made to exclude studies that reported only on utilisation or other features of routine services. Difficulties defining variables in the protocol made it harder for data extractors to apply these codes and to standardise their work. One extractor based in Mexico summed this well: ‘*sometimes in reviews it is not easy to follow “the rules”’.*

**Table:** 

*‘The only difficulty I found was keeping abreast with the changes in the protocol, and due to this, sometimes the inclusion and exclusion criteria were not always clear. Whilst recognizing that flexibility needs to be maintained, for next time, it would be better to avoid changing inclusion and exclusion criteria, data to be collected etc.’*
Australian public health practitioner

It quickly became clear that the initial protocol did not cover all the complexities and ambiguities that arose, and some protocol amendments were required over time. The coordinator felt he had to balance the need for finalising the data extraction codes, with getting the review underway, and thereby taking advantage of the initial enthusiasm of the team. Participants in the review recognised the difficulties in developing a ‘perfect’ protocol, with a junior researcher in South Africa acknowledging that: *‘I believe some ambiguity is expected when working with an unfamiliar research design’*. The large majority, however, indicated they would have preferred the protocol and extraction codes to have been completely finalised before the project commenced. Several participants across the team held that changes in variables to be extracted *‘took some adjusting’, ‘affected the screening’* and ‘*would slow one down’.* The introduction of new sets of data extraction codes, such as codes for identifying articles on specific topics of interest to WHO guidelines, was even seen as altering the aims of the review. For one team member who had been involved from the very outset of the project, these new codes *‘almost took over the review’*.

Ultimately, much of the difficulty with coding came down to striking a balance between wanting more detailed data (which required more extraction codes), and the desire for having only a few simplified codes, which would be easier to standardise and quicker to extract*.* Similarly, one has to weigh up collecting information on a diverse range of issues and thus securing a larger breadth of information and outputs, against the alternative of having more codes on fewer topics, with greater depth of investigation. One respondent from the Mexican MASCOT partner supported the former approach: *‘In my opinion, the diversity of codes is what made it possible to obtain sufficiently rich outputs’*. However, in particular, the health systems experts in the study felt differently. In their view, using broad categories (for example simply labelling a study as covering “human resources”), only provided superficial insights. As a result, they developed more detailed variables and went back to extract additional data from articles classified as “health systems” papers.

The majority of reviewers had a background in health systems, rather than in maternal health or health promotion. Lack of familiarity with a topic made coding more difficult and time-consuming; one reviewer with a background in clinical medicine noted, for example, that: *‘codes for health promotion were very difficult to work with, given my limited understanding of that area’*. Many argued that papers on specific topics, such as HIV or health promotion, should have been assigned to small teams of people, based on topic knowledge and interest: *‘In my opinion, it is better if each team that has a research topic, creates the codes and leads the data extraction’*, reflected a Colombian masters graduate.

Finally, several members of the team worked full-time on screening and data extraction for a few months, which perhaps raised the quality of their work. One said: ‘*I was able to dedicate my attention fully to the review of articles, immerse myself in the topic, take breaks as appropriate and develop a consistent frame for reviewing articles’.* In her view, ‘*others who only reviewed articles sporadically might not have had the chance to develop that consistency and familiarity with the approach and/or the extraction tool.’* She also felt that having several people working full-time would allow: *‘those individuals to work closely together, with frequent contact and discussions of difficult or ambiguous papers, with other team members doing quality checks, etc., and might increase quality.’*

## International collaboration and capacity building

The review team consisted of 15 members, drawn from eight countries across five continents. The team drew on collaborators from two research projects (MASCOT and MHSAR; Table 1). Ensuring a common understanding of the project’s purposes and processes across the team was difficult, as, by design, the project had several overlapping objectives and multiple outcome measures. Conceptualisation of the project itself also evolved over time. A junior researcher in South Africa recounted that: *‘for me, with time everything became clear’,* while others felt differently, for example, a team member from Colombia said: *‘At the beginning, the objectives, tasks were clear; but at the end I was a little confused, especially about the coding and final goal.’*

**Table:** 

*’..the end product was a little different from the initial aim’* South African researcher
*‘I saw it as an excellent opportunity to collaborate with an interdisciplinary team in a unique way – having never met most of my colleagues on the review team, but forming relationships nonetheless’* Consultant who did health promotion coding

**Table 1:** Partners involved in the mapping

The systematic mapping was initially conceptualised as part of the MASCOT project. However, it soon became apparent that the scale of the project exceeded the resources available in MASCOT. The mapping team thus partnered with another multi-country research project (MHSAR), which had some overlapping objectives. Towards the project end, the team linked with the WHO Department of Maternal, Newborn, Child and Adolescent Health, who were about to embark on a series of systematic reviews to support WHO’s guidelines on health promotion interventions for maternal and newborn health.
The MASCOT project, supported by the European Commission’s FP7 research programme, included countries from Europe, Africa and Latin America (http://www.cohred.org/mascot). It consisted of 5 research institutions, 3 university groups and an NGO, representing 11 countries. The overarching aim was to identify and share country-specific strategies for tackling inequalities affecting maternal and child health (MCH). MASCOT also aimed to stimulate knowledge transfer and exchange mechanisms between and within countries for shaping policies, programmes and health actions intended to remediate MCH inequalities. Finally, the project identified coordinating mechanisms for South-South and North-south collaboration, specifically those that examine MCH status, national health research systems’ capacities and best practices, and research supported MCH strategies.
Funded by the Netherlands Organisation for Scientific Research (NWO/WOTRO), through its Global Health Policy and Health Systems Research programme, the Maternal Health and Health Systems in South Africa and Rwanda research project (MHSAR) aims to synthesize and generate knowledge on how health systems strengthening can improve maternal health, and which health system initiatives have the largest impact on maternal health. The project consortium includes research centers in two universities in South Africa, one in the Netherlands, and the Ministry of Health in Rwanda.

Including both the MASCOT and MHSAR teams in the project provided the resources needed to complete the mapping, and widened the project’s scope beyond its original conceptualisation. The coordinator was the go-between for the two consortia, while many respondents indicated they would have preferred more direct communication across the teams. This might have reduced ambiguities about “*how the two projects’ interests were being addressed by the review’* (Mexican researcher at a leading Public Health Institute) and *‘what each consortium contributed to the study’* (South African researcher employed by both projects). The later joining of staff working on the WHO guidelines on health promotion interventions for maternal and newborn health meant that three groups participated in the final stages of the project. A team member of the WHO component felt that overall: *‘most people didn’t really understand the whole of what was happening.’* While clearly there are potential pitfalls in expanding the review team, new people brought onto the project often provided bursts of energy and additional expertise.

Communication among the diverse and geographically dispersed team posed challenges. Conference calls were generally held monthly and five face-to-face meetings took place (three among the MASCOT consortia and two within MHSAR). Most felt that conference calls should have been more frequent (even weekly) and should have covered: *‘common problems’;* ‘*the lessons learnt by people reviewing abstracts’;* and ‘*the more subjective points of the review-we were still at some points discussing “what is an intervention?” late in the review’.* Additional calls could also have served to: *“push people to work more efficiently on meeting their deadline’;* and would *‘have been great for debriefing moments and helping to assist each other’.* Respondents believed that face-to-face meetings, where screening and extraction codes are applied together, might have been particularly useful. One respondent who worked full time on the review for a few months neatly summed this point*: ‘When we did have these calls I found them helpful and I think they increased the quality of the review’.*

Management of the database, screening for eligibility and data extraction were done using web-based systematic review software (EPPI-Reviewer 4, http://eppi.ioe.ac.uk/cms/). Contact between team members mostly took place through the software, with one respondent from Colombia noting that *‘the software was our office, our working place, and it was the place for closest relationship with the team members’.* The EPPI support team provided timely inputs to resolve a few minor glitches which occurred with the software. Aside form some software updates, project activities were never disrupted by website or software issues.

Although the nature of the study meant that much of the work was done by individuals working alone, ironically, the collaborative nature of the work was seen as a major strength of the study. The opportunity to collaborate drove many to join the study. A researcher based in Mexico, for example, noted that he *‘joined maybe mostly because of the diversity of participants from several countries and continents’*. Not everyone, however, felt part of a larger team. One explained that *‘I felt like I was part of a team, but only in relation to certain individuals (about four). We had more frequent contact and I felt I could go to them with questions or ambiguous papers’.* A MASCOT coordinator similarly noted that: ‘*Even though it was a team, we were from so many different countries working individually, it was hard to have a “team spirit” all the time.’* Finally, meeting the people who had been ‘virtual’ colleagues for some time was a highlight of the project for some. A South African researcher captured this sentiment well: ‘*The climax was the Mexico visit, where I got to meet in person people that I had been “virtually” working with for over a year’.*

Maintaining momentum of the study and team cohesion was relatively easy in the initial stages of the project, as these provided many opportunities for teamwork and interaction, especially with tasks done in duplicate. A lead researcher from the Mexican MASCOT partner felt that: ‘*The abstract screening was for me a most exciting process, particularly when it came to interacting with my fellow reviewers and the review coordinator.’* Participation diminished in the later stages, particularly during data analysis and dissemination. Some even felt excluded during these phases and on hindsight they felt that more efforts should have been made to secure their involvement throughout. This might also have improved the quality of the study outputs, as noted by the project lead in Costa Rica: *‘The analysis could be improved with more systematic discussions with co-authors.’*

### Building capacity of staff

Each stage of the study presented junior researchers with opportunities and scope for learning, encompassing both aspects of review methodology and content knowledge. Skill level and suitability for each stage were hard to predict and many acquired new skills by taking on tasks they had not done before. The vast majority of the team had not been involved in similar studies before; one public health graduate reflected that: *‘this was my first time being part of a ‘systematic review’ and so I was always eager to learn. It was sometimes challenging to keep up with other participants in other parts of the world, who seemed to work faster than I did.’*

**Table:** 

*‘In terms of the overall project - I think it built a lot of people’s capacity‘(*researcher involved in stage 1 and 2 of project)
*‘Made good friends and good contacts - and it has been good for my career’* (London-based researcher)
*‘Not only about research methodology, database searching and screening, but also about maternal health’* (information specialist)
*‘So much that one can learn; patience and different skills e.g. the software’ (graduate student)*
*‘I saw it as an excellent opportunity to gain exposure to a wide breadth of recent maternal health literature’* (graduate student)

Using the software also constituted a considerable learning curve for team members. Its apparent complexity even deterred some people from participating in the review. The overall MASCOT coordinator explained: *“Maybe some people did not understand the software and did not commit”*. Ultimately, team members’ proficiency and affinity with the software rose over time. A Mexican researcher recounted that: *”Sometimes issues of the software gave me grief, but it was interesting, and at a later stage I was able to assist other people’.* Another reviewer based in the United States said: *“I now feel confident in using data extraction software and was able to go on and create another review using EPPI Reviewer shortly afterwards*”. Finally, a Colombian graduate student noted that: *‘I am now conducting a systematic review and I miss this software so much’.*

Screening and coding took up a disproportionately large amount of time compared to data analysis, limiting opportunities for capacity building during the stage of conceptualising and completing articles. One researcher noted that: ”*The ratio between time on coding and time on analysis was always going to be a challenge given the time needed for coding.”* Of note, one article was led by a doctoral student [19], working closely with a few members of the team. A more planned and structured approach to capacity building might have raised participation in article writing. Finally, three team members commenced their doctoral studies shortly after the review. One South African said: *“I had no idea what exactly I wanted to look into in my PhD. The more I got involved* [in the mapping study]*, I slowly started thinking about what research ideas I was interested in.”* Another, also from South Africa, noted that it” *was a great project to be involved in before my PhD”.* She went on to say that *“as the review unfolded, I became really interested. For me, it was a way of familiarizing myself with health systems literature as this was a new field for me, which I went on to pursue in my PhD studies”.*

## Was the project worth it?

Without exception, respondents viewed the volume of work involved as large. In addition to the 10,881 titles and abstracts that were reviewed in duplicate, 4,175 full text papers were assessed for eligibility. Locating and uploading of the full text articles was particularly arduous. The final mapping entailed data extraction on 17 variables from 2,292 full text papers. A South African masters graduate captured this well: *The sheer volume of the work overwhelmed many, I think. There was a time where we were screening endlessly with thousands more abstracts to go. It did feel like a mission impossible’*. Overcoming the challenge of the work, however, and gaining new skills was viewed as an important outcome in itself. A Spanish-speaking team member from Colombia noted: ‘*I face my fears of language barriers, methodology and way of working’*.

Overall, most felt that the balance between the amount of work done and the project’s outputs was favourable (Table 2). A member of the Mexico group summed these sentiments as: *‘To me, the long time spent in what can be seen as a repetitive kind of work, was never something tedious or annoying. The outputs definitely make it worthwhile’.* For many, the largest impact of the study was its contribution to the WHO guidelines on Health Promotion interventions for maternal and newborn health. One team member, who assisted mainly with screening of articles, felt that the project’s most important contribution was the bringing together of published literature: *‘I think the outputs are well worth the investment…so much is published and if not brought together in a coherent way, what is the point?’* The overall coordinator of the MASCOT project held the view that the study’s value was its relevance around the world: “*I think these findings might be relevant for all regions, as they bring light to many issues”.*

**Table:** 

*The dissemination of results and use of findings is key, same as use of research results for decision making. As with all research, one needs to consider and prioritise: ‘The question here is HOW to better use these results? Who is the audience? How to reach them?’ How to make sure the current database is visible, disseminated and research and other institutions use it’*
Leading policy maker in Latin America

**Table 2:** Study outputs

• Symposium in South Africa involving about 100 people
• Policy Brief circulated widely in South Africa
• Open-access database of all included studies and data extracted, intended for use by other researchers [20]
• Three articles published on the findings of the mapping [19]
• A thematic series in Globalisation and Health, containing all articles from the mapping, a complementary mapping of literature in high-income countries [21] ; and a series of editorials [22]
• Mapping contributed to the systematic reviews done to support the WHO guidelines on Health Promotion interventions for maternal and newborn health [23]. The mapping identified articles on specific topics, for example maternity waiting homes, and these then formed the basis for the systematic reviews on those topics. Mapping contributed to the evidence summaries for eleven of the twelve recommendations in the guidelines
• The mapping methodology and findings were presented at two meetings at WHO headquarters in Geneva, Switzerland
• Systematic reviews that draw on articles identified on specific topics in the mapping, such as on male involvement in maternal health and birth preparedness [24–26]

As with much research, however, it is difficult to quantify how much this work adds to the existing knowledge base, and to changes in local or international policy. When asked if, overall, the review outputs were ‘worth it’, the Dutch coordinator of MHSAR replied: ‘[it was] *an enormous time investment for a large group of people; but we should all realise that there are other benefits and spin-off effects, beyond the few journal papers that have resulted from this work.’* Another reviewer from South Africa, who commenced masters studies in the United States after the project, felt its principal contribution centred on the novelty of its outputs: ‘*I feel that the novelty of the project definitely adds a different dimension to the maternal health research landscape’*. The team also believed that the methodology of the mapping was novel and would help to advance this kind of technique and inform similar studies in the future.

## Conclusions

The paper sums the perspectives and experiences of the mapping team on the methodology, collaboration and learning opportunities provided by the study. Of note, several factors were identified which could affect the quality of a mapping study and thus the validity of its conclusions. Importantly, the selection and definition of the mapping variables should be finalised prior to onset of the screening, as far as possible, even if that delays the start of the study. Other factors that may influence quality include difficulties in locating non-English literature, lack of familiarity with the subject matter of the mapping, and limited communication and cross-learning among the team.

Communication and coordination of a team spread over five continents was difficult and similar projects might consider having smaller teams working full time, and more frequent face-to-face meetings and conference calls. During the early stages of the study, levels of collaboration were high, though more regular and structured communication was needed. The project was notably less successful at securing participation and learning in its later stages, specifically during data analysis and dissemination. Moreover, there was little interaction between the MASCOT and MHSAR project teams. Disappointingly, no new projects have yet been developed between the research entities involved in this collaboration.

Despite its challenges, the overall predominant view of the team was summed by a leading policy maker in Latin America as: *‘The joint international effort was a great experience, a new line of research could have started and maybe someone would follow.’* Though acknowledging the considerable volume of work involved in such a mapping, the team gave a favourable assessment of the balance between the amount of work required and the value of the study outputs. Also, the study offered a wide range of opportunities for capacity building, both in terms of learning about some aspects of systematic review methodology and software, and in obtaining broad knowledge in the field of maternal health. In these areas, the study seemingly achieved its aims, even though capacity building was less successful during data analysis and dissemination. The series of articles summing the mapping findings and the mapping’s role in identifying the studies that were then included in the systematic reviews for WHO guidelines were especially valued.

Two features of the underlying design of the mapping study bear mention. The exclusion of grey literature makes it difficult to claim that a mapping sums all research on a topic. Also, the lack of assessment of the quality of the studies included in the mapping, as done in systematic reviews, influences the validity of the study’s conclusions. Without assessing the quality of the studies, those with weak and strong methods are given equal weighting. Proxies for study quality were used in the mapping, such as study design and the Impact Factor of the journal in which an article was published, but these cannot replace a formal assessment of the quality of a study. A mapping or scoping of literature is also not a necessary precursor to a systematic review, and may not be an efficient means of doing so, given the burden of work involved.

It might be useful thus to conceptualise mapping studies as synthesizing a body of literature, as distinct from an evaluation of literature as is done in a systematic review. A mapping, such as this study, aims to provide detailed information about the nature of a research field and to investigate a wide range of issues. This, however, means that mapping studies run the risk of being construed as unfocused as, by their nature, they have broad, sometimes difficult to define objectives. Moreover, having such a large team, with members drawn from three projects, poses serious challenges in ensuring a uniform understanding of the protocol, the coding process and intended outputs. The desire to gather a wide breadth of information, as opposed to depth on a few topics, heightens the challenge of clearly delineating the objectives of the mapping.[8] Another mapping study had a similar experience: as that team grew increasingly familiar with the literature being mapped, it became necessary to clarify the study concepts and to revise its research questions [1].

Essentially, the value ascribed to the project stemmed principally from the project’s novelty and contribution to evidence, and its collaborative and capacity building opportunities. The validity of the outputs, however, is tempered by deficiencies in mapping methodology, especially the lack of assessment of the quality of included studies. Based on the perspectives of the review team, the practical ingredients needed to complete such a project are: a sizable team, ideally with some staff working full-time; support for locating and uploading of full text articles; and optimising the number of data extraction codes used, yet retaining some measure of depth and breadth of the data obtained. In addition to these factors, prerequisites for a successful mapping include: strong collaboration across the team, a shared understanding of the review purposes, and standardised screening and data extraction procedures across the team. We conclude that, with all these elements in place, and with sufficient focus and funding for dissemination, mapping studies can make useful contributions to the literature and to building the skills of research teams.

## Additional files

Additional file 1: Tool for gathering perspectives of participants on mapping methodology.ᅟ(DOCX 20 kb) Additional file 2: Mapping search strategy and protocol. (PDF 1497 kb)

## Abbreviations

LMICs: Slow- and middle-income countries
MASCOT: Multilateral Association for Studying health inequalities and enhancing North-South and South-South Cooperation
MH-SAR: Maternal Health and Health Systems in South Africa and Rwanda
