# Supplementary material for: Perspectives on the methods of a large systematic mapping of maternal health interventions
Source: Global Health. 2016 Aug 25;12(1):51. doi: 10.1186/s12992-016-0191-7 (PMC5000454; doi:10.1186/s12992-016-0191-7)
Supplement: Additional file 1: — Tool for gathering perspectives of participants on mapping methodology.ᅟ(DOCX 20 kb) [file 12992_2016_191_MOESM1_ESM.docx]

**Additional file 1**

**Commentary on methods used in MASCOT/MHSAR review and HIC review**

**Authors of commentary**: those who provide a detailed thoughtful contribute to the commentary. The order of authors based on contribution to the commentary, and number of screening/extractions done in the review

**Proposed word count:** about 2500 words

**Scope of commentary:** Both the MASCOT/MHSAR and HIC review to be included. The commentary will sum the methods we used in the reviews, and the experiences of the review team (strengths and weaknesses of the review). We will compare our methods to studies that used similar mapping methods. Mainly focus on the perspectives of the review team.

**Methods for writing the commentary:** make a summary of the review protocol and review outputs; collate reflections from the review team (also from those who did not join, or started the review and discontinued participation); locate studies that used similar methods and compare to our approach; prepare some concluding remarks.

**Instructions for completing your reflections**: You do not need to address all the questions below; they are only a guide. You can raise additional issues that are not covered below. Please be critical, do not be afraid of highlighting any problems you experienced. We want to note both the strengths and problems. You do not have to be polite. We can easily arrange a system of sending comments anonymously if you wish, just let me know what works for you. You can give several responses to each item below.

**Questions on your perspectives**

| **Item** | **Issues to consider** | **Please enter your perspectives here (use as much space as you wish)** |
| --- | --- | --- |
| Overall perspectives | 1. Overall, what is your experience of participating in the review? 2. What could have been done differently in the review? 3. Overall, what parts of the review worked well? What parts worked less well? (protocol, abstract/title screen, full text extraction, analysis and dissemination, or) |  |
| Participation in the review | 1. Many people on Mascot and MH-SAR did not join the review, or left the review before the end. Why do you think that happened? 2. What motivated you to join the review (e.g. because it was part of my commitment to the project; the review sounded interesting; the possibility of being part of publications, or)? |  |
| Quality of the work | Please comment on the quality (robustness, validity of the work) of:   1. Do you think the literature search obtained most of the relevant literature? 2. Abstract screening and full text extractions? 3. Analysis and dissemination of results (journal articles and other dissemination)   How could the quality of the review have been improved? |  |
|  | 1. How could the quality of the review have been improved, which aspects in particular? |  |
| Review objectives and aims | 1. At the beginning of the review, were the objectives of the review clear? Did you understand at the beginning what the task would entail, or did it only become apparent to you over time? At the end of the review, were you clear on whether the review achieved its objectives? |  |
| Was it all worth it? | 1. What did you learn or gain from taking part in the review? 2. Considering the length of time spent on the review and the repetitive nature of the work, do the outputs make this all worthwhile? |  |
| Functioning of the team and communication within the group | 1. Did the participants in the review work together as a team who together were tackling a large task, or did it feel like there were several individuals working separately, not really knowing who was doing what, or when? 2. How could the review leaders have improved their coordination of the review 3. Overall, were the protocol, study processes, coding, EPPI software and end goals of the review clear to you? |  |
| Coding processes | 1. How did you find the EPPI software. How could it be improved? 2. Comment on the codes we used during data extraction from the full text articles, too many codes, e.g. too few codes, too complex to understand. Which codes worked well, or badly? Would you have added any other codes? |  |
| Non-English literature | 1. How well did the screening and extractions of non-English papers work? Do you think there were any differences between the methods used for English versus non-English papers? Do you think that we captured the non-English literature on the topic well? 2. For participants for whom English was not their first language, did you feel adequately included in the review and dissemination. What could have been done to enhance inclusion, for example, translating the protocol and codes into other languages? |  |
| Dissemination of findings | 1. Please comment on the dissemination thus far of the findings of the review, where and how we have disseminated so far (journal articles, EU, reports etc.). The time between doing the review and dissemination? Did we miss any opportunities for dissemination? 2. Did the number and quality of the journal articles so far match your expectations? 3. How much do you think that the review ultimately adds to existing knowledge on the topics we assessed? |  |
