# Supplementary material for: Perspectives on the methods of a large systematic mapping of maternal health interventions
Source: Global Health. 2016 Aug 25;12(1):51. doi: 10.1186/s12992-016-0191-7 (PMC5000454; doi:10.1186/s12992-016-0191-7)
Supplement: Additional file 2: — Mapping search strategy and protocol. (PDF 1497 kb) [file 12992_2016_191_MOESM2_ESM.pdf]

Systematic review protocol

Review title: Health system and community-based interventions for improving maternal health and for reducing maternal health inequalities in low- and middle-income countries: a two-stage mixed-methods research synthesis

This review falls within the European Union Mascot project and the Netherlands-funded Wotro project

Version 6.2 February 2014

Contents

Acronyms 4

Document structure: essential reading for Stage 1 reviewers 4

Definitions and key concepts in the review 4

1. Background 5

1.1 Systematic review of the impact of health system interventions on maternal health within the Wotro project 5

1.2 Systematic review of equity effects of maternal health interventions within the MASCOT project 6

Link with previous work packages in Mascot project 7

2. Overview of methods for the Wotro and Mascot systematic reviews 8

3. Review conceptual framework 10

4. Literature search strategy 13

5. Eligibility criteria for Stage 1 14

Inclusion criteria for Stage 1 of MASCOT/WOTRO review 15

Exclusion criteria for Stage 1 of MASCOT/WOTRO review 17

6. General instructions for Stage 1 screening of titles, abstracts and full text 17

7. Screening of titles and abstracts in Stage 1: variables and instructions 18

Instructions for coding on title and abstract 19

8. Screening of Full Text articles in Stage 1 21

Notes on included articles at the full text stage 22

Instructions for background articles 23

Instructions for duplicate articles 23

Full text codes for screening, included studies 23

Full text codes for screening, excluded studies 25

9. Extraction of data on Full Text articles in Stage 1: mapping of interventions for tracer conditions and health systems 26

9.1 Full text extraction for mapping clinical tracer conditions in Paper 1 29

Full text screening instructions and definitions of variables for clinical intervention studies 29

Definitions of variables to extract 30

9.2 Stage 1: Data extraction on health systems interventions for maternal health 34

Instructions for reviewers 34

Variables to be extracted from all health system and community-based articles 37

9.3 Stage 1: Data extraction on health promotion interventions for maternal health 38

10. Stage 2: In-depth systematic reviews for Wotro and Mascot projects 39
Further detailed review questions and analysis 42
Approach to data analysis in stage 2 of Mascot 45
11. Review limitations and strengths.. 46
12. Review team roles and responsibilities 46
Review timelines and milestones 46
Stage 1 Timelines 46
Stage 2 Timelines 50
Stage 1 Mapping specific tasks 50
Annexes... 54
Annex 1: Search strategies and interim results of literature searches in stage 1 54
Interim results.. 54
PubMED search strategy.. 56
CINAHL search strategy... 57
Embase search strategy 60
PsycINFO search strategy... 61
Web of Knowledge search strategy 62
Annex 3. Study resources.. 66
Annex 4. List of low- and middle-income countries 68
Annex 5. List of key coding examples 70
References 76

Acronyms

**CF**	Conceptual framework
**CHP**	Cente for Health Policy
**HIS**	Health information systems
**IP**	intrapartum
**LMICs**	Low- and middle-income countries
**MASCOT**	**M**ultilateral **A**ssociation for **S**tudying health inequalities and enhancing North-South and South-South **CO**opera**T**ion
**MCH**	Maternal and child health
**MH**	Maternal health
**PHC**	Primary health care
**PP**	postpartum
**PICOT**	Population, Intervention, Comparator, Outcome, Time
**PROGRESS-Plus**	Place of Residence, Race/Ethnicity, Occupation, Gender, Religion, Education, Socioeconomic Status, and Social Capital, and Plus represents additional categories such as Age, Disability, and Sexual Orientation
**TI/AB**	Title abstract
**WOTRO**	Science for Global Development', part of the Dutch Organization for Scientific Research (NWO)
**WP**	Work package

Document structure: essential reading for Stage 1 reviewers

All people who will carry out coding of titles and abstract or full text in Stage 1 must carefully review the following sections before they begin coding: Definitions and Key concepts in the review; Section 2; Section 5 (to be read twice); Section 7; Section 8; Annex 4; and Annex 5. These are considered essential reading for all reviewers. The remaining sections provide background and explain the subsequent steps in the project.

Definitions and key concepts in the review

Multiple/Complex intervention. Provision of a set of clinical interventions, as opposed to provision of a single clinical or laboratory intervention. These studies mainly are assessments of service delivery(1).

Health systems. In the review, we will categorise health systems interventions as follows:

1. **Service delivery:** packages; delivery models; infrastructure; management; safety & quality, integration of care; adherence to treatment protocols; standards; licensing; certification; and accreditation
2. **Health workforce:** national workforce policies and investment plans; advocacy; norms, standards and data; and training.
3. **Information:** facility and population based information & surveillance systems; global standards, tools
4 **Medical equipment, infrastructure, products, vaccines & technologies:** norms, standards, policies; reliable procurement; equitable access; quality
5. **Financing:** national health financing policies; tools and data on health expenditures; costing; risk sharing/pooling; insurance; protection; and purchasing
6. **Leadership and governance:** health sector policies; harmonization and alignment; oversight and regulation; and support services such as standards and norms
7. **Demand-side interventions**, including community education; community needs, involvement, participation, responsiveness ; and male involvement

The review will also assess relationships between the individual building blocks items (listed as 1-6 above) and how these components interact with each other, and with patient demand.

Maternal health. Classified as pregnancy, childbirth and the postpartum period (defined as the first two years after childbirth). Fertility treatment is excluded. Only family planning services specifically provided for women in the postpartum period will be included, not other family planning services. Women of all ages are included in this review, including adolescent women.

PROGRESS-Plus. The review uses this acronym to define disadvantage, the key nexus of social stratification. These categories are: Place of Residence, Race/Ethnicity, Occupation, Gender, Religion, Education, Socioeconomic Status, and Social Capital, and Plus represents additional categories such as Age, Disability, and Sexual Orientation. The acronym PRORESS-Plus is used by the Campbell and Cochrane Equity methods Group and the Cochrane Public Health Review Group.

Joint Wotro and Mascot review The review will be done as part of Wotro and Mascot projects (see below), the first stage of these reviews (identifying and mapping the literature) is identical in both projects and will thus be done together. The second stage of the reviews (addressing specific review questions in detail) will likely differ between these two projects, though possible sharing of tools will be considered, such as data extraction tools.

1. Background

1.1 Systematic review of the impact of health system interventions on maternal health within the Wotro project

The systematic review within the Wotro project will examine evidence of the impact of different supply and demand initiatives on maternal health in LMICs; how potential synergies have been exploited in diverse contexts, which system developments are most critical for maternal health, and what effects other health systems strengthening initiatives have had on maternal health.

The review will thus provide practical insights into how, in the past decade, "systems thinking" has been operationalised within maternal health programmes and research. This entails mapping the published evidence about how health system components have been applied within maternal health programmes or projects in LMICs, how they have been implemented and with what outcomes. This review, as with the subsequent three sub-projects of the Wotro project, adopts a broad approach to systems thinking, extending beyond the six WHO building blocks, with the inclusion of demand-side initiatives for example. The review team will pay particular attention to assessing relationships between the individual building blocks and how these components interact with each other, and with patient demand. In the review, special attention will also be given to locating evidence that might explain why and how a system intervention worked.

Overall, the review aims to systematically identify evidence of the impact of health system interventions on maternal health. The review also aims to identify factors or competing phenomena within the health system which might affect the impact of maternal

Overarching PICO question for Wotro review

Population: women in pregnancy, childbirth or first two years postpartum

Intervention: public health interventions (health system change or complex intervention)

Comparator group: those not receiving the intervention

Outcome: effects of the intervention on Maternal Health outcomes

health programmes or services. This will thus involve evaluating which programmes or services, or the design or implementation thereof, are associated with more beneficial outcomes. During this review we will also identify illustrative countries and case studies for more detailed study in the subsequent study sub-projects.

This review uses a health system framework, encompassing the seven intervention areas described in the definitions and concepts section above.

Specific objectives of the Wotro review:

1. To systematically identify evidence of the impact of health system or multiple clinical interventions (packages of care or complex interventions) on maternal health
2. What does available evidence show about the extent to which maternal health can be improved through interventions to strengthen the health system building blocks, synergies between them, or to enhance patient demand?
3. What externalities and unintended consequences have occurred from health system interventions to improve maternal health?
4. Is there any evidence that some system interventions are more effective than others in particular contexts?

1.2 Systematic review of equity effects of maternal health interventions within the MASCOT project

The main objective of the MASCOT project is to stimulate cooperation between countries from Europe, Africa and Latin America, to identify and implement country-specific strategies for tackling health inequalities affecting mothers and children. Ultimately, these strengthened collaborative actions aim to reduce inequalities. The review done within Mascot aims to systematically identify strategies for tackling health inequalities affecting mothers.

The systematic review within MASCOT will incorporate input from the whole MASCOT team, and from other relevant stakeholders, including users (MH service users, researchers and policy makers). The review team and stakeholders will together define the final research question(s), the conceptual framework for the review and other review outputs. The review, done as Work Package 5 of the MASCOT project aims to strongly complement activities in other Work Packages, and to identify best practices and principles.

A systematic review examining equity impacts of a health system or package of clinical interventions (multiple or complex interventions) is challenging, given the complex nature of the processes of policy implementation and programmes. These processes make it hard to determine the dynamics of interventions with precision, and to definitively identify the factors that influenced effectiveness of interventions, and its differential impacts. Systematic reviews seldom consider effects on health equity (petticrew 2004, ref in Welch). As opposed to other reviews, equity-focused reviews require a deeper investigation of primary studies, with a greater consideration of the implementation processes and context, and of the quality of studies.

This review will show the potential for differences in relative effects of maternal health services between advantaged and disadvantaged populations. By documenting the distribution of benefits and adverse outcomes of MH interventions, across socio-demographic groups, the review will detect best practices which can reduce disadvantage. These findings will be used to inform WP-6 of Mascot.

These best practices are interventions that are especially effective at improving MH outcomes of the poor and other vulnerable groups, and have reduced overall MH inequalities in the studies reviewed. The review also seeks to identify the determinants of (factors which might explain) the relative equity effects of programmes or services for maternal health. In summary, the review involves examining associations between the characteristics of interventions and the effectiveness of interventions, as measured by equity gains.

The review adopts the approach that disadvantage can be measured across categories of social differentiation, using the mnemonic PROGRESS-Plus (Evans, 2003 and Oliver, 2008). PROGRESS is an acronym for Place of Residence, Race/Ethnicity, Occupation, Gender, Religion, Education, Socioeconomic Status and Social Capital, and Plus represents the additional categories such as Age (Adolescents) and Disability. Not all these categories will be relevant in this review. The review will thus examine the extent to which programmes or sets of services for improving maternal health have had differential effects on advantaged or disadvantaged groups, for each of the dimensions across which health inequalities might exist (the PROGRESS-Plus groups).

Given that the Mascot project covers only two regions of the world, in each paper, we will attempt to take other contextual factors into account, such as the overall country characteristics and particularly health system characteristics. While the PROGRESS categories capture the population under study, in the final analysis we may stratify countries by statistics such as a country's income, life expectancy and Human Development Index (HDI). Furthermore, health systems will vary in terms of horizontal and vertical equity of health interventions provided to all the population. The review will thus group types of research and particularly types of interventions and outcomes by HDI and health system characteristics. Countries could also be grouped according to financial protection, though out-of-pocket health expenditures or catastrophic health expenditures. The harvest plot could then show these variables as contextual determinants of the interventions and outcomes.

Link with previous work packages in Mascot project

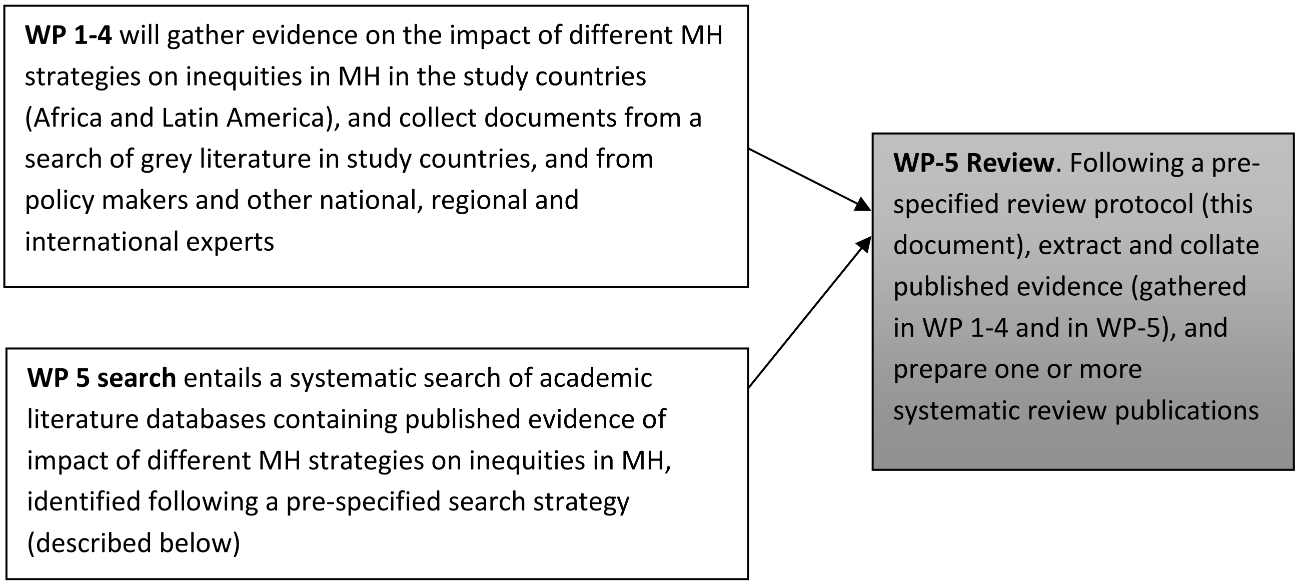

Overall, the review aims to determine whether some interventions led to a decrease in the measurable health experiences and outcomes, according to the PROGRESS-Plus categories. The

review also aims to identify factors or competing phenomena within the health system which might affect the impact of the programmes or services on different population groups. This will thus involve evaluating which programmes or services, or the design or implementation thereof, are associated with more beneficial outcomes for some populations or subgroups.

Specific objectives of the Mascot review

1. To systematically identify evidence of the impact of a programme or service on inequities in maternal health
2. Identify which characteristics of an intervention and its implementation are associated with gains in maternal health equity
3. To assess the extent to which maternal health interventions are explicitly designed and evaluated to address inequities in maternal health (whether effects on equity are explicitly taken into account when designing the intervention and when evaluating or reporting outcomes of services)
4. Within maternal health studies, describe the measures of equity effects, and disadvantaged populations used in these studies

Overarching PICO question for MASCOT review

Population: women in pregnancy, childbirth or first two years postpartum

Intervention: public health interventions (health system change or complex intervention), or tracer condition interventions

Comparator group: those not receiving the intervention

Outcome: differential effects of the intervention on disadvantaged groups in the PROGRESS-Plus categories, (gradient and excluded groups)

Though the review principally aims to identify effective means of providing maternal health services which reduce inequities, it also will be used for "theory-building". In theory-building, we will explore the mechanisms that mediate between the delivery (and receipt) of the programme and its outcomes (Weiss 1998). Practically, this means identifying common mechanisms and clarifying empirical relations between the mediating factors and the main effects.

2. Overview of methods for the Wotro and Mascot systematic reviews

For this review, Maternal Health is defined as the time from conception until two years after childbirth, thus covers pregnancy, childbirth and the postpartum period. Primary evidence published in peer-reviewed literature will be systematically identified, quality assessed, data extracted into standardised data forms and overall findings collated. In addition, evidence will be drawn from existing systematic reviews of the impact of initiatives to improve health systems for maternal health, or to increase demand for such services (such as altering household-decision making)(2). Only interventions related to health system or patient demand will be included, not studies of individual clinical interventions, or descriptive studies, such as needs assessments. We will, however, include studies on interventions to address a few key clinical conditions, assessing the equity outcomes of these interventions and any differences in system factors which influence their effectiveness. All study designs used to evaluate an intervention will be eligible.

Figure showing literature located in search and screening stages of Mascot and Wotro reviews

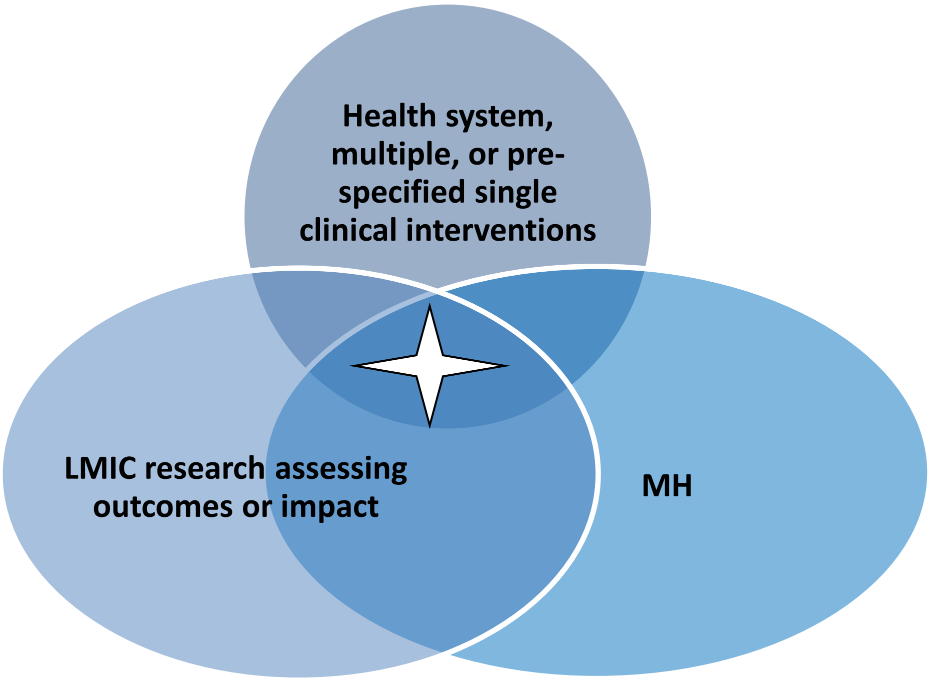

The star in the figure above indicates the relevant literature for the Wotro review (studies in LMICs that assess outcomes or impact of a health system intervention, pre-specified clinical condition, or a package of interventions (multiple or complex interventions) among a maternal health population. The Mascot review includes the same literature as Wotro, but only studies that report the differential effects of such interventions on PROGRESS-Plus groups.

The review consists of two phases. Firstly, Stage 1, which entails developing a systematic map (see Section 4 below). Stage 2 consists of individual systematic reviews (see flowchart figure below with indicative number of papers in each step). In the second stage decisions are made about what specific review questions to address. Review topics will be assigned to different members of the review team in Stage 2. Thus, this large and complex topic will most likely be divided into a series of interrelated, but distinct review questions, assigned to different sets of investigators.

The systematic review will use pre-specified methods, which are reported transparently and with sufficient detail to be replicable. Reference and data extraction tools developed by EPPI-Centre will be used (http://eppi.ioe.ac.uk/cms/Default.aspx?tabid=184). A preliminary scoping review indicated that only a small number of studies meet the inclusion criteria for stage 2, so the workload for the systematic review will be manageable within the project timeframes.

Arabic, English, French, Japanese, Portuguese and Spanish articles will be included in the review. For the Wotro project, French papers will be extracted in English by the Rwandan team, fluent in both languages. Members of the Mascot team will assist with extracting information from papers in languages other than English.

Figure showing stage of review and indicative number of papers

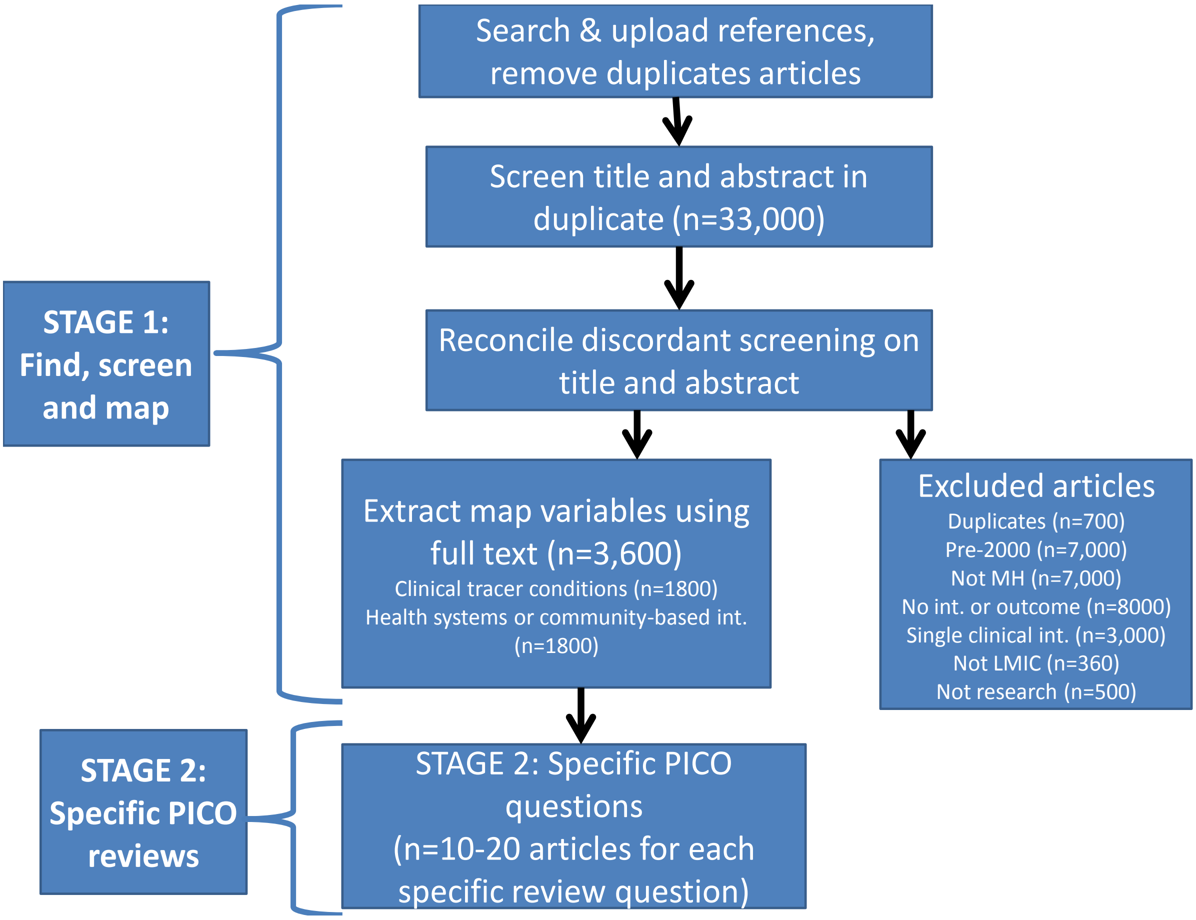

3. Review conceptual framework

The first task here, as in all reviews, will be for the research team to agree on a conceptual framework (CF), a critical element, applied throughout the research protocol. The CF defines the parameters of the study, and provides the team with a clear and transparent tool which depicts their shared understanding and knowledge of review concepts, in what is a highly complex area. The CF also informs selection of study inclusion criteria, search strategies, and development of a descriptive coding tool(3, 4). It plays a different role in each stage of the review and some incremental changes will be made to the framework as knowledge accumulates in the review.

Conceptual frameworks, which identify important elements and relationships within a system, have been used extensively in the understanding of complex programmes to improve social and health outcomes. They illustrate how a programme is designed to achieve its intended outcomes, connections between the determinants of outcomes and causal factors, and which competing factors affected the distribution of outcomes of a programme/service. The conceptual framework facilitates the process of gathering and integrating studies of health system interventions or packages of care (complex or multiple interventions), and also informs the interpretation of cumulative results. It identifies the complex links between determinants, outcomes and intervention components, and guides technical aspects of the review. Factors specified within the model may act

directly on processes of the conceptual framework, or as mediating mechanisms on the processes depicted.

Devising the conceptual framework helps delineate the conceptual boundaries of the review. Further, specifying the conceptual framework *a priori*, uninfluenced by the review findings, is intended to reduce bias in researcher judgement. The final model will depict how the interventions work in different populations, and whether mechanisms through which they work differ between populations.

In this review, the conceptual framework illustrates the conceptualisation of the review; the hypothesized causal links, and effect modifiers and mediators; intermediate outcomes; and the subgroups which are the focus of the analysis. The conceptual framework includes the hypothesized mechanism of action of each programme or service identified, that is, how the intervention is expected to work. The conceptual framework presented below thus aims to depict how factors associated with disadvantage (social stratification) might interact with the hypothesised mechanisms of action (Mascot review). A simplified adaptation of this framework applies to the Wotro review. If evidence is available, this could be contrasted with the mechanism of how the exposure actually exerted its effects, if any.

Figure 2: Conceptual framework showing the hypothesized relation between maternal health interventions, mediating factors and, overall effects (Wotro review) as well as equity outcomes (Mascot review)

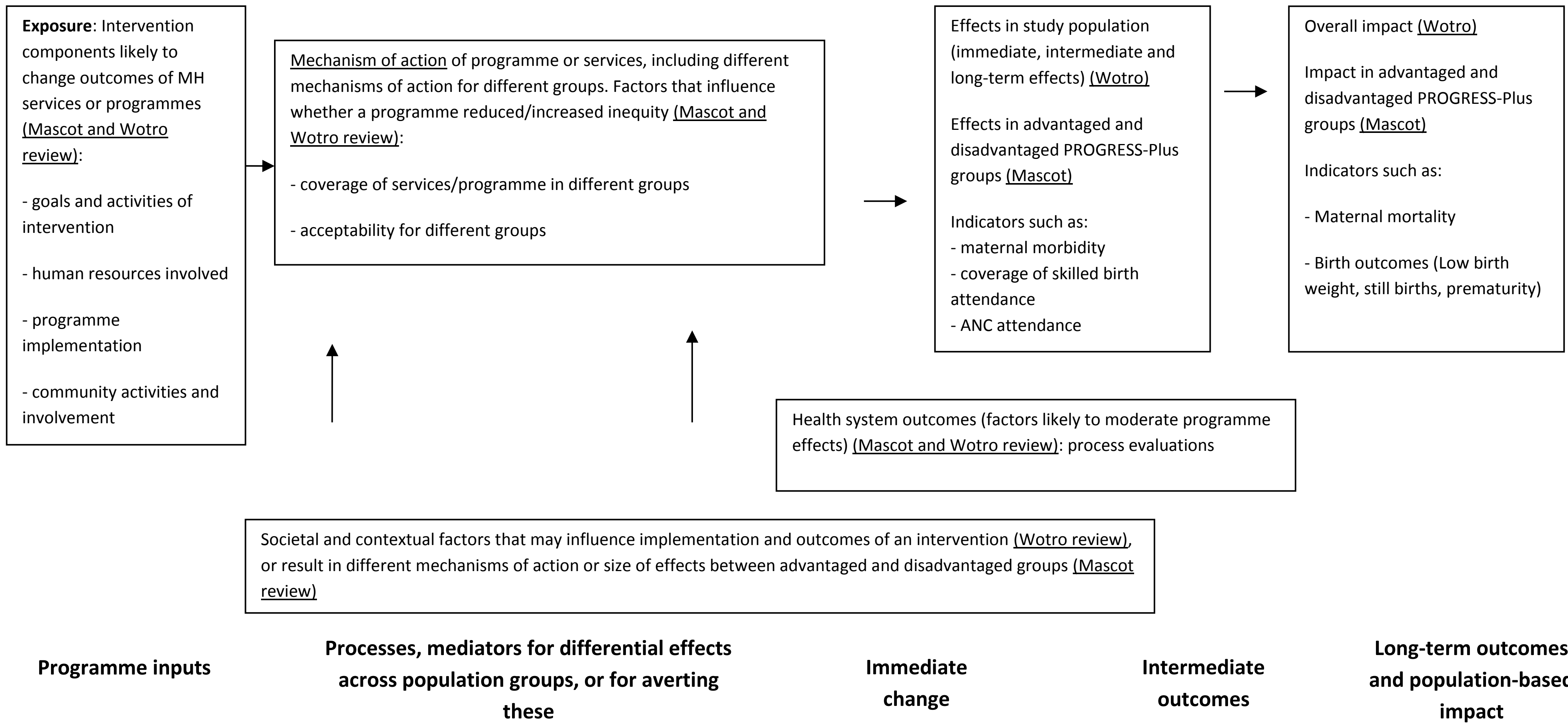

4. Literature search strategy

Tackling global inequalities in maternal health requires systematic reviews of relevance to low and middle income countries (LMIC). A major challenge is how to identify and include research literature conducted in LMIC, much of which is not indexed by the major international research databases, and can be hard to identify literature. This review attempts to address this issue by searching a range of research sources that includes regional databases and registers of research specific to low and middle income countries. Given the breadth of the potential research literature on maternal health, and the difficulties in identifying research related to health inequalities in publications, the search will be both broad and inclusive.

Data sources include both unpublished and published literature, drawn from academic and other databases, as well as from experts. Piloting searches will help to determine what research evidence to identify and the exploratory searches will assist in refining the search criteria. Search terms for pubmed and other database were finalised following piloting (filters and search limits).

Search terms

A highly sensitive search strategy using both controlled vocabulary and free-text terms to identify studies on PubMED will be developed, and adapted for subsequent searches of other electronic sources. We include search terms for maternal health, and low- and middle-income countries only. Searches will be limited to the period from 2000 to 2012 No language restrictions will be employed in searching. Search terms for maternal health will be combined where appropriate with terms for low and middle income countries as defined by the World Bank (see Annex 4). Given that findings related to health inequalities are often a sub-analysis of a study and are frequently not reported in titles and abstracts we will not included search terms for any specify categories of disadvantage (oliver et al 2008). In particular, important negative findings of sub-group analysis are less likely to be mentioned in the title and/or abstract of an article. Final search strategy is in Annex 1.

Databases

The box below shows the final list of the databases that were searched

Sources
CINAHL (Cumulative Index to Nursing and Allied Health Literature)
MEDLINE
EMBASE
PsycINFO
Web of Knowledge (Science Citation Index Expanded; Social Sciences Citation Index
PopLINE
African Journal Online
African Index Medicus
LILACS
Index Medicus for the South Eastern Region (IMSEAR)

Websites of agencies

Web sites of agencies involved in maternal health (WHO, Making Pregnancy Safer, Family Health International and Population Council, Family Care International, Plan International, Mary Stopes

International, Unicef, UN Women) may be searched. This would enable us to include evaluations of projects done by these organisations.

Reference lists

In stage 2 of the review, for some PICO questions, the bibliographies of included studies will be scrutinised to find additional studies. Reference lists of relevant systematic reviews, letters and commentaries were also examined.

Expert opinion

For WP-1-4 of MASCOT, experts in each of the MASCOT countries were contacted and asked to provide evidence that is potentially relevant to the review. The WP 1-4 will collate evidence on the impact of different MH strategies on inequities in MH, and obtain relevant documents from policy makers, and a search of national grey literature in WP-1-4, and from national, regional and international experts and key informants. All these documents will be reviewed for eligibility in the review. We will also contact experts known to us in the fields of maternal health and equity to request them to help identify additional studies which may have appropriate data, particularly if these are unpublished. Further studies identified by other means will also be assessed for eligibility using the criteria listed below.

5. Eligibility criteria for Stage 1

This first review stage will enable us to describe the proportion of maternal health literature that focuses on single clinical interventions, and the study designs used for MH systems research, as well as other key characteristics of maternal health research since 2000. This is useful information, will be collated in a scientific publication.

Overall, the review will cover public health interventions (health system interventions or multiple/complex interventions), as well as interventions addressing selected clinical conditions. Original studies on MH interventions will be included, as well as systematic reviews on MH. We will exclude the effects (or differential effects) of single clinical interventions other than the tracer conditions we selected for review. For example, we will exclude a study of the effects of iron supplementation for pregnant women. We will, however, include individual health system interventions, such as an intervention to increase the numbers of midwives, or to remove user fees for childbirth services.

Studies on the delivery of multiple interventions, such as a package of antenatal care will be included (outcomes of multiple/complex interventions) if they cover one of the selected tracer conditions. Importantly, studies reporting (differential) effects or coverage of maternal health programmes or a set of services in a district, province, or country will be included. We will include studies that assessed different ways or modes of implementing single clinical interventions. Assessment of different implementation strategies is clearly a health systems intervention.

All study designs which provide evidence to answer the review question will be included in the Stage 1 mapping. Thus, no restrictions will be placed in the selection criteria for study designs, so that all studies reporting outcomes of a health system intervention or other intervention of interest are

included, both trial and observational studies. Quantitative and qualitative studies have to include an outcome of an intervention. Those only describing an intervention and not its outcomes will be excluded. Books and doctoral dissertations were excluded.

Especially for the Mascot review, this broad inclusion is used as few randomised trials of system interventions have been done in LMICs. Even fewer include equity outcomes as the primary outcome of the trial. Moreover, much relevant information is available from studies lower in the evidence hierarchy. Previous equity-orientated reviews also included studies other than randomised trials, and drew most evidence from studies lower on the evidence hierarchy (ref). In these reviews, the observational evidence base was especially informative about the differential effects of interventions, and no trial evidence was located in some reviews (ref). Further, inclusion of observational studies fulfils the review's aims of collating evidence that corresponds to the conditions under which health policies are mostly applied in practice. Mostly these are observational studies, a synthesis of research that is applicable to drawing inferences for policy, in a policy-relevant manner.

Inclusion criteria for Stage 1 of MASCOT/WOTRO review

1. **Population included**. Interventions must target a maternal health population (*women in pregnancy, childbirth, or within two years postpartum*), or male involvement with a maternal health population, or be general health system interventions, provided they report outcomes in a maternal health population. For example, please do include a study describing a general intervention to raise the salary payment levels of all health staff, but that reports outcomes of this intervention among pregnant women. Maternal health in adolescents is included. Include articles on interventions for breastfeeding women, provided that they address maternal health, and satisfy other inclusion criteria (one of multiple interventions or a health system intervention etc.). Include articles on abortion, provided they satisfy other inclusion criteria. If the intervention is among a maternal health population, but is primarily for the benefit of the child, it must still be included nonetheless.

2. **Study outcomes included.** Quantitative or qualitative outcomes, or data on the impact of MH interventions at a population level must be reported. The intervention **must** directly or indirectly involve a maternal health population (defined immediately above), but outcomes may be measured in either the woman, or the newborn child. Biological, process, health systems and other outcomes measures are all applicable.

3. Interventions included, The following types of interventional studies are included, provided they also meet the other inclusion criteria 1, 2 above and 4-8 below, and ***provide data on study outcomes*** (utilisation of services is an outcome).

3.1 **Health system interventions included**. Studies that report ***outcomes*** of: health systems interventions for improving maternal health; other multiple/complex interventions for improving maternal health; health services research; organisation of care interventions; or outcomes of national, provincial or district-level maternal health programmes. This includes studies of socio-economic or environmental interventions, such as improving water supply. Health system interventions generally fall within the 6 health system building blocks or aim to raise patient demand for services. Interventions that aim for

general health systems strengthening (such as building more primary care facilities), but that measure the effects of this intervention on maternal health outcomes, will also be included. Single health system interventions will be included. Interventions around traditional birth attendants are classified as health system interventions (human resources building block). Case reports most often have an intervention and an outcome. An intervention can include making a diagnosis of a condition and providing treatment as part of patient management (provided it meet all other inclusion criteria). But we exclude articles where making a diagnosis is only for the assessment of burden of a condition in a population (i.e. disease surveillance or burden of disease studies are excluded, unless they aim specifically to compare alternative surveillance methods). Comparisons of different indicators of maternal health are included (information health system building block). Assessments of the outcomes of implementing clinical practice guidelines or similar guidelines are included under health systems interventions. Descriptions of clinical guidelines without any process or impact outcomes are excluded.

3.2 **Community-based interventions**. Interventions delivered in community settings (any activities occurring outside of health facilities) will be included provided they describe some outcome (including process/uptake outcomes), even delivery of single clinical interventions.

3.3 **Pre-specified single clinical interventions, as tracer conditions.** Certain pre-specified single clinical interventions are included. These are considered tracer conditions and provide relevant information for this review. Key health system lessons will be drawn from study of the effectiveness of interventions for these tracer conditions, and how such effectiveness varies across settings. For example, this will enable the review team to compare the health system requirements of malaria versus PMTCT. The conditions considered tracers in this review are those addressing maternal: HIV/STIs; malaria, hypertension, haemorrhage and pregnancy sepsis. Studies only addressing PMTCT interventions must also be included as Maternal HIV. Outcomes of interventions must be described (even process or uptake outcomes, any outcome).

3.4 **Studies describing levels of service utilisation or coverage.** Descriptions of levels of service utilisation or coverage of services are included, either of single or multiple services. This means including studies reporting population-level survey findings of associations between exposure to an intervention, such as antenatal care in different groups. Qualitative studies of service utilisation are included. These studies must be coded specifically as service utilisation in screening. If studies report an intervention to alter use of services, they **must be coded as a health system intervention, not** coded as a service utilisation study.

4. **LMICs**. Only studies in LMICs will be included. See Annex 4, LMIC countries defined by the World Bank in 2012 (*http://data.worldbank.org/about/country-classifications/country-and-lending-groups*). Note that many European countries are LMIC.

5. **Study designs included.** All study designs will be included provided they report on an assessment of the outcome of an intervention. These studies may thus be with or without a

control group, (e.g. RCTs, cluster randomised trials, pre-test/post-test studies), process evaluations (provide data on aspects such as design, content, delivery, satisfaction and evaluation of an intervention), or qualitative research (conducted as part of process or outcome evaluation, or to provide women's views of intervention acceptability, appropriateness or the barriers and facilitators of uptake of relevant health care). Only systematic reviews will be included, narrative reviews are classified as "Not research".

6. **Dates of publication included.** Studies published between 2000 and 2012 will be included.

7. **Languages included.** Arabic, English, French, Japanese, Portuguese and Spanish language studies will be included. During screening, if the title or abstract is one of these languages, but you do not know that language (or you are unsure what language it is), and thus cannot decide on eligibility of the title/abstract then please mark it as a query and state the language that it is. If in doubt, mark as a query and always note the reason for your query.

8. **Academic theses included.** PhD theses will be included if located in the search.

Exclusion criteria for Stage 1 of MASCOT/WOTRO review

1. **Study designs excluded.** Exclude descriptive studies, such as those documenting prevalence of conditions and needs assessments. The study has to describe and assess an intervention to be included. Studies merely describing an intervention are excluded, outcome data are required. Policy discussion papers on system or multiple/complex interventions will be excluded unless they provide outcome data. Books are excluded
2. **Single-clinical interventions excluded** Studies of the effectiveness of single clinical interventions will be excluded (apart from the tracer conditions listed above). Thus, excluded are studies of single drug interventions, single surgical interventions, single laboratory procedures or single clinical procedures. Studies comparing a single clinical intervention to another single clinical intervention (or to two other single clinical interventions) are also excluded (e.g. an efficacy trial comparing two drugs, or two surgical procedures). Also exclude articles on use of single tools to monitor individual patients, such as a partogram. However, include tools for monitoring of overall services (such as an audit), as a health system intervention. **Often case studies of an intervention are single clinical interventions.**
3. **Topics excluded.** We exclude interventions related to infertility or fertility (such as contraception failure rates). We only include interventions around contraception if part of a postnatal care intervention, and if they meet other inclusion criteria. Kangaroo care, and similar interventions, in the postpartum are excluded as they aim largely to enhance child health.
4. Academic theses excluded. Exclude masters theses. Books are excluded.

6. General instructions for Stage 1 screening of titles, abstracts and full text

EPPI-Reviewer 4 will be used for screening of titles, abstracts and full text, and for several steps in mapping in Stage 1 and in Stage 2 of the review. This software is developed and maintained by the EPPI-Centre of the Institute of Education at the University of London, UK. To find out more about the

work of the EPPI-Centre visit their website eppi.ioe.ac.uk. There are several useful YouTube tutorials on the EPPI-reviewer software: see the instructional videos at: http://www.youtube.com/user/eppireviewer4. All reviewers should watch these YouTube videos prior to beginning screening.

Please use Firefox internet, rather than Internet explorer or any other option. To get a user name and password, please sign up for a one month free trial, so that we have your email address and user name on the system. First click on http://eppi.ioe.ac.uk/cms/Default.aspx?tabid=2935 and then click on "New account". Also useful is: http://eppi.ioe.ac.uk/cms/Default.aspx?alias=eppi.ioe.ac.uk/cms/er4, followed by clicking on the blue text: **Start using EPPI-Reviewer 4 today by signing up for a free one month trial here!** Once you have a user name, please MF Chersich or J Kavanagh know what it is, so we can link you to the review. You will then receive an electronic invitation to join the review.

The login page for EPPI-Reviewer 4 can be found at: http://eppi.ioe.ac.uk/eppireviewer4/. Enter your user name and password. Click on Go next to MASCOT Demo, then the Collaborate tab (2nd from right in top row of tabs). Locate the articles allocated to you in the list of coding assignments. It is important that you click on the articles allocated to your user name and screen only those articles. Look for your name under the reviewer column (if you click on another person's allocation that work will not be saved). The allocations are named using the first 4 letters of the two reviewers' first names and the date of allocation (mmdd). elin_jose_1008 is the allocation for Elinor and Josephine made on October 8. Then **click on number in the remaining column** to open your allocations **(DO NOT CLICK ON ROWS THAT DO NOT CORRESPOND TO YOUR LOG-ON OR CLICK ON NUMBERS IN ANY OTHER COLUMN THAN REMAINING).** Once your list of articles to screen has opened, click on GO at the top left of the page to open your allocated articles for screening.

If you are unclear on coding, code the study as a QUERY. You must note the reason you are unclear in the notes box which is called "info". Click on "info" to add any notes or queries you have (this applies throughout, note any issues in the info box as you go along, rather than noting issues in emails or other places). If the study meets all the inclusion criteria then INCLUDE it. Note that the definition of each code can be viewed by clicking on the code name and looking at the grey-shaded box at the bottom left of the screen.

Please do not alter the definitions, the codes or coding structure. Rather contact Matthew of Josephine with any suggestions about how to improve the codes or definitions.

A list of several key examples of coding, practical illustrations of the rules below, are included in Annex 5 and 6. Please review these examples prior to beginning coding.

7. Screening of titles and abstracts in Stage 1: variables and instructions

Reviewers will screen document titles and, if required, abstracts, with a low threshold for searching full text. Each article will be coded according to the final coding system for Stage 1 that resulted from piloting of potential codes. Piloting of codes gave a good indication of the volume of evidence available on each topic. Definitions were made for each code that is used in Stage 1. This section and Section 7 lists the codes and instructions for reviewers doing the screening of titles and abstracts.

Screening will be done in independently, in duplicate. Pairs of reviewers will be made to draw on complementary skills, by pairing a clinician with a public health person, for example. Differences in extractions will be reconciled by a third reviewer.

Data variables to extract in screening of titles and abstracts in Stage 1:

1. **EXCLUDE** on title and/or abstract, and why excluded (<u>hierarchy approach: mark only highest applicable item on list</u>):
    - An excluded language
    - Publication pre-2000
    - Population not maternal health
    - No intervention or outcome
    - Single clinical intervention (other than the selected tracer conditions)
    - Not LIMC
    - Not research
    - Other, specify
2. **INCLUDE**, code the topic and study design for all included studies <u>(multiple-response question, MARK ALL APPLICABLE!)</u>
    - **Include Interventional Topic** <u>MARK ALL APPLICABLE RESPONSES</u>
        - Health systems or multiple clinical interventions
        - Community-based interventions
        - Maternal malaria
        - Maternal BP/Hypertension
        - Maternal HIV/STIs
        - Antepartum postpartum haemorrhage
        - Pregnancy sepsis
    - **Include Other**
        - Service utilisation/coverage
3. **NO ABSTRACT**, title indicates article may be relevant but abstract not available
4. **QUERY**, need Full Text to decide if INCLUDE (specify reason for query).
5. **DUPLICATE**

4\. **BACKGROUND** is EXCLUDED or INCLUDED on TI/AB, but need to check references of an article, or is an article of much interest to the review

Once an allocation of references has been opened, click on Screening ti, ab under the header Codes (top left). Then click on the word SCREENING CODES to expand the list of codes. When the categories are expanded, you will be able to see the phrase "Duplicate" at the top of the list.

Each article must be coded within only one of the following four categories: exclude; include, query or duplicate. A few articles will be coded into one of these four categories and also into the category background (defined below).

Instructions for coding on title and abstract

1. If the study does not meet all the inclusion criteria then EXCLUDE it.

2. Each included item should be marked at least twice, except for those coded "No abstract". For included articles, mark both "Include retrieve full paper" and the topic section, and sometimes more than one topic section. For example, a study on a PMTCT system intervention around childbirth that compares an intervention hospital and another control site, should be marked Include retrieve full text, Health System and Maternal HIV/STIs.

3. Definitions of health systems. Single or multiple interventions related to the 6 health system building blocks or interventions to increase patient demand, **OR** the provision of multiple clinical interventions, such as packages of care. Any intervention to raise patients' use of antenatal, childbirth or postpartum services should be included, such as cash transfers, or outreach. Examples of interventions in each of the 6 building block are in the study definitions section (at beginning of protocol). The 6 blocks are: 1.Service delivery: packages; a control program for a single or multiple conditions in pregnancy, delivery models; infrastructure; management; safety, quality. 2. Health workforce: training of health workers, national workforce policies, investment plans; advocacy; norms, standards, data. 3. Information: facility, population based information & surveillance systems; global standards, tools. 4. Medical products, vaccines & technologies: norms, standards, policies; reliable procurement; equitable access; quality. 5. Financing: national health financing policies; tools, data on health expenditures; costing. 6. Leadership & governance: health sector policies; harmonization, alignment; oversight, regulation. 6. Leadership and governance: health sector policies; harmonization and alignment; oversight and regulation. Studies on integration of MH services are considered health system studies.

4. No abstract: Title indicative of a relevant study, But no abstract available. If, based on title alone, you are unable to make a decision on whether the article is not relevant, mark the abstract as a query. If no abstract is available, but title clearly indicates article not relevant then exclude the paper. The review team will then obtain the full text of these articles and assess this for eligibility.

5. Other: note the reason for excluding the study

8. Screening of Full Text articles in Stage 1

Here, we screen the full text of all articles included in the screening of title and abstract. The full text articles are checked to ensure that the codes applied when the titles and abstracts were screened are correct.

Codes for screening of full text articles in Stage 1: CODE SET on EPPI-Reviewer called: Screening of ALL FULL TEXT

Duplicate

Include Health systems, including health promotion

Include Community settings

Include tracer condition/clinical intervention

Include tracer condition/other interventions

Include-Service utilisation and non-intervention (ONLY EXTRACT ARTICLES IN THIS GROUP IF ON THE CLINICAL TRACER CONDITIONS)

Include - query

EXCLUDED CATEGORIES OF ARTICLES <u>(NO FURTHER EXTRACTION TO BE DONE)</u>:

- Exclude - not maternal health
- Exclude language
- Exclude - pre 2000
- Exclude - no intervention/outcome
- Exclude-Non-relevant clinical intervention(s)
- Exclude - not LMIC
- Exclude - not research

Background only (use sparingly) e.g. need to check references of an article, or is an article of much interest to the review

Query unclear (details)

DO NOT MAKE ANY CHANGES TO CODES IN THE SET CALLED SCREENING TI,AB

Click on Go next to MASCOT Demo, then the Collaborate tab (2nd from right in top row of tabs). Locate the articles allocated to you in the list of coding assignments. It is important that you click on the articles allocated to your user name and screen only those articles. Look for your name under the reviewer column (if you click on another person's allocation that work will not be saved). The <u>allocations are named using the first 4 letters of the two reviewers' first names and the date of allocation</u> (mmdd). elin_jose_1008 is the allocation for Elinor and Josephine made on October 8. Then **<u>click on number in the remaining column</u>** to open your allocations **<u>(DO NOT CLICK ON ROWS THAT DO NOT CORRESPOND TO YOUR LOG-ON OR CLICK ON NUMBERS IN ANY OTHER COLUMN THAN REMAINING)</u>.** Once your list of articles to screen has opened, click on GO at the top left of the page to open your allocated articles for screening.

Please do not alter the definitions, the codes or coding structure. Rather contact Matthew of Josephine with any suggestions about how to improve the codes or definitions. A list of several key examples of coding, practical illustrations of the rules below, are included in Annex 6. Please review these examples prior to beginning coding of Full Text. Note that the definition of each code can be viewed by clicking on the code name and looking at the grey-shaded box at the bottom left of the screen.

When you upload a PDF, in the coding group "retrieval of full text", click the box "Retrieved and uploaded to ER4"

Once you have opened your allocation of articles to screen, on the top left, click on the code set "Screening of ALL full text". When the categories are expanded, you will be able to see the phrase "Duplicate" at the top of the list. Also click on the code set "Full text keywords".

Perform Full Text Screening, by checking the article is eligible, and reclassify if required.

Please confirm that the PDF that was uploaded is the same as the abstract, some errors in uploading may occur. Delete the PDF if it is the incorrect one. If the PDF is correct, but there is additional information that you require, let Matthew Chersich know by email (e.g. the PDF of the study protocol may have been uploaded, but not the final report)

Each article must be coded within only one of the following categories: exclude (only one exclude category, the highest applicable category); include (multiple responses are possible, **please tick all include categories that apply**), query or duplicate. A few articles will be coded into one of these four categories and also into the category background (defined below).

Note that some articles that were included on screening of title and abstract will be excluded on review of full text. If the full text article does not meet the inclusion criteria (as defined below) then EXCLUDE it. The exclude category uses a hierarchy approach, whereby the reviewer must mark only the exclusion criteria highest on the list that applies to the study. For excluded articles, mark only one code. Mark the highest option, e.g. if an article describes a study in the USA (not a LIMC) and is in Chinese, then mark "Language" as "Language" is higher on the list than "Not LMIC" **In particular, recode any high-income country papers into this category. Check the list of LMICs (Annex 4) if unsure whether country of study is LMIC.**

If you are unclear, code the study as a "QUERY unclear". You must note the reason you are unclear in the notes box which is called "info". Click on "info" to add any notes or queries you have. If the study meets the inclusion criteria then INCLUDE it as a Clinical Review; Health Systems; Community Intervention; or Include other article. Click all applicable INCLUDE categories. For example, a study on a PMTCT system intervention around childbirth that compares an intervention hospital and another control site, should be marked Include Clinical Review and Include Health Systems.

Notes on included articles at the full text stage

Articles on multiple clinical interventions are only included if they address one of the tracer conditions. Multiple clinical interventions are excluded if they do not address one of the tracer conditions, mark them as "Exclude non-relevant clinical intervention". Reviewers must please note that qualitative articles often report both interventions and outcomes. Interventions to be included in the review may be provided to individuals or groups of women (in childbirth, during or after pregnancy); to staff providing services to these women; to the facilities where these women receive services; or to the community where these women live, including men in these communities. The unit that receives the intervention varies considerably. But please do include the paper provided the intervention relates, in some way, to women in childbirth, during or after pregnancy. This includes involvement of men in maternal health.

Interventions to be included in the review may be provided to individuals or groups of women (in childbirth, during or after pregnancy), or to staff providing services to these women, or to facilities where these women receive services. The unit that receives the intervention may thus vary. But

please do include the paper provided the intervention relates, in some way, to women in childbirth, during or after pregnancy.

Instructions for background articles

Here we aim to flag a few papers which will be especially useful when writing up the background to the reviews, or to the conceptual framework. These articles must also be coded as exclude, include, or query. This might include studies which do not meet all our inclusion criteria. Use this code sparingly. Ensure that all background papers are also classified as exclude, include or query. Please do not aim to be comprehensive with coding such articles. It does not matter if you miss identifying some key background studies.

Instructions for duplicate articles

Some duplicate articles may still be found, please code the first of the duplicate articles as include/exclude or query, and then code the subsequent duplicate article(s) as duplicate.

Full text codes for screening, included studies

1. **Codes for <u>Include intervention study</u>** fall within the categories: health systems, community-based interventions, service utilisation and clinical tracer condition articles). These codes are:

1.1 **<u>Include Health systems, including health promotion</u>**. Single or multiple interventions related to the 6 health system building blocks or interventions to increase patient demand. Any intervention to raise patients' use of antenatal, childbirth or postpartum services should be included, such as cash transfers, or outreach. Examples of interventions in each of the 6 building block are in the study definitions section (at beginning of protocol). The 6 blocks are: 1.Service delivery: packages; a control program for a single or multiple conditions in pregnancy, delivery models; infrastructure; management; safety, quality. 2. Health workforce: training of health workers, national workforce policies, investment plans; advocacy; norms, standards, data. 3. Information: facility, population based information & surveillance systems; global standards, tools. 4. Medical products, vaccines & technologies: norms, standards, policies; reliable procurement; equitable access; quality. 5. Financing: national health financing policies; tools, data on health expenditures; costing. 6. Leadership & governance: health sector policies; harmonization, alignment; oversight, regulation. 6. Leadership and governance: health sector policies; harmonization and alignment; oversight and regulation. Studies on integration of MH services are considered health system studies. Also includes studies reporting outcomes of: organisation of care interventions; or outcomes of national, provincial or district-level MH programmes. Includes studies of socio-economic or environmental interventions, e.g. improving water supply.

Health promotion includes: health promotion activities and health education activities within the community, and for the community, including that which occurs in health service settings. Key topics of interest are: Maternity waiting homes, Health education, Birth and complication preparedness, TBAs in the health services, Role of men/ role of other community influentials, Community participation in development/ delivery/quality/evaluation of intervention/ services/programme, Community

participation in maternal death reviews, Community participation in public accountability, Participatory learning and action cycles, Transport schemes, Demand-side financing schemes, Promotion of human rights, Companion of choice at birth, Respectful care/Cultural competencies/Training of providers in communication and counselling, Community health worker/services in the community.

To be included as an intervention, the study must report an outcome, whereby an intervention is described and linked to findings of an intervention. Interventions in the Health information building block often do not contain an outcome, are merely audits of maternal death or service utilisation. A comparison of two alternative means of assessing maternal mortality would be considered a health information intervention. Similarly, if the study assess whether the information gathered in an audit was able to alter practice that would be considered an intervention, but not merely doing of an audit/assessment.

PMTCT programmes are not necessarily health systems interventions, rather health services. The PMTCT intervention may consist of training, but only if that was a substantial part of the intervention and of the evaluation, then that would be considered a human resource intervention, not if only a minor part of the study.

1.2 Include Community settings. Interventions delivered in community settings (any activities occurring outside health facilities), provided outcome described (including process/uptake outcomes), even delivery of single clinical interventions. INCLUDES community 'micro-financing' & 'peer services'. Single or multiple interventions in field or community settings. Outcomes must be described.

1.3 Include tracer condition/clinical intervention. Single or multiple interventions for the 5 selected tracer conditions. Each is described below.

1.3.1.1 Maternal HIV/STIs include all studies of single or multiple interventions related to HIV or STIs in pregnant, intrapartum or postpartum women. HIV studies include those only addressing PMTCT (i.e. all PMTCT studies to be included here). Outcomes must be described. Bacterial vaginosis was not considered an STI.

1.3.1.2 Maternal malaria include all studies of single or multiple interventions related to malaria in pregnant, intrapartum or postpartum women. Outcomes must be described.

1.3.1.3 Maternal hypertension include all studies of single or multiple interventions related to hypertension in pregnant, intrapartum or postpartum women, such as use of Magnesium Phosphate ($MgSO_4$) for eclampsia. Outcomes must be described. Studies on conditions that are risks for hypertension, such as antiphospholipid syndrome, should not be included unless they have a focus on hypertension.

1.3.1.4 Antepartum or postpartum haemorrhage includes all studies of single or multiple interventions related to haemorrhage in pregnant, intrapartum or postpartum women. This includes studies of drugs such as misoprostil for preventing postpartum haemorrhage, but not use of this drug for inducing labour or for any other purpose.

Outcomes must be described. **Cases of uterine rupture were not considered antepartum haemorrhage.**

1.3.1.5 Pregnancy sepsis includes all studies of single or multiple interventions related to pregnancy sepsis in pregnant, intrapartum or postpartum women. Outcomes must be described.

2. Include - Service utilisation and non-intervention category will be coded at a later date. Studies describing utilisation rate or coverage of services (single or multiple services). DO NOT code studies to enhance utilisation with this code, only descriptions of utilisation. **If you can make an argument that the findings/outcomes reported are related to an intervention, then do not code it as Service Utilisation. Hypothetical interventions, where women are asked about their attitudes to a possible intervention in future are excluded.**

3. Codes for **Include other.** These articles do not necessarily include on intervention, but cover routine services (which should be considered an intervention for this code).

3.1 Descriptions of service utilisation. Quantitative or qualitative descriptions of levels of service utilisation or coverage of services are included, either of single or multiple services. Also include studies describing the characteristics of a population who attended a MH service. Also include studies of costing of utilisation of existing services. If studies report an intervention to alter such use, they must not be coded as a service utilisation study, but as a health systems intervention.

3.2 **Include – query**. Meets all inclusion criteria but not clear which of above topics.

Full text codes for screening, excluded studies

The exclude category uses a hierarchy approach, whereby the reviewer must mark only the exclusion criteria highest on the list that applies to the study. Thus, for excluded articles, mark only one code. Mark the highest option, e.g. if an article describes a study in the USA (not a LIMC) and is in Chinese, then mark “Language” as “Language” is higher on the list than “Not LMIC”

Exclude codes (in the hierarchy order) are:

1.1 Population not Maternal Health: Exclude studies on infertility, fertility (such as studies on population-level effects of fertility rates) or on failure of contraception. Maternal health=women in pregnancy, intrapartum, or 2 years postpartum (studies on abortion are included as maternal health).

1.2 Language not included in our list of languages: Exclude studies published in any language other than Arabic, English, French, Japanese, Portuguese and Spanish.

1.3 Publication pre-2000. This refers to date of publication, not date of the intervention.

1.4 No intervention: Paper doesn't report outcomes of a clinical or system intervention, it describes burden of disease, risk factors or a possible intervention without reporting any intervention outcomes, for example. Basic laboratory interventions unrelated to direct patient care are also not considered interventions in this review. Studies only reporting

findings of routine information or surveillance are excluded, there has to be an intervention to alter the health information system, or its use.

1.5 Not relevant clinical intervention(s). These are excluded single or multiple clinical interventions on conditions other than the tracer conditions of interest. Exclude studies of single clinical interventions, e.g. giving a single drug, a single surgical procedure or use of a single laboratory test. Studies comparing a single clinical intervention to another single clinical intervention (or to more than one other single clinical intervention) are also excluded (e.g. an efficacy trial comparing two drugs, or two surgical procedures). Case studies, which report the outcome of an intervention or more than one intervention on a single clinical case, is considered a single clinical intervention. This means we classify reports of single clinical cases as a single intervention even if more than one intervention was done on the case. Note the case studies of a health system or service intervention must not be classified as a single clinical intervention, only clinical cases must. Case series (reports of more than one case) should not be treated as single case studies, but follow rules for other study designs.

1.6 Not LMIC: Exclude any study which does not take place in a low- or middle-income country. Also exclude studies which take place among low-income groups in upper middle income to high income countries. Consult Annex 4 for full list of LMIC countries. Note that many European countries are included in this list.

1.7 Not research: Paper includes only policy discussion, descriptions of government policies, editorials, or an opinion on a topic. This does not include articles that are systematic reviews, which should be considered research.

1.8 **Query unclear (details):** Please enter your query here, why the coding cannot be applied, or any query you have.

1.9 **WHO background.** Tick this box if article provides important background info for the WHO review. Another group has to be ticked, not only this one.

9. Extraction of data on Full Text articles in Stage 1: mapping of interventions for tracer conditions and health systems

Ensure the article has been coded in the section called Screening Full text

The map developed in Stage 1 will be used to identify potential topics for systematic review, as well as the studies to be included in these reviews. The map will thus identify where there are gaps in systematic review evidence in this field, and the available research data that can be usefully synthesized by the Wotro team and in WP-5 of Mascot. The map will inform discussions with the other stakeholders in which we identify the most policy-relevant review topic(s) to review in Stage 2. Systematic review topics would then be prioritized by the study team, and Wotro and MASCOT partners. Topics identified as high priority, could then be allocated to interested people within the team for leading a stage 2 review.

The map developed in stage 1 will also produce a freely available and searchable resource open to any user. It may be an especially useful resource for other research groups and funding bodies to identify systematic review topics of relevance to reducing health inequalities in maternal health in LMIC. The map will also identify gaps in primary research relevant to reducing health inequalities in maternal health in LMIC. Two articles will be written based on the findings of Stage 1 mapping, these are outlined below.

Full data extraction will then only be done on studies eligible for the specific review in Stage 2. This is then followed by synthesis of results, including an assessment of generalisability of review findings and quality of included studies.

For the Wotro project, decisions will have to be made in Stage 2 as to whether to focus on health service or systems questions in the review.

Variables to be extracted in the mapping elements of Stage 1 were piloted and then finalised. Data extraction will occur in duplicate initially, but if quality is high may occur in single. Extraction is based on the abstract and full text articles. In this stage, articles that have been incorrectly allocated to a category in Stage 1 may be reassigned to their correct category. The full text of articles may, for example, show that a study was actually done in a high-income country. This article is able to provide information on whether the amount of research done in a particular country or region matches the burden of maternal mortality (is research distributed equitably).

The data variables to be extracted for this paper vary depending on which category articles have been assigned in Stage 1. For example, papers classified as HIV/STIs will be coded as interventions: for HIV, for STIs other than HIV, or for HIV and other STIs, a coding system that will not be applied to other categories. The table below depicts the categories of articles of interest for this article and the variables to be extracted for this article.

Studies of multiple clinical interventions will only be extracted if they concern one of the tracer conditions.

Approximate number of articles for full text extraction in stage 1

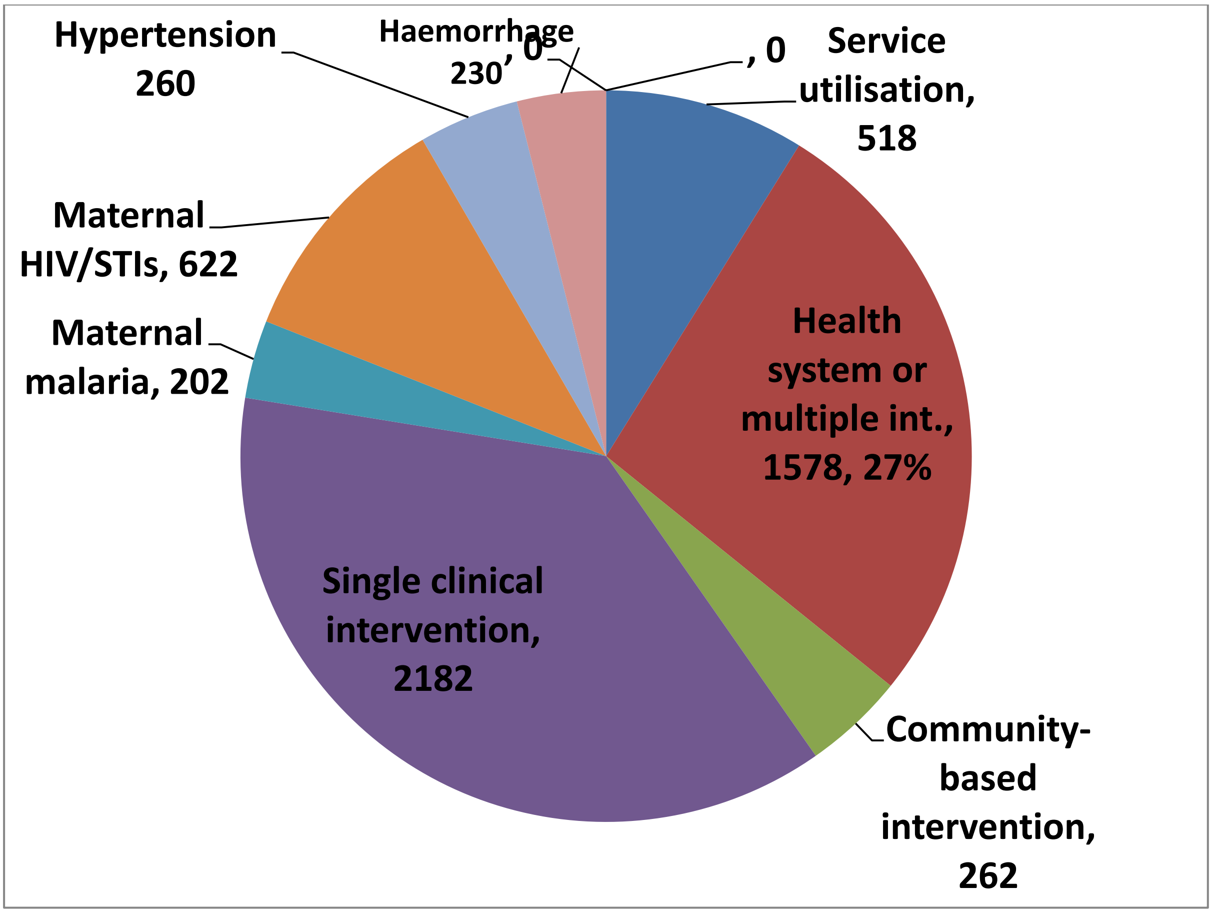
Hypertension
260
Haemorrhage
230
, 0
, 0
Service
utilisation,
518
Maternal
HIV/STIs, 622
Maternal
malaria, 202
Health
system or
multiple int.,
1578, 27%
Single clinical
intervention,
2182
Community-
based
intervention,
262

9.1 Full text extraction for mapping clinical tracer conditions in Paper 1

Data to extract from full text for overall mapping article. CODE SET on EPPI-Reviewer called: FULL text keywords: A. Generic codes/keywords. For HIV studies, also use the code set called: B. <u>HIV tracer condition codes</u> (immediately below generic codes).

<u>If a variable code says "(add details)" then add info to the box, do not just tick the box.</u>

Variables to be extracted from full text of all articles included after screening of full text. Articles coded only as service utilisation and not one of the tracer conditions are to be coded latter

<u>A. Generic codes</u>, apply to all included FULL TEXT articles:

Excluded on Full text

Language not English (add details) Add language to text box if known

Service utilisation non-tracer (2 be coded later). Service utilisation studies covering tracer conditions must be coded now.

1. Country(ies) (tick all that apply) where research conducted. Tick next to name of country(ies) or type name of country(ies) in other details
2. Country(ies) of first author affiliation. Tick next to name of country(ies) or type name of country(ies) in other details
3. Study population is a PROGRESS-Plus group? PROGRESS-PLUS=Place of Residence, Race/Ethnicity, Occupation, Gender, Religion, Education, Socioeconomic Status, and Social Capital, and Plus represents additional categories such as Age, Disability and Sexual Orientation
4. Paper addresses WHO health promotion? *Tick* Yes if fits into the WHO definition of WHO Health Promotion. Note this is a wide definition involving activities within the community, for the community or with the community, including that which occurs in health service settings, or that which reports community/user involvement/empowerment/engagement. Tick unclear if unsure. Please see below for full definition of WHO health promotion.
5. Research question(s) study might answer (tick all applicable) Health systems (CODE C); Community settings*;* WHO Health promotion; Tracer conditions with single clinical intervention*;* Tracer conditions with complex/multiple interventions*;* Health service utilisation/non-intervention research; Other (details)
6. Study design, enter name of study if provided. Also coded as: Systematic Review; Review (other); Randomised controlled trial (RCT); Effectiveness evaluation including process evaluation (not RCT); Qualitative design; Formative non-intervention research; Other (details); Unclear
7. Intervention topic(s) (tick all that apply) *Emergency obstetric care; Prolonged or obstructed labour; Maternal bleeding/haemorrhage; Sepsis/infection; STIs - other than HIV; Malaria; HIV or MTCT; Hypertension/blood pressure; Induced abortion or post-abortion care; Demand side financing; Miscarriage; Male involvement; Transport schemes;; Traditional birth attendants; Maternity waiting homes; Birth and complications preparedness; Female genital mutilation; Family planning (postpartum or post abortion); Other (add details); Not applicable*
8. DIRECT intervention recipient/population (tick all that apply): Women; Family; Male partner; Community; Community health worker; Traditional birth attendant; Midwife/Nurse; Other mid-level provider (add details); Doctor/Obstetrician; Managers; Planner; Policy maker(s) (add details); Other (add details); Not applicable (add details)
9. Period mainly targeted by intervention (tick all that apply) Pregnancy; Childbirth; Post birth
10. Data collected: maternal health outcomes, service utilisation; cost/health economics; child health outcomes; other
11. Funder. Name of funder, or government funder if mentioned

<u>B HIV tracer condition</u> codes:

1. HIV testing uptake (extract the % of women tested)
2. ARV regimen (extract the ARV regimen provided for women)
3. MTCT risk, HIV transmission rate (tick if study provides rate)
4. Women on ART (tick if study provided ART provision data)
5. Percent women retained in long-term care (tick if study reports data on women retained in long-term care)
6. Birth outcomes reported (tick yes if reports % of newborns with birth defects, stillbirth, prematurity, low birth weight)

Full text screening instructions and definitions of variables for clinical intervention studies

<u>Please confirm that the PDF that was uploaded is the same as the abstract, some errors in uploading may occur. Delete the PDF if it is the incorrect one. No need to let us know if it is the incorrect PDF, we will identify these using the search function.</u>

<u>If you exclude an article on Full text, click the box "Excluded on Full text in the code set "Generic codes".</u>

If the box says "add details" that means you must supply additional information details, for that variable. For example, for a study of antiretroviral drugs given to HIV-infected pregnant women tick the "info box" next to: "B2. ARV regimen (add details)" and enter the Antiretroviral regimen used in the box.

If an article does not fit into one of the categories provided, enter the details in info box of the "other" category if there is one for the variable, or leave it blank. Very few should be left blank

For the Clinical Review, reviewers must code up to the end of the code set called "Generic codes/keywords (ALL Studies)". Articles on single clinical interventions must not be coded with the code set called "C. Specialist health systems codes", ignore these and other code sets below that. These code sets apply only to Health System and promotion articles. If an article is a health system intervention on a tracer condition, and thus eligible for both the clinical and health systems' review, code the article up to the end of A. Generic codes section, the health system team will complete the remaining codes. Make sure that you tick the code "Include health systems, including health promotion" and the tracer condition if the article is on a tracer condition and has health system elements. And tick the box "A5. Health systems".

Sometimes the reviewer has to do some brief additional searching for the information required. For example, an author may give the name of their university but not the country of the university, search Google for the country of the university.

Only some data will be extracted from some study designs. Articles that present summary information from other studies (such as systematic reviews), will not be coded in full. For systematic reviews, do not extract A2 country of study. Extract all other data where possible. Note that for A11, extract the funder of the systematic review, not the funder of the studies included in the review. Leave some fields blank if they do not apply to the review.

Definitions of variables to extract

Codes for administrative purposes

1. Excluded on Full text
2. Tick the box "Language not English" if the paper is not in English (we need the language classified so we can assign non-English articles to corresponding people). Add the language to text box if you know what language it is in, and do not code further. These will be distributed to people with the necessary translation skills. If you do understand the non-English language then extract further.
3. Service utilisation non-tracer (2 be coded later) Studies of service utilisation unrelated to one of the tracer conditions will be coded later, not now. Service utilisation studies covering a tracer condition must be coded now.

A. Codes for analysis

1. Country(ies) where study done (tick the correct box(es) or type name of country(ies). Use a capital letter for writing the first letter of the country name (e.g. South Africa is correct, incorrect is south Africa). Do not use acronyms such as USA. Multiple responses are possible. List both high-income country (HIC) names and LMIC country(ies) names if a study was done in both LMIC and HIC. Do not enter names of countries if the article is a systematic review that covers many studies. *Use name of country from Annex 4.* For systematic review type of articles that do not state the name of the country in which the studies were done, check the studies included in the

review, (see references if necessary), **if you can find one article done in a LMIC then include the review and enter "systematic review: in the info box of the "other" category for this variable**. For modelling studies, enter country of data used in the model if it is a country-specific model, if it aims to model across several countries, tick "other" and enter "modelling study across several countries" in the info box next to "other".

2. Country(ies) of first author (type name of country(ies) of affiliation of author). **Multiple responses are possible.** Enter all country names if 1st author has more than one country of affiliation. *Use name of country from Annex 4.* Enter Nepal in the following example: "Save the Children-USA, Himalayan Field Office, GPO Box 2218, Kathmandu, Nepal". If a study group was given as the first author, then the first name listed in the study group was taken as the first author and her/his country(ies) extracted.
3. Study population is a PROGRESS-Plus group? Tick Yes, if study population is one of the PROGRESS-PLUS groups: Place of Residence, Race/Ethnicity, Occupation, Gender, Religion, Education, Socioeconomic Status, and Social Capital, and Plus represents additional categories such as Age, Disability and Sexual Orientation. Tick NO if, the intervention is ***Universal,*** *i.e. is aimed at the whole group population, not on the basis of individual needs/risks). No is thus ticked if the intervention(s) target the general public or a whole population group that has not been identified on the basis of individual risk or needs.* Tick Unclear if uncertain. We use this code to capture if paper addresses health inequalities/SDOH. If a paper has been done in a rural area do not tick "yes" unless there is very clear indication that the study was done in the area to specifically target the population, as opposed to other populations, for example. Being done in a rural area or urban area is insufficient reason to tick yes, there must be other reasons to make one tick yes.
4. **Paper addresses WHO health promotion?** Health promotion includes: activities within the community, for the community or with the community, including that which occurs in health service settings, or that which reports community or user involvement, empowerment or engagement. The main objectives of health promotion are to increase individual, family or community capacity to contribute to improved health or to increase use of maternal and new born health services. Key topics of interest are: health education; birth and complication preparedness; promotion of human rights/reproductive rights: role of men/ role of other community influential; transport schemes; finance schemes; role of TBAs in the health services; maternity waiting homes; community participation in development/ delivery/quality/evaluation of intervention/ services/programme; community participation in maternal death reviews; community participation in public accountability; participatory learning and action cycles; companion of choice at birth; respectful care, and improved interpersonal and cultural competencies of health providers and services. *Tick* ***Yes, No or*** Unclear.
5. Research question(s) study might answer (tick all applicable). The categories are: Health systems; Community settings (*services provided within community settings);* WHO Health promotion (See definition above); Tracer conditions with a single clinical intervention (*a single clinical intervention for one of the tracer conditions, e.g. just drug provision);* Tracer conditions with complex/multiple interventions (*provision of several interventions or a complex/social intervention for one of the tracer conditions);* Health service utilisation/non-intervention research; Other (details)
6. **Study design codes Multiple responses are possible as NB a paper may report more than one study e.g. RCT and Process evaluation.** The review covers all studies designed to evaluate outcomes of an intervention. We do not exclude studies based on their design alone.

a. **If specified**, enter the **name** of the study/intervention programme in the info box next to the variable "Name of study/intervention. Use the **spelling exactly as given** in the report; this is the **only case** in which the requirement for English spelling does not have to be maintained, in all other cases use UK English spelling.
b. Systematic review. A systematic brings together the findings/opinion/conclusions from a range of previous studies in a systematic explicit manner. A systematic review is explicit in its reporting of the search for studies (i.e. reports the search strategy for specified databases) and the criteria for including and excluding studies; it may or may not include a meta-analysis. It may include a range of study designs including qualitative research.
c. Review (other). Use this code for any non-systematic reviews (i.e. those which do not have an explicit search strategy and inclusion/exclusion criteria). Sometimes called a narrative literature review or overview.
d. Randomised controlled trial (RCT) A study in which an intervention is allocated randomly. RCT includes trials of interventions involving individual or group trials (cluster or stepped wedge etc.). Control groups may receive a placebo or other intervention. An RCT study compares different groups i.e. groups receiving different interventions or different intensities/levels of an intervention with each other; and/or with a group which does not receive any intervention at all. IMPORTANLTY, the participants in an RCT are allocated to the different groups in a random manner i.e. the report states 'randomised' and that a random numbers table, a random code or numbered sealed envelopes were used to allocate participants to study groups.
e. Effectiveness evaluation including process evaluation (not RCT) Any method of allocation different from randomisation as above, or the method of allocation is not stated or unclear. A process evaluation examines the acceptability and feasibility of an intervention; studies the ways in which the intervention is delivered; assesses the quality of the procedures performed by the programme staff etc. It is designed to describe what goes on rather than to establish whether it works or not, and may suggest ways in which the programme design and implementation could be improved. Other designs included are controlled (non-random) trials, where the comparison is between two unrelated groups and receipt of the intervention was not randomly assigned. The following methods also fit this category: "We recorded blood pressure in all 1004 pregnant women using the two different blood pressure machines". Includes observational, non-experimental studies where the researcher does not intervene, but describes and analyses people or situations e.g. case study, case series, case-control study, cross-sectional survey, needs assessment, surveys of user perspectives, policy analysis articles, studies on the validity of new diagnostic tests; among other designs.
f. Qualitative design, using techniques such as focus groups, in depth interviews, key informant interviews, ethnography.
g. Formative non-intervention research This includes studies that use modelling methods as the research technique.
h. Other (details), put health economic studies here if they do not provide information on effectiveness of an intervention.
i. Unclear (details) Code as unclear if unsure of design, noting reason for query

7. Intervention topic(s) (tick all that apply) This information should be available in the title and abstract, or aims of study. It is the topics covered by the intervention in the paper. Tick all topics

that apply, not only the main primary focus of the study. Emergency obstetric care; Prolonged or obstructed labour Other terms to look for include: cephalo-pelvic disproportion; malpresentation; malposition; Maternal bleeding/haemorrhage (**this includes studies of uterine rupture and blood transfusions)**; Sepsis/infection; STIs - other than HIV; Malaria; HIV or MTCT; Relevant HIV related maternal health issues, and Mother to Child Transmission; Hypertension/blood pressure; Induced abortion or PAC; Includes studies about post-abortion care PAC; Demand side financing; Miscarriage; Male involvement; Transport schemes; Traditional birth attendants; Maternity waiting homes; Birth and complications preparedness; Female genital mutilation; Family planning (postpartum or post abortion); Other (add details); Not applicable mark if none of the above applies. STIs other than HIV excludes bacterial vaginosis, which is not considered an STI in this review.

8. DIRECT intervention recipient/population (tick all that apply). Actual population that receives the intervention
    a. Women. This includes interventions for fetal health, such as ANC ultrasound
    b. Family
    c. ***Male partner (any intervention that includes the male)***
    d. Community. The community that pregnant/birthing/post-partum women inhabit. Includes neighbourhoods, schools, local businesses, places of worship
    e. Community health worker. Includes village health workers, filed workers, similar cadres
    f. Traditional birth attendant
    g. Midwife/Nurse
    h. Other mid-level provider (add details) Mid-level provider, but not midwife or nurse, e.g. Medical assistant, clinical officer
    i. Doctor/Obstetrician
    j. Managers/Planners/Policy makers. Managers of health services - personnel managers, finance managers, care team managers etc. Policy maker(s) is the person responsible for policy making which impacts on health services, it can be at the level of a single institution (clinic/hospital) or beyond (area/town/region/nation). For health information interventions, tick this category ("health manager/planner/policy maker).
    k. Other (add details)
    l. Not applicable (add details)
9. Period mainly targeted by intervention or utilisation study (tick all the period(s) that apply). This is the period(s) which the intervention mainly was delivered. For service utilisation articles, which assess the use of services in one of the tracer conditions, code the period that utilisation is assessed:
    a. Pregnancy (this includes abortion and miscarriage)
    b. Childbirth
    c. Post birth (postpartum haemorrhage <6 hours after childbirth is not considered post-birth, but childbirth).
10. Data collected: Here tick all boxes that cover an outcome provided in the paper. **Tick Other only if none of the boxes above are ticked.** Maternal health outcomes (this includes maternal biomedical and mental health outcomes); Maternal health outcomes consist of maternal mortality and morbidity measures in the woman only (this does not include outcome of

pregnancy such as stillbirth or low birth weight baby). Use this code for clinical measures of morbidity, including diagnoses of postnatal depression. For the purposes of this review, social support, adherence to medication and measures of mental and emotional well being must also be coded as maternal health outcomes.

Service utilisation: <u>ITNs are considered service delivery if the nets are clearly distributed by the health sector, including the private health sector.</u>

Cost / health economics (Use for studies which report any cost data linked to an outcome, or an economic analyses of the intervention, e.g. cost effectiveness, cost utility studies etc). Merely reporting the cost of an intervention without linking that to effectiveness or outcomes is not included. The latter studies are sometimes called cost-of-illness studies.

Child health outcomes, this includes stillbirths, fetal outcomes and low birth weight, for example. Fetal health outcomes are included as child outcomes.

Other. Code factors such as knowledge and satisfaction as "Other".

11. <u>Funder name</u>, including name of government if mentioned as the funder. This captures the funder of the study, which is not always the same as the funder of the intervention. *To find funder name, search PDF using the terms" "fund", "support", "financ", "acknowle".* Copy text on funders acknowledged. If no funder acknowledged, tick "No funding acknowledgement". Tick no funder acknowledgements if no funder mentioned. Copy the name of funders of the study or of individuals mentioned, e.g. enter National Institutes of Health if the paper says: "Christy R. Goverder was funded by National Institutes of Health". **Extract also the funder of an investigator's salary if that is mentioned.**

B. Codes to extract from HIV articles

Note that articles coded in the title/abstract stage as Maternal HIV/STIs are coded separately as those on HIV; and those on other STI. Tick both categories if the article covers both HIV and other STIs.

1. Extract the percentage of women who had an HIV test in while pregnant, with the 95% confidence interval around that percentage, or the number tested (numerator) and number offered a test (denominator). If testing was done intrapartum or postpartum then extract that information as well, but note in the details box that the testing was done intrapartum or postpartum.
2. Extract the ARV regimen provided to women in the study. Copy all details about the regimen.
3. Tick the box HIV transmission rate if this information is provided in the article. Do not extract the transmission rate, but only whether this information is provided in the paper.
4. Tick the box Women on ART if the paper reports the proportion of women in the study who were given ART.
5. Tick the box "Reports women retained in care" if the study reports the proportion of women retained in care.
6. Tick the box: "Birth outcomes reported" if paper reports anything on the % of children born with birth disorders, birth weight of newborns, stillbirths, gestation at birth etc.

9.2 Stage 1: Data extraction on health systems interventions for maternal health

Instructions for reviewers

Variables to be extracted are defined here. **<u>Please confirm that the PDF that was uploaded is the same as the abstract, some errors in uploading may occur. Delete the PDF if it is the incorrect one.</u>**

Health systems definition. If in doubt, and unsure whether to include a study, ask the question, does the study help answer – what works, for whom, or under what conditions?

Variables to extract on health systems research in maternal health

C. Specialist health systems codes

INCLUDED CATEGORIES TO EXTRACT FROM HEALTH SYSTEM OR HEALTH PROMOTION ARTICLES

Code all articles from Full text coding that were coded as “include Health systems, include health promotion articles” or “include community setting”.

1. **Developer of intervention: National NGO; International NGO; Government (add details)** give the part of government that implemented the intervention**; Research Group; Other (add details).**
2. **Main implementing agency: National NGO; International NGO; Government (add details)** give the part of government that implemented the intervention; **Research Group; Private sector; Other (add details)**
3. **Intervention delivery extent: Entire country; More than one district but not entire country (I**ncludes states); **Single district; More than one facility but not entire district; Single facility (hospital or clinic); Other (add details)** Includes community
4. **Nature of intervention: Broad system intervention beyond MH (**A system-level intervention directly targeting one or more of the six health system building blocks): **A maternal disease/condition-specific intervention** (A maternal disease/condition-specific intervention that is expected to have (large) system-wide effects); **Other (add details)**
5. **The intervention involves (tick all with predominant focus): Changes to health services** (Changes to health services at the organizational level which are not expected to have a system-wide effect (e.g. modification of patient flow within a health facility); **Health system-level changes** (Building blocks other than service delivery); **Change at community level** (Intervention directly involving community); **Changes beyond health system** (Changes beyond health system, e.g. micro-credit schemes); **Other (add details)**
6. **Number of building blocks: Single; Multiple; None**
7. **Type of health service or system intervention: (**Type of health system intervention (derived from Table 3 in Adam et al., 2012); **Model of service delivery (**e.g. Scaling up, Integration, Quality improvements, a. Service package, b. Health service organisation: delivery platforms, integration, (de)centralisation c. Quality assurance, adherence to protocols. d. Demand creation); **Health human resource strategy (**e.g. a. Health worker training, skills b. Skills mix, task shifting c. Employment conditions (salaries, benefits, career path, training incentives) d. Supervision e. Performance review, registration, accreditation); **Information systems (a.** Availability of information systems b. Timeliness, quality of data c. Enforcing reporting requirements d. Use of data for programme improvement); **Pharmaceuticals & medical technologies (**e.g. a. Availability of drugs and technologies b. Pricing of medicines and medical supplies c. Procurement, supply chain management d. Rational prescription and use e. Introducing/scale-up of new technologies); **Financing interventions** e.g. a. Availability of finances for health (budget allocation, fiscal space). b. User fees, insurance mechanisms. c. Provider payment / incentives. d. Service vouchers (overlap with demand creation above); **Sector reforms / Governance** e.g. Decentralisation a. Roles & responsibility, level of decision making. b. Professionalism c. Accountability (incl community participation, consumer/stakeholder involvement); **Other (add details); Not health service/system intervention, specify**

Variables to be extracted from all health system and community-based articles

For systematic reviews or other review, some fields must be left blank, but please do complete the following sections for review articles:

1. **Developer of intervention: National NGO; International NGO; Government (add details) give the part of government that implemented the intervention; Research Group; Other** (add details). This is the group who does the work of designing or developing the intervention.
2. **Main implementing agency: National NGO; International NGO; Government (add details)** give the part of government that implemented the intervention; **Research Group; Private sector; Other** (add details). This is the group who does the work of implementing the intervention.
3. **Intervention delivery extent: Entire country; More than one district but not entire country** (Includes states); **Single district; More than one facility but not entire district; Single facility (hospital or clinic); Other (add details)** Includes community. Code highest level of the study, e.g. a study of 1 facility in each of 5 districts is coded as more than one district but not entire country. This is the extent to which an intervention is implemented, not the area evaluated, e.g. a programme implemented at national level but assessed in a few hospitals should be coded as "entire country".
4. **Nature of intervention: Broad system intervention beyond MH (**A system-level intervention directly targeting one or more of the six health system building blocks): **A maternal disease/condition-specific intervention (**A maternal disease/condition-specific intervention that is expected to have (large) system-wide effects); **Other (add details)**
5. **The intervention involves (tick all with predominant focus): Changes to health services (**Changes to health services at the organizational level which are not expected to have a system-wide effect (e.g. modification of patient flow within a health facility); **Health system-level changes (**Building blocks other than service delivery); **Change at community level (**Intervention directly involving community); **Changes beyond health system (**Changes beyond health system (e.g. micro-credit schemes); **Other (add details)**
6. **Number of building blocks:** Single; Multiple; None
7. **Type of health service or system intervention: (**Type of health system intervention (derived from Table 3 in Adam et al., 2012); **Model of service delivery (**e.g. Scaling up, Integration, Quality improvements, a. Service package, b. Health service organisation: delivery platforms, integration, (de)centralisation c. Quality assurance, adherence to protocols. d.Demand creation); **Health human resource strategy (**e.g. a. Health worker training, skills b. Skills mix, task shifting c. Employment conditions (salaries, benefits, career path, training incentives) d. Supervision e. Performance review, registration, accreditation); **Information systems (a.** Availability of information systems b. Timeliness, quality of data c. Enforcing reporting requirements d. Use of data for programme improvement); **Pharmaceuticals & medical technologies (**e.g. a. Availability of drugs and technologies b. Pricing of medicines and medical supplies c. Procurement, supply chain management d. Rational prescription and use e. Introducing/scale-up of new technologies); **Financing interventions** e.g a.Availability of finances for health (budget allocation, fiscal space). b.User fees, insurance mechanisms. c. Provider payment / incentives. d. Service vouchers (overlap with demand creation above); **Sector reforms / Governance** e.g. Decentralisation a.Roles & responsibility, level of decision making. b.Professionalism c. Accountability (incl community participation,

consumer/stakeholder involvement); **Other (add details); Not health service/system intervention, specify**

This paper satisfies the Wotro subproject 1a objective, and for Mascot it will aim to: describe how equity effects have been measured in MH studies over the review period, and how such measures have changed over time. No additional information will be extracted from articles about multiple clinical interventions, unless they also included a health system intervention. We will use the findings of this data extraction to think about linkages between HS and MH, and to conceptualise these linkages. This coding aims to enable the review team to identify articles with a systemic approach. We will use the variables extracted in the screening of full text stage and the data extraction of full text stage above, as well as 3. Variables to transfer from data already in EPPI-reviewer data fields (i.e. reviewers do not have to extract these data) (Year of publication and Journal name).

9.3 Stage 1: Data extraction on health promotion interventions for maternal health

Reviewer instructions

Code all articles from "Full text coding" that were coded as "include Health systems, include health promotion articles" or "include community setting". **Tick all codes that apply – interventions could fit into a number of codes. Some full text screening and extraction may be done by single reviewers, once they are proficient with the codes.**

Variables to extract on health promotion interventions in maternal health

**D. Specialist health promotion codes (tick all that apply – interventions could fit into a number of codes)**<br>**Maternity waiting homes:** A maternity waiting home is a setting near a health facility where women can stay in the final weeks of pregnancy. Sometimes called maternity waiting village/facility<br><br>**Health education (not including birth preparedness:** Interventions that use health education with pregnant women, their partners/husbands, their families or with other community members to improve key maternal & new born health outcomes, including improved care practices in the home and improved use of maternal and new born health services**.** Health education must be an explicit component of the intervention. Only include counselling interventions (e.g VCT voluntary counselling and testing for HIV) where the authors have an explicit focus on an education related elements (e.g knowledge outcomes, provider training, service uptake, educational resources).<br><br>**Birth and complication preparedness:** Interventions that works with pregnant women, their partners and families focusing on preparations for birth and in case of complications including who will accompany to the facility, how she will get there, saving funds if needed, what materials to bring, blood donor, etc. Often emergency for after birth including for new born can be discussed<br><br>**TBA's in the health services:** Interventions that involve Traditional Birth Attendants (sometimes called community midwives/traditional midwives). We are particularly interested in interventions that find roles for TBAs that do not involve assisting childbirth but give them other roles to integrate them into health services.<br><br>**Role of men/other community influential:** Any interventions with women, men and/or community members to increase positive male, family and community involvement in supporting the women for care during pregnancy, childbirth or after birth, including care for the child after birth. Other 'community influentials' might include mother in laws, father in laws, other relatives, friends, community leaders, religious leaders who influence decisions and social norms for care during pregnancy, for childbirth and after birth<br><br>**Community participation in maternal death reviews:** Use of methodologies and tools such as community epidemiological surveillance, community-based death reviews, maternal and perinatal death audits, verbal autopsies, and other research

on maternal and newborn health issues, where the community is considered a partner not just a source of information i.e. including the involvement of community representatives in gathering, analysing and using the information.

Community involvement other: Use for community involvement in development, delivery, quality, and evaluation of intervention, services or programmes.

Participatory learning and action cycles: Participatory Learning and Action (PLA) is a form of action research. It is a practical, adaptive research strategy that enables diverse groups and individuals to learn, work and act together in a co-operative manner, to focus on issues of joint concern, identify challenges and generate positive responses in a collaborative and democratic manner. Include any study using this approach that works with women, families or communities.

Social accountability: Social accountability can be defined as an approach towards building accountability (of healthcare providers/services/departments) that relies on civic/community/user engagement, i.e., in which it is ordinary citizens and/or civil society organizations who participate directly or indirectly in exacting accountability.

Transport schemes: Interventions that aim to reduce transport barriers women face in accessing skilled care at birth or birth in a facility. These interventions could include a) Interventions to provide non-conventional transport methods E.g. bicycle ambulance, trucks, buses, boats, ox-carts, modified tricycles with platforms, canoes, taxis, three-wheeled motorcycles and trailers. b) Interventions that provide funds to women for transport / of pay for transport for women e.g. vouchers / community emergency funds or c) Interventions organized by the health system to improve transport to for women to facilities and between facilities.

Promotion of human rights: This includes promotion of human rights, sexual rights, reproductive rights, and right to quality health care. Study should explicitly use the language or approach of 'rights'.

Companion of choice at birth: Any intervention focusing on enabling women to have a companion of choice for birth in a facility. These companions can be partners, TBAs, family members or a doula.

Respectful car: Interventions focusing on combating physical abuse; non-consented clinical care; non-confidential care, non-dignified care i.e. verbal abuse; discrimination in services; abandonment and detention in facilities. E.g. Intervention to put in curtains between beds, increase support and supervision of health care workers to improve how they treat women.

Interpersonal/Intercultural Competencies: Include papers about improving providers and services skills to interact with women including interpersonal training, efforts to understand cultural factors that affect use of care, etc.

Community health worker/Services in the community: Interventions delivered in community settings (any activities occurring outside health facilities), provided outcome described (including process/uptake outcomes), even delivery of single clinical interventions. Includes community 'micro-financing' & 'peer services'. Include interventions that use community health workers where they are mandated to deliver services in the community.

Demand side financing: Interventions to reduce financial barriers women face in accessing ANC, childbirth and post-partum, care. I.e. conditional cash transfers/vouchers/ user fee exemptions/loans and subsidies

Other health promotion activity: Falls under the broad definition of WHO health promotion activities - BUT does not address a PICO question or topic in the list above. I.e. whose objectives relate to increasing individual, family or community capacity to contribute to improved health or to increase use of maternal and new born health services.

10.Stage 2: In-depth systematic reviews for Wotro and Mascot projects

<u>Ideas for specific review questions include:</u>

- Examining service delivery articles, to classify the deficiencies in delivery into the health system building blocks.
- For data extracted on HIV, we will compare the MTCT regimens used with the MTCT rates from UNAIDS reports. We will assess if the Impact factor of journal is associated with having

a RCT design versus other designs, and if RCT design is associated with certain tracer conditions and country of author.

- Review for equity effects of PMTCT.
- Detailed mapping of human resources interventions for maternal health.

The variables to be extract are classified as: characteristics of the study population and setting; and characteristics of the intervention. The conceptual framework for this paper is depicted in Figure 2 below and for measures in Figure 3. Study context is important as the implications of social strata depend on context. Thus detailed information is needed to understand and explore the mediating effect of context. In particular, characteristics of PROGRESS-Plus will be extracted, which is often included only in the baseline description of the study population.

To code in more detail articles coded as Include Community setting, which are articles about interventions done in the community. Intervention recipient (e.g. type of facility) is important. Recipient level systemic, facility based, MH programme level, health system level. Organisational level (clinic/hospital), programme (MH broadly); instrumental level (info and research), system (not confined to programme). Julio Franck tools. Interventions purely initiated by MH workers, protocols adherence, not confined to MH programme. Is this an intervention in a facility, it an intervention initiated in programme, through multiple programmes, or something in the programme or system more broadly. Steps remaining in Stage 1 of the review:

a. Contextual information relevant for the category/categories of disadvantage under consideration in the study
b. Inclusion/exclusion criteria of the study
c. Extract full text sections within documents that explain the mediating effect of context on programme outcomes
d. Did the study describe the socio-demographic characteristics of withdrawals and dropouts, if so what were they?
e. Intervention elements coded as: Education / training; Incentives; Policy documents, guidelines; Equipment/technology provision/access; Social support including Social determinants of health; Strengthening service delivery; Infrastructure development; Other, specify
f. Specific intervention topic if any e.g.: postpartum/postabortion contraception. Or copy free text describing the intervention?
g. Intervention duration, intensity, and mode of delivery, this includes the following intervention components likely to change outcomes of MH services or programmes:

    i. goals and activities of intervention

    ii. human resources involved

    iii. programme implementation

    iv. community activities and involvement

The steps in Stage 2 reviews are:

1. Define the specific PICO question of each review (3 days)

2. Overall coordination of Stage 2 activities (10 days)

3. Define the eligibility criteria for inclusion of articles to address the PICO question and prepare a PICO review protocol, get GRC approval for each protocol (2 days for each PICO)

4. Locate all articles on the topic from the map completed in Stage 1 of the review, checked against eligibility criteria defined for the PICO questions (1 day for each PICO)

5. For each of the studies located, define variables to extract for each PICO, extract additional data on the study intervention, outcomes and research quality (5 days per PICO question)

6. Analyse the data extracted and prepare GRADE tables (5 days per PICO question)

This stage of the review will draw on the revised PRISMA guidelines for equity-based reviews (http://bit.ly/XiqhLg). This second stage begins with defining specific review question(s) presented in the PICOT format, and the conceptual framework(s) for such questions. Once these decisions are made, reviews will be carried out, following pre-specified review methods described in a review protocol for each of the review questions. The person responsible for the review will finalise a review protocol for each stage 2 review. Where possible, we will register each of these reviews and obtain a review registration number. Variables to be extracted in each sub-study in Stage 2 will be piloted and finalised. There are several options in Stage 2 for data extraction. These include a more detailed extraction of a subset of papers, extracting information from both the abstract and full text. Alternatively, we may first do a PROGRESS-Plus screen on some random proportion of the studies identified in Stage 1 to ascertain how many include these groups. It is also possible to randomly select a sub-sample of papers for more detailed assessment.

In this stage we will only include studies that provide quantitative or qualitative evidence of the differential effects of MH interventions for advantaged and disadvantaged groups, who are disadvantaged either by Place of Residence, Race/Ethnicity, Occupation, Religion, Education, Socioeconomic Status, Social Capital, Age, or Disability. The final review questions for this stage will be finalised based on the outcomes of Stage 1. Likely the review will concern the distribution of outcomes of maternal health initiatives or programmes, and less about the distribution of the outcomes of a set of services. Reviews will not cover individual clinical or laboratory interventions.

Original studies on MH interventions will be included, as well as systematic reviews that focus on equity in MH. Excluded are studies which do not report effects of intervention on at least one PROGRESS-Plus group. Findings about differential effects of an intervention may often be sub-group analyses of the study and thus not reported in the abstract. Negative findings of sub-group analyses might especially not be reported in the abstract, making full text searching especially important. Data will be extracted into standard data extraction forms.

Studies within the review will be classified into two categories, or studies with a combination of these categories. Firstly, targeted intervention studies, where the programme/service aims explicitly to target a disadvantaged group or setting (often one of the PROGRESS-Plus groups). The population sample in these studies is thus restricted to disadvantaged populations or settings in which most people are disadvantaged. In targeted studies there is often no comparison group, making it difficult to assess differential effects of interventions on study groups. Some of these studies among vulnerable populations do, however, report outcomes among sub-groups at especially high

vulnerability. The second category, the focus of this review, involves a universal or general population intervention, which was designed for the general population, but may report on the differential effects of the programme/service on different population groups. Data may thus be stratified by one or more categories of differentiation (PROGRESS-Plus categories). These studies may also use proxy measures for disadvantaged strata. Some combinations of targeted and universal study types are also possible. For example, a targeted study of an intervention aimed at poor groups might report effects on the extremely poor, or on those with both poverty and disability, or other combinations of disadvantages.

Qualitative study outcomes that could be assessed

Assess patient experiences of accessing health care services, if these varied across population groups.

Extract qualitative outcomes of descriptions of distribution of outcomes, how an intervention changed this distribution, or how experiences of women of MH services varied according to the PROGRESS-Plus groups, extract full text on these (ask for advice on how to extract qualitative data).

Did the study outcomes report a process evaluation, and how the intervention's quality interacted with disadvantage? If yes, extract that information.

Unanticipated negative and positive effects of the programme/service

Description of trajectory of the impact on outcomes (causal pathway), whether this was a simple bivariate relationship, or non-linear phase changes (tipping point), feedback loops, or due to interactions between components

Further detailed review questions and analysis

Variables to be extracted in Stage 2 will be piloted and finalised in that stage of the review. There are several options in Stage 2 for data extraction. These will be explored in detail once Stage 1 is complete, or near complete. These include a more detailed extraction of a subset of papers, extracting first information from the abstract, or using both the abstract and full text.

1.1 **Equity aspects of interventions:**

1.1.1 Is there any evidence that the interventions were specifically aimed at the disadvantaged, defined across PROGRESS-Plus categories?

1.1.2 how intervention varied systematically across sites or areas

1.2 Types of outcomes and their effectiveness. Immediate, intermediate and long-term outcomes (extract actual outcomes which are disaggregated by the PROGRESS-Plus groups, but also extract the overall outcome in the whole population). Extract adjusted outcomes if both unadjusted and adjusted outcomes are provided:

1.2.1 Outcomes for women: Mortality; Morbidity; Psychosocial; Satisfaction; Knowledge; Utilisation; Wellbeing of women (*includes: quality of life, social cohesion and community integration);* Other, specify

1.2.2 Health worker outcomes; Knowledge/training; Quality of care; Time spent; Satisfaction / motivation; Retention; Other, specify

1.2.3 Outcomes for community: Knowledge; Engagement; Resources; Support for maternal health; Other

1.2.4 Outcomes in PROGRESS-PLUS group: Place of residence; *rural, urban for example*; Race/ethnicity; Occupation; Religion; Education; Socioeconomic status; Social capital; Age (e.g. *adolescent pregnancy for example);* Disability Proxy measures of social differentiation can be used for socio-economic status.

1.2.5 Outcomes for health system: Health information systems; Cost; Recruitment / retention; Service quality; Provision of comprehensive obstetric services; Provision of basic obstetric care; Facility development; Facility improvement; Other

1.2.6 Process outcomes: Skilled attendance; Uptake/coverage (add more in pilot phase, acceptability/feasibility); Other. This considers how the programme was delivered, mechanisms of effects, for whom it worked, in what respects, and under what circumstances. What population actually received the intervention, did coverage vary between PROGRESS-Plus groups.

- Examining service delivery articles, to classify the deficiencies in delivery into the health system building blocks.
- Detailed mapping of human resources interventions for maternal health.

Figure 2: Systematic review exposures (WHO health system building blocks, demand-side initiatives) and outcomes (adapted from Blaauw et al(5))

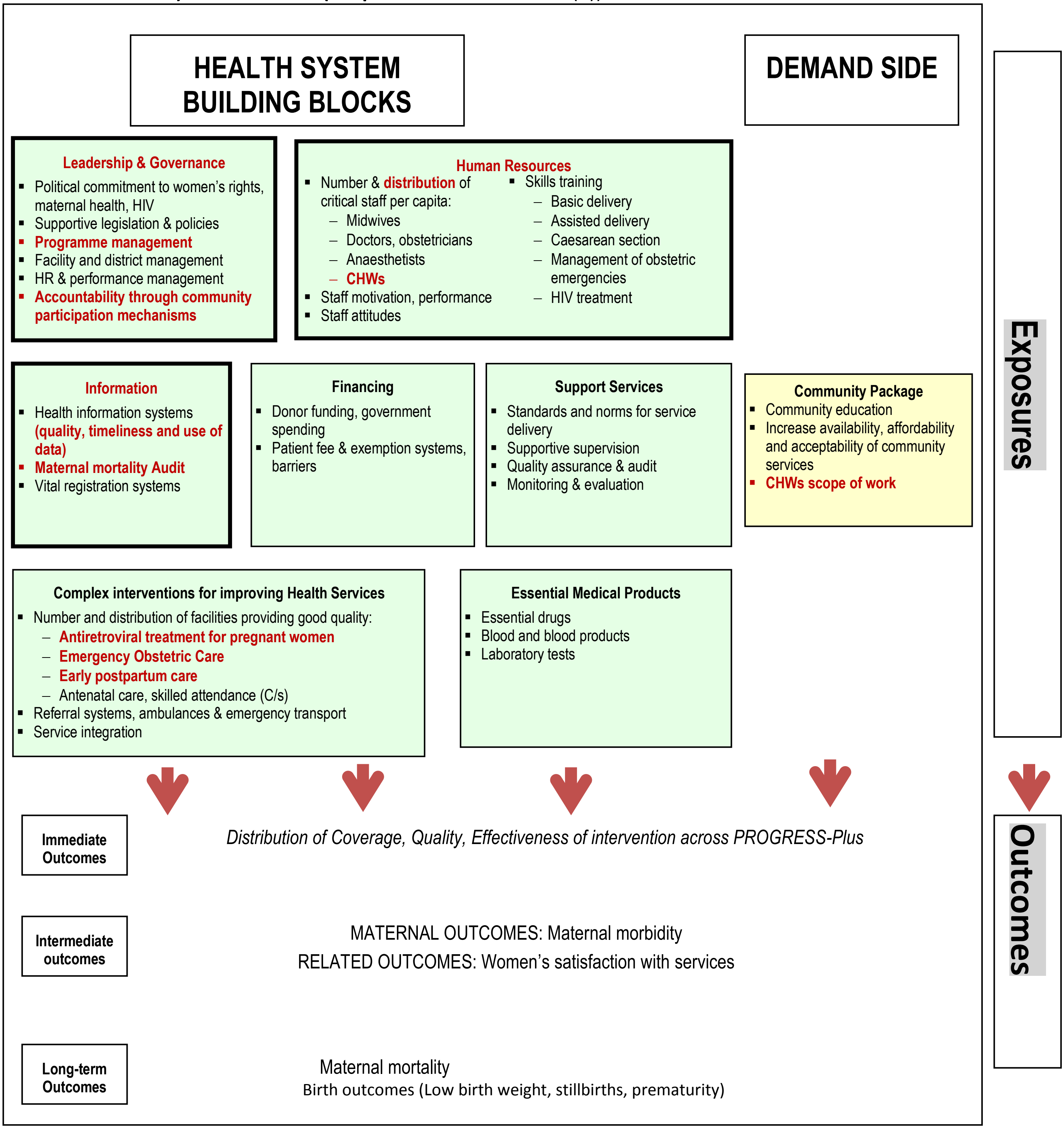

For the WOTRO project, focus areas and health system probes are highlighted in red. These also inform selection of the tracer conditions

Approach to data analysis in stage 2 of Mascot

In evidence synthesis, we will interpret the cumulative evidence in the review to draw conclusions about the relevance of results to the review question. This aims to present evidence on outcomes in disadvantaged populations compared to advantaged ones, and on which interventions optimise such outcomes. We will conduct subgroup analyses across categories of disadvantage (e.g. socioeconomic status, sex, race, etc.) where appropriate. This aims to determine whether equity implications of maternal health programmes differ between categories of disadvantage. Analysis of outcome data related to subgroups (disadvantaged populations) can be done using ***within-study subgroup analysis*** reported by different studies. The alternative is to classify study populations according to their level of disadvantage (e.g. socio-economic disadvantage) and conduct ***between-study subgroup analyses***. Selection of option may be determined by the availability of sufficient data to analyse within-study subgroup differences or between study differences.

The quality of included studies will be appraised. For any trials we identify, quality will be assessed using the GRADE study limitations criteria, and the NICE criteria used to assess quality of evidence for observational studies.

The marked heterogeneity of study exposures and outcomes means results will likely be summed descriptively or qualitatively, rather than in meta-analysis. Where possible, we will analyse both differences in absolute and in relative effects in the categories of disadvantage, and assess the implications of these differences (Carling, 2009).

As this is an equity-oriented systematic review, we will analyse data on gaps, gradients, and targeted interventions based on the fitness for purpose of the summary measure and availability of data (Evans, 2001 presents thorough discussion of gap and gradient analysis). As mentioned, the data will likely be unsuitable for meta-analysis, and summary measures will likely not be used. Data will be summed qualitatively, and presented in tables, using whatever gap and gradient data are available. The harvest plot can be used to analyse the presence of gradients in effect size from complex and diverse studies (Ogilvie, 2008). Compare outcome in sub-group with those not in the sub-group, or with whole population.

We could also conduct a process evaluation in some reviews (with extraction of process data), using qualitative methods to assess why, how, when, and under what circumstances an intervention is most likely to be effective, in different groups. Further, the review will consider what contextual factors enhanced or limited these differential effects of the intervention.

The synthesis will include discussion of the applicability, transferability, and external validity of findings according to accepted criteria, as well as consider context (such as using theory and judgment). Thorough attention to understanding context and process evaluation will aid judgments about applicability. Also, the inclusion/exclusion criteria of the primary studies in each stage 2 review will influence the generalizability of findings. A judgement is required about how much cultural or political context has shaped the original studies' interpretation, and if interventions that are effective in this setting will work in different contexts. Applicability relates to the context in which the primary data were collected and the setting to which they will be extrapolated.

We aim for transparent reporting of judgements made. The risk of bias will be assessed for included studies, according to the potential for bias in selection and detection of primary studies. Finally,

overall conclusions will be drawn about the equity implications of maternal health programmes, and what strategies best reduce inequities.

11.Review limitations and strengths

Though the MASCOT and Wotro teams are spread across more than a dozen countries and three continents, we by no means have global coverage. For examples, in the Mascot review some people will know key grey literature documents in their countries, but not in others. The MASCOT team includes European, Latin American and African countries, and an partner with much experience with Asian research has been introduced for this component of the study (Centre for International Health, Burnet Institute, Australia). Having teams from each region aims to limit the potential for such selection bias and to specifically ensure Asian publications are included.

Many studies will not report the effects of an intervention on specific population strata (ref Welch Cochrane review). This may occur for the following reasons: differential reporting as effects were not measured in population groups; sub-group analysis was not done; or sub-group analysis was done, but not fully reported. Publication bias is likely as negative or null findings of sub-group analyses are less likely to be reported than positive findings (ref Eggar book). We will do formal tests for publication bias, including using funnel plots.

The review aims to include studies of the effects of socio-economic or environmental interventions, such as improving water, on maternal health. We are, however, mostly searching biomedical sources, which may not index all studies on this topic.

12.Review team roles and responsibilities

This section may be updated as additional people join the review. Here we outline the roles of each partner in the review and how authorship and other outputs will be attributed.

Review timelines and milestones

Stage 1 Timelines

**Phase**	**1. Review piloting**	**2. Finalise Stage 1 review protocol**	**3. Identify eligible literature**	**4. Screen articles in Stage 1**	**5. Clean data and reconcile discordant coding**	**6. Prepare map of included literature**
**Indicative timing**	March-April 2012	July-September 2012	July-September 2012	October 2012-Feb. 2013	Dec. 2012- April 2013	April 2013- July 2013
**Outputs**	Present review outline at MASCOT meeting March 2012. Search strategy piloting and decisions made about which databases to search in Stage 1. In total, 45,959 items were	Pilot and finalise Stage 1 methods and protocol. Define CF. Design data capture forms for Stage 1 on EPPI Centre website	Perform searches of selected databases. Upload references into EPPI-reviewer. Remove duplicate articles.	In duplicate, screen articles for eligibility. Present first findings at Oct. 2012 MASCOT meeting in South Africa	Reconcile differences in screening of title and abstract. Resolve queries. Upload full text articles of included articles.	Do full text screening and code included articles on limited mapping variables. Prepare paper on stage 1 mapping findings. Prepare for Stage 2 detailed data extractions.

	added to the online systematic review software EPPI-Reviewer 4. The software and individual reviewers then removed duplicate items totalling 12,071. Independently, in duplicate, we then screened the remaining records (33,888) for relevance on their title and abstract. This screening applied the review inclusion and exclusion criteria. The two reviewers or a third reviewer then reconciled any discrepancies in this coding. From the 33,888 articles reviewed on title and abstract, 4472 were marked for					

	full text review. This is an inclusion rate of 13.2% after screening of title and abstract. We were unable to locate the full text document for a total of 300 articles (6.7%; 300/4472). Of the 3140 full text articles reviewed, a further 45.3% were excluded (1889). In total, 31.305 articles were excluded after screening of title and abstract and after full text review. This is 92.3% of all the articles identified in the review. Of the studies excluded from the review that were on maternal health, the most important reason for exclusion was that the					

	study did not describe an intervention or outcome (33.0%; 10,347/31,305). Other studies that were on maternal health, but were excluded were those on single clinical interventions other than the tracer conditions (13.9%; 4343/31,305) or only provided data on utilisation of routine services (2.0%; 622/31,305). Other reasons for exclusion were: articles published before the year 2000 (20.3%; 6364); studies not on maternal health (25.2%; 7877/31,305); studies not done in LMICs (2.1%; 666/31,305); Not research (3.9%; 1213/31,305)					

	; and an excluded language (1.0%; 303/31,305). In total, we identified 80 articles on community-based interventions that were assessed in an RCT or a systematic review. data fields for Stage 1 extraction.					

Stage 2 Timelines

Phase	1. Choice of in-depth review topics.	2. Finalise Stage 2 review protocol	3. Identify eligible literature
**Duration**	**Month 1-2**	**Month 3-4**	**Month 5-6**
**Outputs**	Select topic for stage 2 article. Define review question. Draft codes for data extraction. Adapt CF for individual review. Pilot codes and finalise codes, protocol, review lead and review team.	Upload PDFs into EPPI website. Extract data in duplicate using EPPI centre tools. Reconcile discordant data extraction. Prepare outline of review paper.	Analyse data. Develop review articles.<br>Submission of peer-reviewed journal articles

Dates not provided for Stage 2 as the process is repeated for several reviews, beginning at different time points.

Stage 1 Mapping specific tasks

Overall task	Person/team leading this work	No. of articles to do	Estimated time to completion
**Review articles coded as "query", "no abstract"**	Siphiwe, Matthew, Langa	900	14 June
**Reconcile differences between screeners of title and abstract**	Ashar, Caroline van de ven, Elinor, Emily, Loveday, Josephine, Matthew, Langa	30,000 reconciled	14 June
**Download PDFs of included articles, upload onto EPPI-reviewer**	Langa, Siphiwe<br>Others depending on Journal article access	2192 PDFs uploaded	14 June
**Assign articles for extraction, train team, perform quality assurance (initial duplicate screening)**	Josephine (assign articles for health system review). Siphiwe (assign articles for clinical review, quality assurance, reconcile discordant reviewers and feedback). Matthew (quality assurance, reconcile discordant reviewers and feedback)	-	-
**Data extraction for clinical tracer condition group**	Malaria (Phyllis, Patricia, Godfrey, Adiel, Shakira); HIV and STIs (Priya & Charles, Carolina Fonseca, Marcela, Francisco, Simukai, Langa); Hypertension (Emily, Mario, Martha), Haemorrhage (Hassen, Imed, Victor, Jihen Maatoug)<br>USE CODE SET: Screening of ALL full text AND A. Generic codes/keywords (under header Full text keywords). Only if includes an HIV intervention use code set B. HIV tracer condition codes. Reconcile discordant screening with your screening partner.	Malaria 320<br>HIV 1000<br>BP 350<br>Haemorrhage 320	15 July
**Data extraction for health**	Leon, Felix, Duane, Matthew, Loveday, Ashar	1500	15 July

**system and community group**	USE CODE SET: Screening of ALL full text; A. Generic codes/keywords (under header Full text keywords); C. Specialist Health System codes; AND Only if includes an HIV intervention use code set B. HIV tracer condition codes		
**Data extraction for health promotion group**	Mari, Loveday, Annie, Pieter<br>USE CODE SET: Screening of ALL full text; A. Generic codes/keywords (under header Full text keywords); C. Specialist Health System codes; AND D. Specialist Health promotion codes. Only if includes an HIV intervention use code set B. HIV tracer condition codes	1250 (uncertain estimate)	30 June

The review team is multi-disciplinary, bringing diverse skills sets and languages. The names of all persons involved in the review and their roles in the review are detailed here. Authorship will be contingent on proportion of the work done, this includes screening in Stage 1, extraction from included articles, analysis and writing up of articles. To be a named author, the following criteria apply:

1. To be included as an author, a person must have completed at least an overall 5% of the screening or data extraction from the included articles. This could be made up of 10% screening and 0% extraction, or 7.5% of screening and 2.5% of extraction, for example. A person who does not have the skills required for data extraction, should focus on ensuring they have done higher levels of screening to cover their contribution. A partner can combine efforts if skill sets or time constraints do not allow one person to complete both screening and extraction. For example, a junior staff member could screen 5% and a senior one extract 5%, the partner will qualify for 1 co-author, and could include alternate between the 2 people who contributed from each partner on each paper.

2. In some instances, senior staff who provide overall oversight, may be included as a senior author or as a named author on a paper, even if they have done less than 5% of the work.

3. As per international guidelines (Uniform Requirements for Manuscripts Submitted to Biomedical Journals: Writing and Editing for Biomedical Publication: http://www.icmje.org/urm_full.pdf), all authors are expected to make a contribution to drafting the article or revising it critically for important intellectual content. This means co-authors should help write the paper, or at least review versions as they progress.

4. Though we will attempt to publish in journals that do not have a limit on author number, some journals do limit the number of authors one can include. The term Mascot Study Group and Wotro Study Group may be used (a group author). The article and the journal will list the names of individuals within this group, who were identified as being directly responsible for the manuscript (fulfilled criteria 1 or 2, and also criteria 3). The National Library of Medicine (Pubmed) indexes the group name and the names of individuals the group has identified as being directly responsible for the manuscript.

5. Authors will be listed in order of the percentage contribution they made to screening and extraction of information. However, weight will also be given to the contribution of analysis and writing up of the article, and the person leading the latter two processes for each article will generally be named as first author of that paper.

6. Other members of Mascot and Wotro who made some contribution to the study, but did not fulfill criteria 1 or 2, and also do not meet criteria 3 will be named in the acknowledgements section of the paper.

The most recent review team is listed in the table below. Other Mascot groups from the Latin American and African partners in the project will join the study team. From CHP, Siphiwe Thwala and Loveday Penn-Kekana and Duane Blaauw are employed within the Wotro project, and others from the University of the Western Cape involved in Wotro will contribute. The table below notes which people will extract which foreign language.

External advisors will be consulted, aiming to obtain the input of a multi-disciplinary team (MH service users, researchers and policy makers) and frequent engagement of this group. The WHO team conducting health system reviews for maternal health and the MCH Initiative will be contacted, and requested to provide advice.

Roles of team members in Stage 2 are presently being developed.

Table: Roles of members of the review team in Stage 1

Name of people doing screening in Stage 1 (M=Mascot W=Wotro)	Institution, email	Planned role of contributor
Ashar Dhana (M &W)	Wits Uni. ashdha@hotmail.co.za	Screening, reconciling discordant screening, and summary of review findings
Carolina Fonseca Cortes (M)	carolinafonsecacortes@gmail.com	Screening
Caroline van de Ven (W)	Radboud, ccvandeven@gmail.com	Screening, resolve discordant screening.
Charles Chasela (M&W)	charles.chasela@wits.ac.za	Screening, data extraction
Christina Zarowsky (W)	czarowsky@gmail.com	Screening
David Sanders (W)	UWC, sandersdav5845@gmail.com	Input on protocol
Debra Jackson (W)	UWC, debrajackson@mweb.co.za	Input on protocol
Duane Blaauw (W)	CHP, Duane.Blaauw@wits.ac.za	Oversight, screening, coordination of health system review codes
Elinor Kern (M&W)	CHP, elinor_kern@yahoo.com	EPPI-reviewer resource person, can solve queries, screening, makes allocations of articles for screening, reconciling discordant screening
Emily Vargas (M)		Screening, reconciling discordant screening, and data extraction
Francisco Becerra (M)	COHRED, becerra@cohred.org	Screening and data extraction
Hassen Ghannem (M)	hassen_ghannem@yahoo.fr	Screening and data extraction
Imed Harrabi (M)	imed_harrabi@yahoo.fr	Screening and data extraction
Janneke van de Wijgert (W)	AIGHD, j.vandewijgert@aighd.org	Input on codes for stage 1 & 2, analysis
Josephine Kavanagh (M&W)	j.kavanagh@ioe.ac.uk	Literature search, upload of articles, removal of duplicates, makes allocations of articles for screening, oversight, limited screening, addressing discordancies between coding, design of codes
Leon Bijlmakers (W)	L.Bijlmakers@elg.umcn.nl	Screening and data extraction, French screening, design of codes, data extraction
Loveday Penn-Kekana (W)	CHP, Loveday.Penn-Kekana@lshtm.ac.uk	Screening reconciling discordant screening, and data extraction.
Marcela Cortes (M)	pmarcelacortes@gmail.com	Screening and data extraction

Martha Perry (M)	HAPI, Martha.Perry@hapi.org.uk	Screening, data extraction
Mario Tristan (M)	IHCAI, mtristan@ihcai.org	Screening, oversight, technical input
Matthew Chersich (M&W)	CHP, matthew.chersich@wits.ac.za	Oversight of review, assist resolving queries, design of codes, training of team, screening, resolve discordant screening
Priya Mannava (M)	Burnet Institute, priyam@burnet.edu.au	Screening and input on coding, draft concept sheet for first paper
Siphiwe Thwala (M&W)	CHP, lathwalas@yahoo.co.uk	Screen TI/AB and resolve queries on full text, data extraction
Stanley Luchters (M)	Burnet, sluchters@burnet.edu.au	Limited screening, input on codes and protocol
Sunisha Neupane (W)	UWC, sunisha.neupane@gmail.com	Screening and data extraction
Thubelihle Mathole (W)	UWC, tmathole@uwc.ac.za	Screening and data extraction
Victor Montekio	victor.becerril@insp.mx	Screening and data extraction

Table: Reviewers to extract non-English papers (to be completed later)

**Name of extractor(s) in Stage 2**	**Language**
Martha Perry, Mario Tristan	Spanish
	Japanese
Martha Perry, Leon Bijlmakers (W)	French
	Portuguese

Table: Roles of members of the review team in Stage 2 (to be completed later)

**Name of person**	**Institution**	**Planned role of contributor**
Elinor Kern	CHP	Resource person
Duane Blaauw	CHP	Oversight, input on design of review questions and extraction tools, , data extraction
Josephine Kavanagh		Oversight, design of EPPI-reviewer tools, data extraction tools and full text screening, resolve queries about EPPI-reviewer software.
Loveday Penn-Kekana	CHP	Design of data extraction tools, data extraction
Mario Tristan		Oversight, technical input, data extraction
Matthew Chersich	CHP	Oversight of review, assist resolving queries, design of extraction codes, data extraction
Priya Mannava	Burnet Institute	
Siphiwe Thwala	CHP	Data extraction
Leon Bijlmakers (W)	L.Bijlmakers@elg.umcn.nl	Data extraction, design of codes, French extraction

Annexes

Annex 1: Search strategies and interim results of literature searches in stage 1

Interim results

As reported in section 4.1 above approaches to searching have been employed that aim to increase the identification of research from low- and middle-income countries. The results of these searches are presented in the table below.

To date 37500 items have been added to online systematic review software EPPI-Reviewer 4. Approximately 4200 records have so far been screened for relevance on their title and abstract. Screening has so far been undertaken by two reviewers working independently then meeting to agree any discrepancies. Agreement has been reached on 1280 research records, of the 800 records of research published since 2000, approximately 40 have been judged to be relevant. This is a low inclusion rate of 0.05%. However it is expected to increase once all duplicates have been removed and the results of searches of African Index MEDICUS, Index Medicus for South-East Asia Region, and African Journals online are incorporated.

Source	Number of hits	Duplicates	Date of search
**CINAHL**	2398	489	Sep 3rd 2012
**EMBASE**	3618	376	21/09/2012
**Popline**	12186	2678	
**PsycINFO**	1139	1	21/09/2012
**PubMED**	13634	2293	17/08/2012
**Web of Knowledge**	8903	3288	21/09/2012
**LILACS**	3450	In process	21/09/2012

When screening of all records has been completed, and in keeping with recommended methods for reporting systematic reviews (Moher et al 2009) we will complete the following flow chart highlighting the flow of studies through the review process .

Figure: Flow chart of identification and selection of studies in Stage 1

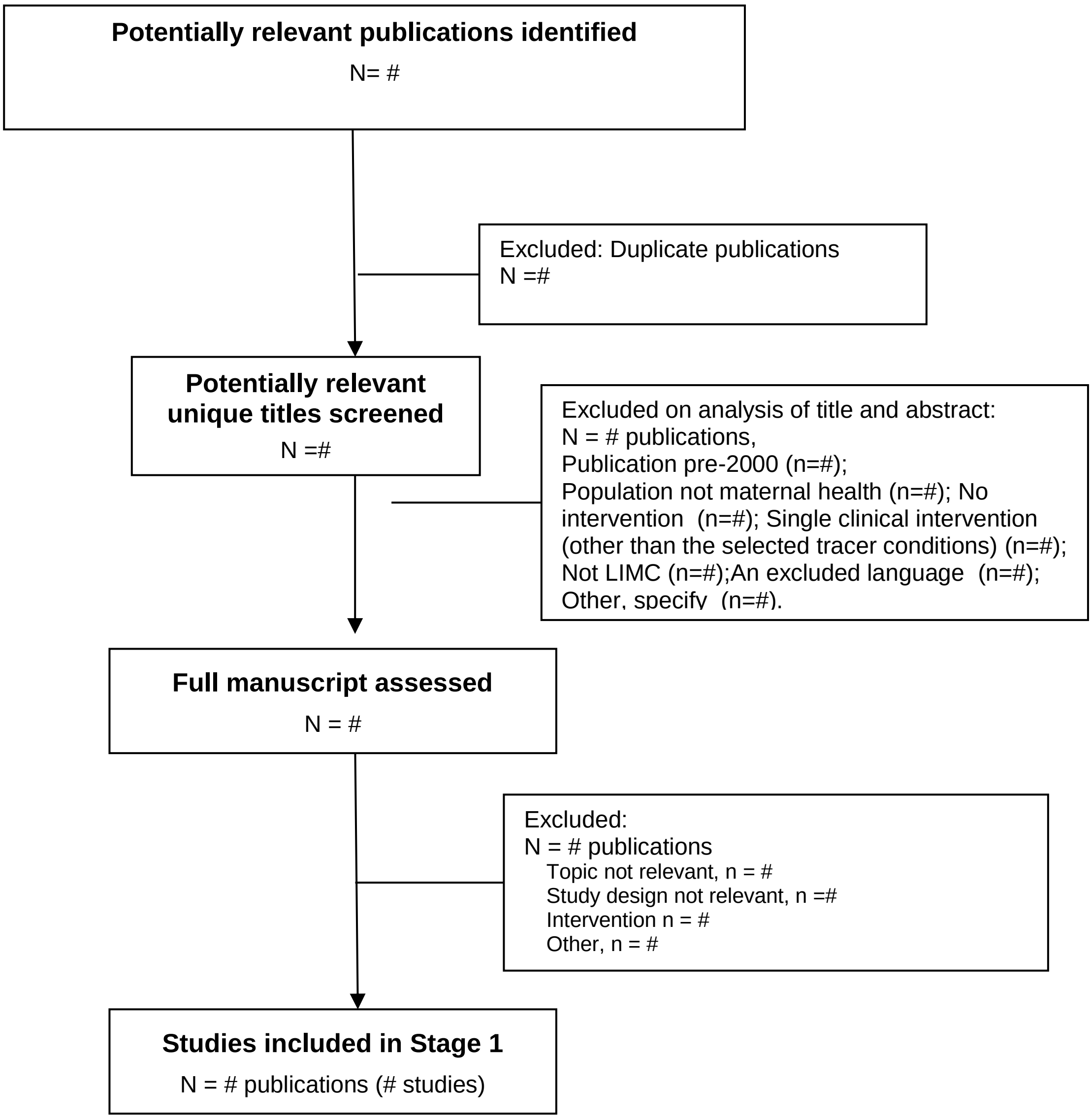

PubMED search strategy

(((((non-pregnancy[All Fields] AND related[All Fields] AND ("infection"[MeSH Terms] OR "infection"[All Fields] OR "communicable diseases"[MeSH Terms] OR ("communicable"[All Fields] AND "diseases"[All Fields]) OR "communicable diseases"[All Fields])) OR non-pregnancy related[Title]) OR ((maternal[Title] OR pregnant[Title] OR pregnancy[Title] OR obstetric[Title] OR puerperal[Title] OR mother[Title] OR childbirth[Title] OR labour[Title] OR labor[Title] OR natal[Title] OR post-natal[Title] OR pre-natal[Title] OR prenatal[Title] OR antenatal[Title] OR ante-natal[Title] OR perinatal[Title] OR peri-natal[Title] OR puerperal[Title] OR puerperium[Title]) AND ((((((sepsis[Title] OR septic$[Title]) OR infection$[Title]) OR HIV[Title]) OR tuberculosis[Title]) OR pneumonia[Title]) OR meningitis[Title]))) OR (chorioamnionitis[Title/Abstract] OR "chorioamnionitis"[MeSH Terms])) OR ((("sepsis"[MeSH Terms] OR "sepsis"[All Fields]) OR septic$[All Fields] OR infection$[Title]) AND ((amniotic[Title/Abstract] OR intra-amniotic[Title/Abstract]) OR intraamniotic[Title/Abstract])))) OR ((((anemic[Title] OR anaemia[Title]) OR anaemic[Title]) OR anemia[Title]) AND (puerperal[Title] OR (((((maternal[Title] OR pregnant[Title]) OR pregnancy[Title]) OR obstetric[Title]) OR mother[Title]) OR childbirth[Title])))) OR ((((("Midwifery"[Mesh] OR dula[Title/Abstract]) OR ((("parturition"[MeSH Terms] OR "parturition"[All Fields] OR "birth"[All Fields]) AND attendant[All Fields]) OR (("parturition"[MeSH Terms] OR "parturition"[All Fields] OR "birth"[All Fields]) AND attendants[All Fields]))) OR ("residence characteristics"[MeSH Terms] OR ("residence"[All Fields] AND "characteristics"[All Fields]) OR "residence characteristics"[All Fields] OR ("place"[All Fields] AND "birth"[All Fields]) OR "place of birth"[All Fields])) OR (("Birthing Centers"[MAJR] OR "Delivery Rooms"[MAJR]) OR "Delivery, Obstetric/nursing"[MAJR])) OR ((maternal[Title] OR pregnant[Title] OR pregnancy[Title] OR obstetric[Title] OR puerperal[Title] OR mother[Title] OR childbirth[Title] OR labour[Title] OR labor[Title] OR natal[Title] OR post-natal[Title] OR pre-natal[Title] OR prenatal[Title] OR antenatal[Title] OR ante-natal[Title] OR perinatal[Title] OR peri-natal[Title] OR puerperal[Title] OR puerperium[Title]) AND (("Ambulances"[Mesh] OR "Health Services Accessibility"[Mesh]) OR "Transportation of Patients"[Mesh]))) OR (("Travel"[MeSH Terms] OR "Delivery of Health Care/organization and administration"[MAJR]) AND (maternal[Title] OR pregnant[Title] OR pregnancy[Title] OR obstetric[Title] OR puerperal[Title] OR mother[Title] OR childbirth[Title] OR labour[Title] OR labor[Title] OR natal[Title] OR post-natal[Title] OR pre-natal[Title] OR prenatal[Title] OR antenatal[Title] OR ante-natal[Title] OR perinatal[Title] OR peri-natal[Title] OR puerperal[Title] OR puerperium[Title])))) OR (ectopic pregnancy[Title/Abstract] OR "pregnancy, ectopic"[MeSH Terms])) OR (((((("Postpartum Hemorrhage"[Mesh] OR (((((((((((((((maternal[Title] OR pregnant[Title]) OR pregnancy[Title]) OR obstetric[Title]) OR puerperal[Title]) OR mother[Title]) OR childbirth[Title]) OR labour[Title]) OR labor[Title]) OR natal[Title]) OR post-natal[Title]) OR pre-natal[Title]) OR prenatal[Title]) OR antenatal[Title]) OR ante-natal[Title]) OR perinatal[Title]) OR peri-natal[Title]) AND (Hemorrhage[Title] OR Haemorrhage[Title]))) OR (((obstetric[All Fields] AND ("haemorrhage"[All Fields] OR "hemorrhage"[MeSH Terms] OR "hemorrhage"[All Fields])) OR obstetric hemorrhage[Title/Abstract]) OR ("postpartum hemorrhage"[MeSH Terms] OR ("postpartum"[All Fields] AND "hemorrhage"[All Fields]) OR "postpartum hemorrhage"[All Fields] OR ("post"[All Fields] AND "partum"[All Fields] AND "hemorrhage"[All Fields]) OR "post partum hemorrhage"[All Fields]) OR ("postpartum hemorrhage"[MeSH Terms] OR ("postpartum"[All Fields] AND "hemorrhage"[All Fields]) OR "postpartum hemorrhage"[All Fields] OR ("post"[All Fields] AND "partum"[All Fields] AND "haemorrhage"[All Fields]) OR "post partum haemorrhage"[All Fields]) OR ("postpartum hemorrhage"[MeSH Terms] OR ("postpartum"[All Fields] AND "hemorrhage"[All Fields]) OR "postpartum hemorrhage"[All Fields] OR ("post"[All Fields] AND "partum"[All Fields] AND "hemorrhage"[All Fields]) OR "post partum hemorrhage"[All Fields]) OR ("postpartum hemorrhage"[MeSH Terms] OR ("postpartum"[All Fields] AND "hemorrhage"[All Fields]) OR "postpartum hemorrhage"[All Fields] OR ("post"[All Fields] AND "partum"[All Fields] AND "haemorrhage"[All Fields]) OR "post partum haemorrhage"[All Fields]))) OR obstetric hemorrhage[Title/Abstract]) OR "Hypertension, Pregnancy-Induced"[Mesh]) OR ((((obstructed labor[Title/Abstract] OR obstructed labour[Title/Abstract]) OR (obstetric fistula[Title/Abstract] OR obstetric fistulae[Title/Abstract])) OR ("vaginal fistula"[MeSH Terms] OR "vesicovaginal fistula"[MeSH Terms])) OR ("Obstetric Labor Complications"[Mesh] OR "Obstetric Labor, Premature"[Mesh]))) OR ((((((((((((((((maternal[Title] OR pregnant[Title]) OR pregnancy[Title]) OR obstetric[Title]) OR puerperal[Title]) OR mother[Title]) OR childbirth[Title]) OR labour[Title]) OR labor[Title]) OR natal[Title]) OR post-natal[Title]) OR pre-natal[Title]) OR prenatal[Title]) OR antenatal[Title]) OR ante-natal[Title]) OR perinatal[Title]) OR peri-natal[Title]) AND (hypertension[Title] OR blood pressure[Title])) AND (((((eclampsia[Title/Abstract] OR preeclampsia[Title/Abstract]) OR HELLP[Title/Abstract]) OR "eclampsia"[MeSH Terms]) OR "pre-eclampsia"[MeSH Terms]) OR pre-eclampsia[Title/Abstract])))) OR ("Pregnancy Complications, Hematologic"[Mesh] OR "Pregnancy in Adolescence"[Mesh] OR "Pregnancy Complications, Infectious"[Mesh] OR "Pregnancy Complications, Cardiovascular"[Mesh] OR "Pregnancy Complications"[Mesh] OR "Pregnancy, Prolonged"[Mesh])) AND (((("africa"[MeSH Terms] OR "africa"[All Fields]) OR ((((((("afghanistan"[MeSH Terms] OR "afghanistan"[All Fields]) OR ("bangladesh"[MeSH Terms] OR "bangladesh"[All Fields])) OR ("benin"[MeSH Terms] OR "benin"[All Fields])) OR ("burkina faso"[MeSH Terms] OR ("burkina"[All Fields] AND "faso"[All Fields]) OR "burkina faso"[All Fields])) OR ((((((((((((((((((((((((((("burundi"[MeSH Terms] OR "burundi"[All Fields]) OR ("cambodia"[MeSH Terms] OR "cambodia"[All Fields])) OR ("central african republic"[MeSH Terms] OR ("central"[All Fields] AND "african"[All Fields] AND "republic"[All Fields]) OR "central african republic"[All Fields])) OR ("chad"[MeSH Terms] OR "chad"[All Fields])) OR ("comoros"[MeSH Terms] OR "comoros"[All Fields])) OR (("congo"[MeSH Terms] OR "congo"[All Fields]) AND Dem.[All Fields] AND Rep[All Fields])) OR ("congo"[MeSH Terms] OR "congo"[All Fields])) OR DRC[Affiliation]) OR ("eritrea"[MeSH Terms] OR "eritrea"[All Fields])) OR ("ethiopia"[MeSH Terms] OR "ethiopia"[All Fields])) OR ("gambia"[MeSH Terms] OR "gambia"[All Fields])) OR ("guinea"[MeSH Terms] OR "guinea"[All Fields])) OR (("guinea"[MeSH Terms] OR "guinea"[All Fields]) AND Bisau[All Fields])) OR ("haiti"[MeSH Terms] OR "haiti"[All Fields])) OR ("kenya"[MeSH Terms] OR "kenya"[All Fields])) OR ("korea"[MeSH Terms] OR "korea"[All Fields])) OR Kyrgyz[All Fields]) OR ("liberia"[MeSH Terms] OR "liberia"[All Fields])) OR ("madagascar"[MeSH Terms] OR "madagascar"[All Fields])) OR ("malawi"[MeSH Terms] OR "malawi"[All Fields])) OR ("mali"[MeSH Terms] OR "mali"[All Fields])) OR ("mozambique"[MeSH Terms] OR "mozambique"[All Fields])) OR ("myanmar"[MeSH Terms] OR "myanmar"[All Fields])) OR ("nepal"[MeSH Terms] OR "nepal"[All Fields])) OR ("niger"[MeSH Terms] OR "niger"[All Fields])) OR ("rwanda"[MeSH Terms] OR "rwanda"[All Fields])) OR ("sierra leone"[MeSH Terms] OR ("sierra"[All Fields] AND "leone"[All Fields]) OR "sierra leone"[All Fields])) OR ("somalia"[MeSH Terms] OR "somalia"[All Fields]))) OR ((((("tajikistan"[MeSH Terms] OR "tajikistan"[All Fields]) OR ("tanzania"[MeSH Terms] OR "tanzania"[All Fields])) OR ("togo"[MeSH Terms] OR "togo"[All Fields])) OR ("uganda"[MeSH Terms] OR "uganda"[All Fields])) OR ("zimbabwe"[MeSH Terms] OR "zimbabwe"[All Fields]))) OR ("africa, northern"[MeSH Terms] OR ("africa"[All Fields] AND "northern"[All Fields]) OR "northern africa"[All Fields] OR "sahara"[All Fields])) OR sub-saharan[All Fields])) OR (("angola"[MeSH Terms] OR "angola"[All Fields]) OR ("armenia"[MeSH Terms] OR "armenia"[All Fields])) OR ("belize"[MeSH Terms] OR "belize"[All Fields])) OR ("bhutan"[MeSH Terms] OR "bhutan"[All Fields])) OR ("bolivia"[MeSH Terms] OR "bolivia"[All Fields])) OR ("cameroon"[MeSH Terms] OR "cameroon"[All Fields])) OR ("cape verde"[MeSH Terms] OR ("cape"[All Fields] AND "verde"[All Fields]) OR "cape verde"[All Fields])) OR ("congo"[MeSH Terms] OR "congo"[All Fields])) OR ("cote d'ivoire"[MeSH Terms] OR ("cote"[All Fields] AND "d'ivoire"[All Fields]) OR "cote d'ivoire"[All Fields])) OR ("cote d'ivoire"[MeSH Terms] OR ("cote"[All Fields] AND "d'ivoire"[All Fields]) OR "cote d'ivoire"[All Fields] OR ("ivory"[All Fields] AND "coast"[All Fields]) OR "ivory coast"[All Fields])) OR ("djibouti"[MeSH Terms] OR "djibouti"[All Fields])) OR ("egypt"[MeSH Terms] OR "egypt"[All Fields])) OR ("el salvador"[MeSH Terms] OR ("el"[All Fields] AND "salvador"[All Fields]) OR "el salvador"[All Fields])) OR ("fiji"[MeSH Terms] OR "fiji"[All Fields])) OR ("georgia"[MeSH Terms] OR "georgia"[All Fields] OR "georgia (republic)"[MeSH Terms] OR ("georgia"[All Fields] AND "(republic)"[All Fields]) OR "georgia (republic)"[All Fields])) OR ("ghana"[MeSH Terms] OR "ghana"[All Fields])) OR ("guatemala"[MeSH Terms] OR "guatemala"[All Fields])) OR ("guyana"[MeSH Terms] OR "guyana"[All Fields])) OR ("honduras"[MeSH Terms] OR "honduras"[All Fields])) OR ("indonesia"[MeSH Terms] OR "indonesia"[All Fields])) OR ("india"[MeSH Terms] OR "india"[All Fields])) OR ("iraq"[MeSH Terms] OR "iraq"[All Fields])) OR ("micronesia"[MeSH Terms] OR "micronesia"[All Fields] OR "kiribati"[All Fields])) OR ("yugoslavia"[MeSH Terms] OR "yugoslavia"[All Fields] OR "kosovo"[All Fields])) OR Lao[All Fields]) OR ("lesotho"[MeSH Terms] OR "lesotho"[All Fields])) OR ("micronesia"[MeSH Terms] OR "micronesia"[All Fields] OR ("marshall"[All Fields] AND "islands"[All Fields]) OR "marshall islands"[All Fields])) OR ("mauritania"[MeSH Terms] OR "mauritania"[All Fields])) OR ("micronesia"[MeSH Terms] OR "micronesia"[All Fields])) OR ("moldova"[MeSH Terms] OR "moldova"[All Fields])) OR ("mongolia"[MeSH Terms] OR "mongolia"[All Fields])) OR ("morocco"[MeSH Terms] OR "morocco"[All Fields])) OR

("nicaragua"[MeSH Terms] OR "nicaragua"[All Fields])) OR ("nigeria"[MeSH Terms] OR "nigeria"[All Fields])) OR ("pakistan"[MeSH Terms] OR "pakistan"[All Fields])) OR ("papua new guinea"[MeSH Terms] OR ("papua"[All Fields] AND "new"[All Fields] AND "guinea"[All Fields]) OR "papua new guinea"[All Fields])) OR ("paraguay"[MeSH Terms] OR "paraguay"[All Fields])) OR ("philippines"[MeSH Terms] OR "philippines"[All Fields])) OR ("samoa"[MeSH Terms] OR "samoa"[All Fields])) OR ("atlantic islands"[MeSH Terms] OR ("atlantic"[All Fields] AND "islands"[All Fields]) OR "atlantic islands"[All Fields] OR ("sao"[All Fields] AND "tome"[All Fields] AND "principe"[All Fields]) OR "sao tome and principe"[All Fields])) OR (Sao[All Fields] AND Tome[All Fields])) OR ("senegal"[MeSH Terms] OR "senegal"[All Fields])) OR ("melanesia"[MeSH Terms] OR "melanesia"[All Fields] OR ("solomon"[All Fields] AND "islands"[All Fields]) OR "solomon islands"[All Fields])) OR ("sri lanka"[MeSH Terms] OR ("sri"[All Fields] AND "lanka"[All Fields]) OR "sri lanka"[All Fields])) OR ("sudan"[MeSH Terms] OR "sudan"[All Fields])) OR ("swaziland"[MeSH Terms] OR "swaziland"[All Fields])) OR ("syria"[MeSH Terms] OR "syria"[All Fields] OR ("syrian"[All Fields] AND "arab"[All Fields] AND "republic"[All Fields]) OR "syrian arab republic"[All Fields])) OR ("syria"[MeSH Terms] OR "syria"[All Fields])) OR ("east timor"[MeSH Terms] OR ("east"[All Fields] AND "timor"[All Fields]) OR "east timor"[All Fields] OR ("timor"[All Fields] AND "leste"[All Fields]) OR "timor leste"[All Fields])) OR ("indonesia"[MeSH Terms] OR "indonesia"[All Fields] OR "timor"[All Fields])) OR ("tonga"[MeSH Terms] OR "tonga"[All Fields])) OR ("turkmenistan"[MeSH Terms] OR "turkmenistan"[All Fields])) OR ("micronesia"[MeSH Terms] OR "micronesia"[All Fields] OR "tuvalu"[All Fields])) OR ("ukraine"[MeSH Terms] OR "ukraine"[All Fields])) OR ("uzbekistan"[MeSH Terms] OR "uzbekistan"[All Fields])) OR ("vanuatu"[MeSH Terms] OR "vanuatu"[All Fields])) OR ("vietnam"[MeSH Terms] OR "vietnam"[All Fields])) OR (("middle east"[MeSH Terms] OR ("middle"[All Fields] AND "east"[All Fields]) OR "middle east"[All Fields] OR ("west"[All Fields] AND "bank"[All Fields]) OR "west bank"[All Fields]) AND Gaza[All Fields])) OR Gaza[All Fields]) OR ("yemen"[MeSH Terms] OR "yemen"[All Fields])) OR ("zambia"[MeSH Terms] OR "zambia"[All Fields]))) OR (((((((((((((((((((((("albania"[MeSH Terms] OR "albania"[All Fields]) OR ("algeria"[MeSH Terms] OR "algeria"[All Fields])) OR ("american samoa"[MeSH Terms] OR ("american"[All Fields] AND "samoa"[All Fields]) OR "american samoa"[All Fields])) OR ("samoa"[MeSH Terms] OR "samoa"[All Fields])) OR ("antigua and barbuda"[MeSH Terms] OR ("antigua"[All Fields] AND "barbuda"[All Fields]) OR "antigua and barbuda"[All Fields])) OR ("antigua and barbuda"[MeSH Terms] OR ("antigua" [All Fields] AND "barbuda"[All Fields]) OR "antigua and barbuda"[All Fields] OR "antigua"[All Fields])) OR ("antigua and barbuda"[MeSH Terms] OR ("antigua"[All Fields] AND "barbuda"[All Fields]) OR "antigua and barbuda"[All Fields] OR "barbuda"[All Fields])) OR ("argentina"[MeSH Terms] OR "argentina"[All Fields])) OR ("azerbaijan"[MeSH Terms] OR "azerbaijan"[All Fields])) OR ("republic of belarus"[MeSH Terms] OR ("republic"[All Fields] AND "belarus"[All Fields]) OR "republic of belarus"[All Fields] OR "belarus"[All Fields])) OR ("bosnia-herzegovina"[MeSH Terms] OR "bosnia-herzegovina"[All Fields] OR ("bosnia"[All Fields] AND "herzegovina"[All Fields]) OR "bosnia and herzegovina"[All Fields])) OR ("bosnia-herzegovina"[MeSH Terms] OR "bosnia-herzegovina"[All Fields] OR "bosnia"[All Fields])) OR ("bosnia-herzegovina"[MeSH Terms] OR "bosnia-herzegovina"[All Fields] OR "herzegovina"[All Fields])) OR ("botswana"[MeSH Terms] OR "botswana"[All Fields])) OR ("brazil"[MeSH Terms] OR "brazil"[All Fields])) OR ("bulgaria"[MeSH Terms] OR "bulgaria"[All Fields])) OR ("chile"[MeSH Terms] OR "chile"[All Fields])) OR ("china"[MeSH Terms] OR "china"[All Fields])) OR ("colombia"[MeSH Terms] OR "colombia"[All Fields])) OR ("costa rica"[MeSH Terms] OR ("costa"[All Fields] AND "rica"[All Fields]) OR "costa rica"[All Fields])) OR ("cuba"[MeSH Terms] OR "cuba"[All Fields])) OR ("dominica"[MeSH Terms] OR "dominica"[All Fields])) OR ("dominican republic"[MeSH Terms] OR ("dominican"[All Fields] AND "republic"[All Fields]) OR "dominican republic"[All Fields])) OR ("ecuador"[MeSH Terms] OR "ecuador"[All Fields])) OR ("gabon"[MeSH Terms] OR "gabon"[All Fields])) OR ("grenada"[MeSH Terms] OR "grenada"[All Fields])) OR ("iran"[MeSH Terms] OR "iran"[All Fields])) OR ("jamaica"[MeSH Terms] OR "jamaica"[All Fields])) OR ("jordan"[MeSH Terms] OR "jordan"[All Fields])) OR ("kazakhstan"[MeSH Terms] OR "kazakhstan"[All Fields])) OR ("latvia"[MeSH Terms] OR "latvia"[All Fields])) OR ("lebanon"[MeSH Terms] OR "lebanon"[All Fields])) OR ("libya"[MeSH Terms] OR "libya"[All Fields])) OR ("lithuania"[MeSH Terms] OR "lithuania"[All Fields])) OR ("macedonia (republic)"[MeSH Terms] OR ("macedonia"[All Fields] AND "(republic)"[All Fields]) OR "macedonia (republic)"[All Fields] OR "macedonia"[All Fields])) OR ("malaysia"[MeSH Terms] OR "malaysia"[All Fields])) OR ("indian ocean islands"[MeSH Terms] OR ("indian"[All Fields] AND "ocean"[All Fields] AND "islands"[All Fields]) OR "indian ocean islands"[All Fields] OR "maldives"[All Fields])) OR ("mauritius"[MeSH Terms] OR "mauritius"[All Fields])) OR ("comoros"[MeSH Terms] OR "comoros"[All Fields] OR "mayotte"[All Fields])) OR ("mexico"[MeSH Terms] OR "mexico"[All Fields])) OR ("montenegro"[MeSH Terms] OR "montenegro"[All Fields])) OR ("namibia"[MeSH Terms] OR "namibia"[All Fields])) OR ("palau"[MeSH Terms] OR "palau"[All Fields])) OR ("panama"[MeSH Terms] OR "panama"[All Fields])) OR ("peru"[MeSH Terms] OR "peru"[All Fields])) OR ("romania"[MeSH Terms] OR "romania"[All Fields])) OR ("russia"[MeSH Terms] OR "russia"[All Fields] OR ("russian"[All Fields] AND "federation"[All Fields]) OR "russian federation"[All Fields])) OR ("russia"[MeSH Terms] OR "russia"[All Fields])) OR ("ussr"[MeSH Terms] OR "ussr"[All Fields])) OR ("serbia"[MeSH Terms] OR "serbia"[All Fields])) OR ("seychelles"[MeSH Terms] OR "seychelles"[All Fields])) OR ("south africa"[MeSH Terms] OR ("south"[All Fields] AND "africa"[All Fields]) OR "south africa"[All Fields])) OR ("saint kitts and nevis"[MeSH Terms] OR ("saint"[All Fields] AND "kitts"[All Fields] AND "nevis"[All Fields]) OR "saint kitts and nevis"[All Fields] OR ("st"[All Fields] AND "kitts"[All Fields] AND "nevis"[All Fields]) OR "st kitts and nevis"[All Fields])) OR ("saint kitts and nevis"[MeSH Terms] OR ("saint"[All Fields] AND "kitts"[All Fields] AND "nevis"[All Fields]) OR "saint kitts and nevis"[All Fields])) OR (("saints"[MeSH Terms] OR "saints"[All Fields] OR "saint"[All Fields]) AND Kitts[All Fields])) OR ("saint lucia"[MeSH Terms] OR ("saint"[All Fields] AND "lucia"[All Fields]) OR "saint lucia"[All Fields] OR ("st"[All Fields] AND "lucia"[All Fields]) OR "st lucia"[All Fields])) OR ("saint lucia"[MeSH Terms] OR ("saint"[All Fields] AND "lucia"[All Fields]) OR "saint lucia"[All Fields])) OR ("saint vincent and the grenadines"[MeSH Terms] OR ("saint"[All Fields] AND "vincent"[All Fields] AND "grenadines"[All Fields]) OR "saint vincent and the grenadines"[All Fields] OR ("st"[All Fields] AND "vincent"[All Fields] AND "grenadines"[All Fields]) OR "st vincent and the grenadines"[All Fields])) OR (St. Vincent[Author] OR St. Vincent[Investigator])) OR ("saint vincent and the grenadines"[MeSH Terms] OR ("saint"[All Fields] AND "vincent"[All Fields] AND "grenadines"[All Fields]) OR "saint vincent and the grenadines"[All Fields])) OR Saint Vincent[Author]) OR ("saint vincent and the grenadines"[MeSH Terms] OR ("saint"[All Fields] AND "vincent"[All Fields] AND "grenadines"[All Fields]) OR "saint vincent and the grenadines"[All Fields] OR "grenadines"[All Fields])) OR ("suriname"[MeSH Terms] OR "suriname"[All Fields])) OR ("thailand"[MeSH Terms] OR "thailand"[All Fields])) OR ("tunisia"[MeSH Terms] OR "tunisia"[All Fields])) OR ("turkey"[MeSH Terms] OR "turkey"[All Fields])) OR ("uruguay"[MeSH Terms] OR "uruguay"[All Fields])) OR ("venezuela"[MeSH Terms] OR "venezuela"[All Fields]))))

CINAHL search strategy

#	Query	Limiters/Expanders
S44	S39 and S43	Search modes - Boolean/Phrase<br>Limiters - Published Date from: 20000101-20121231
S43	S40 or S41 or S42	Search modes - Boolean/Phrase
S42	Albania OR Algeria OR Samoa OR Antigua OR Barbuda OR Argentina OR Azerbaijan OR Belarus OR Bosnia OR Herzegovina OR Botswana OR Brazil OR Bulgaria OR Chile OR China OR Colombia OR Costa Rica OR Cuba OR Dominica OR Dominican Republic OR Ecuador OR Gabon OR Grenada OR Iran OR Jamaica OR Jordan OR Kazakhstan OR Latvia OR Lebanon OR Libya OR Lithuania OR Macedonia OR Malaysia OR Maldives OR	Search modes - Boolean/Phrase

	Mauritius OR Mayotte OR Mexico OR Montenegro OR Namibia OR Palau OR Panama OR Peru OR Romania OR Russian Federation OR SerbiaOR Seychelles OR South Africa OR St. Kitts and Nevis OR St. Lucia OR St. Vincent OR Grenadines OR Suriname OR Thailand OR Tunisia OR Turkey OR Uruguay OR Venezuela	
S41	Angola OR Armenia OR Belize OR Bhutan OR Bolivia OR Cameroon OR Cape Verde OR Congo, Rep OR Côte d'Ivoire OR Djibouti OR Egypt OR El Salvador OR Fiji OR Georgia OR Ghana OR Guatemala OR Guyana OR Honduras OR Indonesia OR India OR Iraq OR Kiribati OR Kosovo OR Lao PDR OR Lesotho OR Marshall Islands OR Mauritania OR Micronesia OR Moldova OR Mongolia OR Morocco OR Nicaragua OR Nigeria OR Pakistan OR Papua New Guinea OR Paraguay OR Philippines OR Samoa OR São Tomé and Principe OR Senegal OR Solomon Islands OR Sri Lanka OR Sudan OR Swaziland OR Syria* OR Timor-Leste OR Tonga OR Turkmenistan OR Tuvalu OR Ukraine OR Uzbekistan OR Vanuatu OR Vietnam OR Gaza OR Yemen OR Zambia	Search modes - Boolean/Phrase
S40	Afghanistan OR Bangladesh OR Benin OR Burkina Faso OR Burundi OR Cambodia OR Central African Republic OR Chad OR Comoros OR Congo, Dem. Rep OR Eritrea OR Ethiopia OR Gambia, The OR Guinea OR Guinea-Bisau OR Haiti OR Kenya OR Korea, Dem Rep OR Kyrgyz Republic OR Liberia OR Madagascar OR Malawi OR Mali OR Mozambique OR Myanmar OR Nepal OR Niger OR Rwanda OR Sierra Leone OR Somalia OR Tajikistan OR Tanzania OR Togo OR Uganda OR Zimbabwe	Search modes - Boolean/Phrase
S39	S8 or S13 or S19 or S25 or S38	Search modes - Boolean/Phrase
S38	S26 or S27 or S28 or S29 or S30 or S31 or S32 or S33 or S34 or S35 or S36 or S37	Search modes - Boolean/Phrase
S37	traditional birth attendant	Search modes - Boolean/Phrase
S36	(attend* OR unattend*) N2 (birth* OR delivery or labo#r)	Search modes - Boolean/Phrase
S35	unattended birth	Search modes - Boolean/Phrase
S34	(MH "Lay Midwives") OR "birth attendant"	Search modes - Boolean/Phrase
S33	(MH "Delivery Rooms") OR (MH "Alternative Birth Centers")	Search modes - Boolean/Phrase
S32	(MH "Pregnancy, Ectopic") OR (MH "Pregnancy Complications, Cardiovascular+") OR (MH "Pregnancy Complications, Neoplastic+") OR (MH "Puerperal Disorders+")	Search modes - Boolean/Phrase
S31	(MH "Intrapartum Care") OR (MH "Obstetric Care") OR (MH "Delivery")	Search modes - Boolean/Phrase
S30	MM "Management of Labor"	Search modes - Boolean/Phrase
S29	(pro#long* OR obstruct*) N2 (deliver* OR labo#r)	Search modes - Boolean/Phrase
S28	"obstructed labor"	Search modes - Boolean/Phrase
S27	Miscarriage	Search modes - Boolean/Phrase
S26	(MH "Abortion, Spontaneous")	Search modes - Boolean/Phrase
S25	S20 or S21 or S22 or S23 or S24	Search modes - Boolean/Phrase
S24	pre#eclampsia	Search modes - Boolean/Phrase
S23	HELLP	Search modes - Boolean/Phrase
S22	(MH "Eclampsia+") OR (MH "Pre-Eclampsia+")	Search modes - Boolean/Phrase
S21	Eclampsia	Search modes - Boolean/Phrase
S20	(MH "Pregnancy-Induced Hypertension")	Search modes - Boolean/Phrase
S19	S14 or S15 or S17 or S18	Search modes - Boolean/Phrase
S18	"post#partum h#emorrhage"	Search modes - Boolean/Phrase
S17	S2 N2 S16	Search modes - Boolean/Phrase
S16	h#emorrhage	Search modes - Boolean/Phrase
S15	(MH "Postpartum Hemorrhage")	Search modes - Boolean/Phrase
S14	postpartum hemorrhage	Search modes - Boolean/Phrase
S13	S9 or S10 or S12	Search modes - Boolean/Phrase

S12	S2 N2 S11	Search modes - Boolean/Phrase
S11	an#emia	Search modes - Boolean/Phrase
S10	MM "Pregnancy Complications, Hematologic"	Search modes - Boolean/Phrase
S9	maternal anemia	Search modes - Boolean/Phrase
S8	S1 or S4 or S5 or S6 or S7	Search modes - Boolean/Phrase
S7	MM "Pregnancy Complications, Infectious"	Search modes - Boolean/Phrase
S6	infection in pregnancy	Search modes - Boolean/Phrase
S5	"maternal infection"	Search modes - Boolean/Phrase
S4	S2 N2 S3	Search modes - Boolean/Phrase
S3	(infect* OR sepsis OR septic OR tubercul* OR pneumonia OR meningitis OR HIV)	Search modes - Boolean/Phrase
S2	(pregnan* OR maternal OR obstetric* OR puerper* OR partum OR birth OR childbirth)	Search modes - Boolean/Phrase
S1	(MH "Chorioamnionitis")	Search modes - Boolean/Phrase

Embase search strategy

1	(Albania or Algeria or Samoa or Antigua or Barbuda or Argentina or Azerbaijan or Belarus or Bosnia or Herzegovina or Botswana or Brazil or Bulgaria or Chile or China or Colombia or Costa Rica or Cuba or Dominica or Dominican Republic or Ecuador or Gabon or Grenada or Iran or Jamaica or Jordan or Kazakhstan or Latvia or Lebanon or Libya or Lithuania or Macedonia or Malaysia or Maldives or Mauritius or Mayotte or Mexico or Montenegro or Namibia or Palau or Panama or Peru or Romania or Russian Federation or Russia or Serbia or Seychelles or South Africa or St Kitts or Nevis or St Lucia or St Vincent or Grenadines or Suriname or Thailand or Tunisia or Turkey or Uruguay or Venezuela).mp. [mp=title, abstract, subject headings, heading word, drug trade name, original title, device manufacturer, drug manufacturer, device trade name, keyword]
2	(Angola or Armenia or Belize or Bhutan or Bolivia or Cameroon or Cape Verde or Congo, or Cote d'Ivoire or Ivory Coast or Djibouti or Egypt or Arab Republic or El Salvador or Fiji or Georgia or Ghana or Guatemala or Guyana or Honduras or Indonesia or India or Iraq or Kiribati or Kosovo or Lao PDR or Lesotho or Marshall Islands or Mauritania or Micronesia or Moldova or Mongolia or Morocco or Nicaragua or Nigeria or Pakistan or Papua New Guinea or Paraguay or Philippines or Samoa or Sao Tome or Principe or Senegal or Solomon Islands or Sri Lanka or Sudan or Swaziland or Syrian Arab Republic or Timor-Leste or Tonga or Turkmenistan or Tuvalu or Ukraine or Uzbekistan or Vanuatu or Vietnam or West Bank or Gaza or Yemen or Zambia).mp. [mp=title, abstract, subject headings, heading word, drug trade name, original title, device manufacturer, drug manufacturer, device trade name, keyword]
3	(Afghanistan or Bangladesh or Benin or Burkina Faso or Burundi or Cambodia or Central African Republic or Chad or Comoros or Congo or Eritrea or Ethiopia or Gambia or Guinea or Bisau or Haiti or Kenya or Korea or Kyrgyz or Liberia or Madagascar or Malawi or Mali or Mozambique or Myanmar or Nepal or Niger or Rwanda or Sierra Leone or Somalia or Tajikistan or Tanzania or Togo or Uganda or Zimbabwe).mp. [mp=title, abstract, subject headings, heading word, drug trade name, original title, device manufacturer, drug manufacturer, device trade name, keyword]
4	(Africa or sahara* or low income country or low income countries or middle income country or middle income countries or south america or central america or latin america or carribean).mp. [mp=title, abstract, subject headings, heading word, drug trade name, original title, device manufacturer, drug manufacturer, device trade name, keyword]
5	exp Developing Countries/
6	(#1 or #2 or #3 or #4 or #5).mp. [mp=title, abstract, subject headings, heading word, drug trade name, original title, device manufacturer, drug manufacturer, device trade name, keyword]
7	limit 6 to (human and yr="2000 -Current")
8	maternal infection.mp.
9	chorioamnionitis.mp.
10	exp maternal disease/ or exp intrauterine infection/
11	(pregnan* or maternal or obstetric* or puerper* or partum or birth or childbirth or prenatal or postnatal or natal).mp. [mp=title, abstract, subject headings, heading word, drug trade name, original title, device manufacturer, drug manufacturer, device trade name, keyword]
12	(infect* or sepsis or septic or tubercul* or pneumonia or meningitis or HIV).mp. [mp=title, abstract, subject headings, heading word, drug trade name, original title, device manufacturer, drug manufacturer, device trade name, keyword]
13	(#11 adj3 #12).mp. [mp=title, abstract, subject headings, heading word, drug trade name, original title, device manufacturer, drug manufacturer, device trade name, keyword]
14	maternal anemia.mp.
15	exp PREGNANCY COMPLICATIONS, HEMATOLOGIC/
16	(anemi* or anaemi* or hemoglobin or haemoglobin).mp. [mp=title, abstract, subject headings, heading word, drug trade name, original title, device manufacturer, drug manufacturer, device trade name, keyword]
17	(#11 adj3 #16).mp. [mp=title, abstract, subject headings, heading word, drug trade name, original title, device manufacturer, drug manufacturer, device trade name, keyword]
18	exp postpartum hemorrhage/
19	((maternal or obstetric* or puerper* or partum or birth or childbirth or postnatal) adj2 (bleed or bleeding or hemorrhage or haemorrhage)).mp. [mp=title, abstract, subject headings, heading word, drug trade name, original title, device manufacturer, drug manufacturer, device trade name, keyword]
20	exp "eclampsia and preeclampsia"/ or exp eclampsia/
21	(eclampsia or pre-ecalmapsia or preeclampsia or HELLP).ti,ab.
22	miscarriage.ti,ab.
23	exp SPONTANEOUS ABORTION/
24	obstructed labor.mp.
25	exp LABOR OBSTRUCTION/
26	((obstruct* or prolong*) adj2 (labour or labor or delivery or birth or childbirth)).ti,ab.

27 exp LABOR MANAGEMENT/

28 exp intrapartum care/

29 exp perinatal care/

30 exp DELIVERY ROOM/

31 exp HOME DELIVERY/

32 exp birthplace/

33 birth attendant*.mp.

34 place* of birth*.mp.

35 ((attend* or unattend* or alone or support) adj2 (Birth* or childbirth* or deliver*)).ti,ab.

36 *MATERNAL CARE/

37 8 or 9 or 10 or 13 or 14 or 15 or 17 or 18 or 19 or 20 or 21 or 22 or 23 or 24 or 25 or 26 or 27 or 28 or 29 or 30 or 31 or 32 or 33 or 34 or 35 or 36

38 clincal trial.mp.

39 phase 1 clinical trial/

40 phase 2 clinical trial/

41 controlled clinical trial/ or clinical trial/ or "controlled clinical trial (topic)"/

42 phase 3 clinical trial/

43 phase 4 clinical trial/

44 38 or 39 or 40 or 41 or 42 or 43

45 7 and 37

46 limit 45 to ((evidence based medicine or meta analysis or outcomes research or "systematic review") and yr="2000 -Current")

PsycINFO search strategy

1 (Albania or Algeria or Samoa or Antigua or Barbuda or Argentina or Azerbaijan or Belarus or Bosnia or Herzegovina or Botswana or Brazil or Bulgaria or Chile or China or Colombia or Costa Rica or Cuba or Dominica or Dominican Republic or Ecuador or Gabon or Grenada or Iran or Jamaica or Jordan or Kazakhstan or Latvia or Lebanon or Libya or Lithuania or Macedonia or Malaysia or Maldives or Mauritius or Mayotte or Mexico or Montenegro or Namibia or Palau or Panama or Peru or Romania or Russian Federation or Russia or Serbia or Seychelles or South Africa or St Kitts or Nevis or St Lucia or St Vincent or Grenadines or Suriname or Thailand or Tunisia or Turkey or Uruguay or Venezuela).mp. [mp=title, abstract, heading word, table of contents, key concepts, original title, tests & measures] (51248)
2 (Angola or Armenia or Belize or Bhutan or Bolivia or Cameroon or Cape Verde or Congo, or Cote d'Ivoire or Ivory Coast or Djibouti or Egypt or Arab Republic or El Salvador or Fiji or Georgia or Ghana or Guatemala or Guyana or Honduras or Indonesia or India or Iraq or Kiribati or Kosovo or Lao PDR or Lesotho or Marshall Islands or Mauritania or Micronesia or Moldova or Mongolia or Morocco or Nicaragua or Nigeria or Pakistan or Papua New Guinea or Paraguay or Philippines or Samoa or Sao Tome or Principe or Senegal or Solomon Islands or Sri Lanka or Sudan or Swaziland or Syrian Arab Republic or Timor-Leste or Tonga or Turkmenistan or Tuvalu or Ukraine or Uzbekistan or Vanuatu or Vietnam or West Bank or Gaza or Yemen or Zambia).mp. [mp=title, abstract, heading word, table of contents, key concepts, original title, tests & measures] (28159)
3 (Afghanistan or Bangladesh or Benin or Burkina Faso or Burundi or Cambodia or Central African Republic or Chad or Comoros or Congo or Eritrea or Ethiopia or Gambia or Guinea or Bisau or Haiti or Kenya or Korea or Kyrgyz or Liberia or Madagascar or Malawi or Mali or Mozambique or Myanmar or Nepal or Niger or Rwanda or Sierra Leone or Somalia or Tajikistan or Tanzania or Togo or Uganda or Zimbabwe).mp. [mp=title, abstract, heading word, table of contents, key concepts, original title, tests & measures] (15936)
4 (Africa or sahara* or low income country or low income countries or middle income country or middle income countries or south america or central america or latin america or carribean).mp. [mp=title, abstract, heading word, table of contents, key concepts, original title, tests & measures] (13920)
5 exp Developing Countries/ (3010)
6 limit 2 to (human and yr="2000 - 2012") (18992)
7 limit 3 to (human and yr="2000 - 2012") (10958)
8 limit 4 to (human and yr="2000 - 2012") (10674)
9 limit 5 to (human and yr="2000 - 2012") (2363)
10 (#1 or #2 or #3 or #4 or #5).mp. [mp=title, abstract, heading word, table of contents, key concepts, original title, tests & measures] (906414)

11 limit 10 to (human and yr="2000 -Current") (510379)
12 maternal infection.mp. (118)
13 chorioamnionitis.mp. (40)
14 exp midwifery/ or exp obstetrical complications/ (1531)
15 miscarriage.mp. or exp Spontaneous Abortion/ (768)
16 (pregnan* or maternal or obstetric* or puerper* or partum or birth or childbirth or prenatal or postnatal or natal or post-partum).mp. [mp=title, abstract, heading word, table of contents, key concepts, original title, tests & measures] (84865)
17 (infect* or sepsis or septic or tubercul* or pneumonia or meningitis or HIV or hemorrhage or haemorrhage or bleed*).mp. [mp=title, abstract, heading word, table of contents, key concepts, original title, tests & measures] (49913)
18 (#18 adj3 #19).mp. [mp=title, abstract, heading word, table of contents, key concepts, original title, tests & measures] (2516)
19 (anemia or anaemia).mp. [mp=title, abstract, heading word, table of contents, key concepts, original title, tests & measures] (1011)
20 (#18 adj3 #21).mp. [mp=title, abstract, heading word, table of contents, key concepts, original title, tests & measures] (1230)
21 ((obstruc* or prolong*) adj3 (labour or labour or birth or delivery)).mp. [mp=title, abstract, heading word, table of contents, key concepts, original title, tests & measures] (43)
22 birth attendant*.mp. [mp=title, abstract, heading word, table of contents, key concepts, original title, tests & measures] (110)
23 childbirth.mp. [mp=title, abstract, heading word, table of contents, key concepts, original title, tests & measures] (2867)
24 *Birth/ (2853)
25 12 or 13 or 14 or 15 or 18 or 20 or 21 or 22 or 23 or 24 (10515)
26 11 and 25 (4257)
27 limit 26 to (human and ("reviews (maximizes sensitivity)" or "therapy (maximizes sensitivity)" or "qualitative (maximizes sensitivity)") and human and yr="2000 -Current") (3171)
28 limit 27 to (120 neonatal <birth to age 1 mo> or 200 adolescence <age 13 to 17 yrs> or 320 young adulthood <age 18 to 29 yrs> or 340 thirties <age 30 to 39 yrs> or 360 middle age <age 40 to 64 yrs>) (1139)
29 limit 28 to (100 childhood <birth to age 12 yrs> or 200 adolescence <age 13 to 17 yrs> or 320 young adulthood <age 18 to 29 yrs> or 340 thirties <age 30 to 39 yrs> or 360 middle age <age 40 to 64 yrs>) (1139)
30 limit 29 to yr="2000 - 2005" (339)
31 limit 29 to yr="2006 -Current" (800)

Web of Knowledge search strategy

Set	Results	Search terms
# 69	**8,903**	#59 AND #6<br>Refined by: Web of Science Categories=(PUBLIC ENVIRONMENTAL OCCUPATIONAL HEALTH OR INFECTIOUS DISEASES OR OBSTETRICS GYNECOLOGY OR MEDICINE GENERAL INTERNAL OR TROPICAL MEDICINE OR SOCIAL SCIENCES BIOMEDICAL OR HEALTH POLICY SERVICES OR NURSING OR MEDICINE RESEARCH EXPERIMENTAL)<br>*Databases=SCI-EXPANDED, SSCI, A&HCI Timespan=2000-01-01 - 2012-09-21*<br>*Lemmatization=On*
# 68	**1,248**	#59 AND #6<br>Refined by: Web of Science Categories=(MEDICINE GENERAL INTERNAL)<br>*Databases=SCI-EXPANDED, SSCI, A&HCI Timespan=2000-01-01 - 2012-09-21*<br>*Lemmatization=On*
# 67	**3,064**	#59 AND #6<br>Refined by: Web of Science Categories=(PUBLIC ENVIRONMENTAL OCCUPATIONAL HEALTH)<br>*Databases=SCI-EXPANDED, SSCI, A&HCI Timespan=2000-01-01 - 2012-09-21*<br>*Lemmatization=On*
# 66	**1,560**	#59 AND #6<br>Refined by: Web of Science Categories=(IMMUNOLOGY)<br>*Databases=SCI-EXPANDED, SSCI, A&HCI Timespan=2000-01-01 - 2012-09-21*<br>*Lemmatization=On*
# 65	**2,080**	#59 AND #6<br>Refined by: Web of Science Categories=(INFECTIOUS DISEASES)<br>*Databases=SCI-EXPANDED, SSCI, A&HCI Timespan=2000-01-01 - 2012-09-21*<br>*Lemmatization=On*
# 64	**10,555**	#59 AND #6<br>Refined by: Web of Science Categories=(PUBLIC ENVIRONMENTAL OCCUPATIONAL HEALTH OR INFECTIOUS DISEASES OR OBSTETRICS GYNECOLOGY OR IMMUNOLOGY OR MEDICINE GENERAL INTERNAL OR TROPICAL MEDICINE OR PEDIATRICS OR VIROLOGY OR MICROBIOLOGY OR SOCIAL SCIENCES BIOMEDICAL OR PARASITOLOGY OR HEALTH POLICY SERVICES OR NUTRITION DIETETICS OR NURSING OR MEDICINE RESEARCH EXPERIMENTAL OR HEALTH CARE SCIENCES SERVICES OR REPRODUCTIVE BIOLOGY)<br>*Databases=SCI-EXPANDED, SSCI, A&HCI Timespan=2000-01-01 - 2012-09-21*<br>*Lemmatization=On*
# 63	**10,852**	#59 AND #6<br>Refined by: Web of Science Categories=(PUBLIC ENVIRONMENTAL OCCUPATIONAL HEALTH OR INFECTIOUS DISEASES OR OBSTETRICS GYNECOLOGY OR IMMUNOLOGY OR WOMEN S STUDIES OR MEDICINE GENERAL INTERNAL OR TROPICAL MEDICINE OR PEDIATRICS OR VIROLOGY OR MICROBIOLOGY OR SOCIAL SCIENCES BIOMEDICAL OR PARASITOLOGY OR HEALTH POLICY SERVICES OR NUTRITION DIETETICS OR NURSING OR MEDICINE RESEARCH EXPERIMENTAL OR BIOLOGY OR RESPIRATORY SYSTEM OR HEALTH CARE SCIENCES SERVICES OR DEMOGRAPHY OR ENVIRONMENTAL SCIENCES OR PSYCHOLOGY MULTIDISCIPLINARY OR REPRODUCTIVE BIOLOGY OR SURGERY) AND Research Areas=(PUBLIC ENVIRONMENTAL OCCUPATIONAL HEALTH OR INFECTIOUS DISEASES OR OBSTETRICS GYNECOLOGY OR IMMUNOLOGY OR GENERAL INTERNAL MEDICINE OR TROPICAL MEDICINE OR PEDIATRICS OR VIROLOGY OR MICROBIOLOGY OR BIOMEDICAL SOCIAL SCIENCES OR HEALTH CARE SCIENCES SERVICES OR PARASITOLOGY OR NUTRITION DIETETICS OR NURSING OR RESEARCH EXPERIMENTAL MEDICINE OR LIFE SCIENCES BIOMEDICINE OTHER TOPICS OR REPRODUCTIVE BIOLOGY OR WOMEN S STUDIES)<br>*Databases=SCI-EXPANDED, SSCI, A&HCI Timespan=2000-01-01 - 2012-09-21*

		Lemmatization=On
# 62	**11,111**	#59 AND #6<br>Refined by: Web of Science Categories=(PUBLIC ENVIRONMENTAL OCCUPATIONAL HEALTH OR INFECTIOUS DISEASES OR OBSTETRICS GYNECOLOGY OR IMMUNOLOGY OR WOMEN S STUDIES OR MEDICINE GENERAL INTERNAL OR TROPICAL MEDICINE OR PEDIATRICS OR VIROLOGY OR MICROBIOLOGY OR SOCIAL SCIENCES BIOMEDICAL OR PARASITOLOGY OR HEALTH POLICY SERVICES OR NUTRITION DIETETICS OR NURSING OR MEDICINE RESEARCH EXPERIMENTAL OR BIOLOGY OR RESPIRATORY SYSTEM OR HEALTH CARE SCIENCES SERVICES OR DEMOGRAPHY OR ENVIRONMENTAL SCIENCES OR PSYCHOLOGY MULTIDISCIPLINARY OR REPRODUCTIVE BIOLOGY OR SURGERY)<br>*Databases=SCI-EXPANDED, SSCI, A&HCI Timespan=2000-01-01 - 2012-09-21*<br>*Lemmatization=On*
# 61	**5,217**	#59 AND #6<br>Refined by: Web of Science Categories=(OBSTETRICS GYNECOLOGY OR WOMEN S STUDIES OR MEDICINE GENERAL INTERNAL OR HEMATOLOGY OR TROPICAL MEDICINE OR PEDIATRICS OR SOCIAL SCIENCES BIOMEDICAL OR SOCIAL SCIENCES INTERDISCIPLINARY OR SOCIOLOGY OR SOCIAL ISSUES)<br>*Databases=SCI-EXPANDED, SSCI, A&HCI Timespan=2000-01-01 - 2012-09-21*<br>*Lemmatization=On*
# 60	**13,054**	#59 AND #6<br>*Databases=SCI-EXPANDED, SSCI, A&HCI Timespan=2000-01-01 - 2012-09-21*<br>*Lemmatization=On*
# 59	**95,097**	#58 OR #33 OR #29 OR #22 OR #18<br>*Databases=SCI-EXPANDED, SSCI, A&HCI Timespan=2000-01-01 - 2012-09-21*<br>*Lemmatization=On*
# 58	**37,609**	#57 OR #56 OR #55 OR #54 OR #53 OR #52 OR #51 OR #47 OR #46 OR #45 OR #44 OR #43 OR #42 OR #41 OR #40 OR #39 OR #38 OR #37 OR #36 OR #35 OR #34<br>*Databases=SCI-EXPANDED, SSCI, A&HCI Timespan=2000-01-01 - 2012-09-21*<br>*Lemmatization=On*
# 57	**395**	Topic=((*attend* childbirth*))<br>*Databases=SCI-EXPANDED, SSCI, A&HCI Timespan=2000-01-01 - 2012-09-21*<br>*Lemmatization=On*
# 56	**2,833**	Topic=((*attend* birth))<br>*Databases=SCI-EXPANDED, SSCI, A&HCI Timespan=2000-01-01 - 2012-09-21*<br>*Lemmatization=On*
# 55	**1,592**	Topic=("maternal care")<br>*Databases=SCI-EXPANDED, SSCI, A&HCI Timespan=2000-01-01 - 2012-09-21*<br>*Lemmatization=On*
# 54	**3,944**	Topic=(midwife)<br>*Databases=SCI-EXPANDED, SSCI, A&HCI Timespan=2000-01-01 - 2012-09-21*<br>*Lemmatization=On*
# 53	**529**	Topic=("birth attendant*")<br>*Databases=SCI-EXPANDED, SSCI, A&HCI Timespan=2000-01-01 - 2012-09-21*<br>*Lemmatization=On*
# 52	**587**	Topic=("place of birth")<br>*Databases=SCI-EXPANDED, SSCI, A&HCI Timespan=2000-01-01 - 2012-09-21*<br>*Lemmatization=On*
# 51	**2,632**	#13 AND #50<br>*Databases=SCI-EXPANDED, SSCI, A&HCI Timespan=2000-01-01 - 2012-09-21*<br>*Lemmatization=On*
# 50	**8,104**	#49 OR #48<br>*Databases=SCI-EXPANDED, SSCI, A&HCI Timespan=2000-01-01 - 2012-09-21*<br>*Lemmatization=On*
# 49	**8,104**	Topic=((labour management))<br>*Databases=SCI-EXPANDED, SSCI, A&HCI Timespan=2000-01-01 - 2012-09-21*<br>*Lemmatization=On*
# 48	**8,104**	Topic=((labor management))<br>*Databases=SCI-EXPANDED, SSCI, A&HCI Timespan=2000-01-01 - 2012-09-21*<br>*Lemmatization=On*
# 47	**1,002**	Topic=("obstetric care")<br>*Databases=SCI-EXPANDED, SSCI, A&HCI Timespan=2000-01-01 - 2012-09-21*<br>*Lemmatization=On*
# 46	**195**	Topic=("intrapartum care")<br>*Databases=SCI-EXPANDED, SSCI, A&HCI Timespan=2000-01-01 - 2012-09-21*<br>*Lemmatization=On*
# 45	**17,221**	Topic=(pregnan* complicat*)<br>*Databases=SCI-EXPANDED, SSCI, A&HCI Timespan=2000-01-01 - 2012-09-21*<br>*Lemmatization=On*
# 44	**7**	Topic=("complication* of labour")<br>*Databases=SCI-EXPANDED, SSCI, A&HCI Timespan=2000-01-01 - 2012-09-21*<br>*Lemmatization=On*

# 43	**32**	Topic=("complication* of labor")<br>*Databases=SCI-EXPANDED, SSCI, A&HCI Timespan=2000-01-01 - 2012-09-21*<br>*Lemmatization=On*
# 42	**2,810**	Topic=(complication* of labor)<br>*Databases=SCI-EXPANDED, SSCI, A&HCI Timespan=2000-01-01 - 2012-09-21*<br>*Lemmatization=On*
# 41	**1,755**	Topic=(dystocia)<br>*Databases=SCI-EXPANDED, SSCI, A&HCI Timespan=2000-01-01 - 2012-09-21*<br>*Lemmatization=On*
# 40	**465**	Topic=(OBSTRUCT* LABOUR)<br>*Databases=SCI-EXPANDED, SSCI, A&HCI Timespan=2000-01-01 - 2012-09-21*<br>*Lemmatization=On*
# 39	**1,223**	Topic=(PROLONG* LABOUR)<br>*Databases=SCI-EXPANDED, SSCI, A&HCI Timespan=2000-01-01 - 2012-09-21*<br>*Lemmatization=On*
# 38	**1,223**	Topic=(PROLONG* LABOR)<br>*Databases=SCI-EXPANDED, SSCI, A&HCI Timespan=2000-01-01 - 2012-09-21*<br>*Lemmatization=On*
# 37	**465**	Topic=(OBSTRUCT* LABOR)<br>*Databases=SCI-EXPANDED, SSCI, A&HCI Timespan=2000-01-01 - 2012-09-21*<br>*Lemmatization=On*
# 36	**1,476**	Topic=("SPONTANEOUS ABORTIONS")<br>*Databases=SCI-EXPANDED, SSCI, A&HCI Timespan=2000-01-01 - 2012-09-21*<br>*Lemmatization=On*
# 35	**3,363**	Topic=("SPONTANEOUS ABORTION")<br>*Databases=SCI-EXPANDED, SSCI, A&HCI Timespan=2000-01-01 - 2012-09-21*<br>*Lemmatization=On*
# 34	**4,380**	Topic=(miscarriage*) AND Topic=(pregnan*)<br>*Databases=SCI-EXPANDED, SSCI, A&HCI Timespan=2000-01-01 - 2012-09-21*<br>*Lemmatization=On*
# 33	**19,698**	#32 OR #31 OR #30<br>*Databases=SCI-EXPANDED, SSCI, A&HCI Timespan=2000-01-01 - 2012-09-21*<br>*Lemmatization=On*
# 32	**8,922**	Topic=(hypertens*) AND Topic=(pregnan*)<br>*Databases=SCI-EXPANDED, SSCI, A&HCI Timespan=2000-01-01 - 2012-09-21*<br>*Lemmatization=On*
# 31	**1,158**	Topic=(HELLP)<br>*Databases=SCI-EXPANDED, SSCI, A&HCI Timespan=2000-01-01 - 2012-09-21*<br>*Lemmatization=On*
# 30	**15,082**	Topic=(*eclampsia*)<br>*Databases=SCI-EXPANDED, SSCI, A&HCI Timespan=2000-01-01 - 2012-09-21*<br>*Lemmatization=On*
# 29	**2,959**	#28 OR #27 OR #26 OR #25 OR #24 OR #23<br>*Databases=SCI-EXPANDED, SSCI, A&HCI Timespan=2000-01-01 - 2012-09-21*<br>*Lemmatization=On*
# 28	**802**	Topic=(*natal* h?emorrhage)<br>*Databases=SCI-EXPANDED, SSCI, A&HCI Timespan=2000-01-01 - 2012-09-21*<br>*Lemmatization=On*
# 27	**479**	Topic=(obstetric h?emorrhage)<br>*Databases=SCI-EXPANDED, SSCI, A&HCI Timespan=2000-01-01 - 2012-09-21*<br>*Lemmatization=On*
# 26	**0**	Topic=(obstetric h?emorhage)<br>*Databases=SCI-EXPANDED, SSCI, A&HCI Timespan=2000-01-01 - 2012-09-21*<br>*Lemmatization=On*
# 25	**697**	Topic=(postpartum bleed*)<br>*Databases=SCI-EXPANDED, SSCI, A&HCI Timespan=2000-01-01 - 2012-09-21*<br>*Lemmatization=On*
# 24	**510**	Topic=("postpartum haemorrhage".)<br>*Databases=SCI-EXPANDED, SSCI, A&HCI Timespan=2000-01-01 - 2012-09-21*<br>*Lemmatization=On*
# 23	**1,353**	Topic=("postpartum hemorrhage".)<br>*Databases=SCI-EXPANDED, SSCI, A&HCI Timespan=2000-01-01 - 2012-09-21*<br>*Lemmatization=On*
# 22	**1,835**	#21 OR #19<br>*Databases=SCI-EXPANDED, SSCI, A&HCI Timespan=2000-01-01 - 2012-09-21*<br>*Lemmatization=On*
# 21	**1,835**	#13 AND #20

		Databases=SCI-EXPANDED, SSCI, A&HCI Timespan=2000-01-01 - 2012-09-21<br>*Lemmatization=On*
# 20	**18,960**	Topic=(an?emi* OR h?emoglobin)<br>*Databases=SCI-EXPANDED, SSCI, A&HCI Timespan=2000-01-01 - 2012-09-21*<br>*Lemmatization=On*
# 19	**113**	Topic=("maternal an?emia")<br>*Databases=SCI-EXPANDED, SSCI, A&HCI Timespan=2000-01-01 - 2012-09-21*<br>*Lemmatization=On*
# 18	**45,324**	#17 OR #16 OR #15 OR #12 OR #10 OR #9 OR #8 OR #7<br>*Databases=SCI-EXPANDED, SSCI, A&HCI Timespan=2000-01-01 - 2012-09-21*<br>*Lemmatization=On*
# 17	**43**	Topic=("PUERPERAL INFECTION")<br>*Databases=SCI-EXPANDED, SSCI, A&HCI Timespan=2000-01-01 - 2012-09-21*<br>*Lemmatization=On*
# 16	**246**	Topic=("infection in pregnancy")<br>*Databases=SCI-EXPANDED, SSCI, A&HCI Timespan=2000-01-01 - 2012-09-21*<br>*Lemmatization=On*
# 15	**43,468**	#14 AND #13<br>*Databases=SCI-EXPANDED, SSCI, A&HCI Timespan=2000-01-01 - 2012-09-21*<br>*Lemmatization=On*
# 14	**819,451**	Topic=(infect* OR sepsis OR septic OR tubercul* OR pneumonia OR meningitis OR HIV)<br>*Databases=SCI-EXPANDED, SSCI, A&HCI Timespan=2000-01-01 - 2012-09-21*<br>*Lemmatization=On*
# 13	**417,789**	Topic=(pregnan* OR maternal OR obstetric* OR puerper* OR partum OR birth OR childbirth OR prenatal OR postnatal OR *natal*)<br>*Databases=SCI-EXPANDED, SSCI, A&HCI Timespan=2000-01-01 - 2012-09-21*<br>*Lemmatization=On*
# 12	**925**	Topic=("INTRAUTERINE INFECTION")<br>*Databases=SCI-EXPANDED, SSCI, A&HCI Timespan=2000-01-01 - 2012-09-21*<br>*Lemmatization=On*
# 11	**2,514**	Topic=(INTRAUTERINE INFECTION)<br>*Databases=SCI-EXPANDED, SSCI, A&HCI Timespan=2000-01-01 - 2012-09-21*<br>*Lemmatization=On*
# 10	**1,013**	Topic=(FEMALE GENITAL TRACT INFECTION)<br>*Databases=SCI-EXPANDED, SSCI, A&HCI Timespan=2000-01-01 - 2012-09-21*<br>*Lemmatization=On*
# 9	**155**	Topic=(FEMALE GENITAL TRACT INFLAMMATION)<br>*Databases=SCI-EXPANDED, SSCI, A&HCI Timespan=2000-01-01 - 2012-09-21*<br>*Lemmatization=On*
# 8	**1,821**	Topic=(chorioamnionitis)<br>*Databases=SCI-EXPANDED, SSCI, A&HCI Timespan=2000-01-01 - 2012-09-21*<br>*Lemmatization=On*
# 7	**7,405**	Topic=(maternal infection*)<br>*Databases=SCI-EXPANDED, SSCI, A&HCI Timespan=2000-01-01 - 2012-09-21*<br>*Lemmatization=On*
# 6	**848,085**	#5<br>*Databases=SCI-EXPANDED, SSCI, A&HCI Timespan=2000-01-01 - 2012-09-21*<br>*Lemmatization=On*
# 5	**1,375,550**	#4 OR #3 OR #2 OR #1<br>*Databases=SCI-EXPANDED, SSCI, A&HCI Timespan=All Years*<br>*Lemmatization=On*
# 4	**217,559**	Topic=(Africa OR *sahara* OR "low income country" OR "low income countries" OR "middle income country" OR "middle income countries" OR "south america" OR "central america" OR "latin america" OR carribean)<br>*Databases=SCI-EXPANDED, SSCI, A&HCI Timespan=All Years*<br>*Lemmatization=On*
# 3	**264,996**	Topic=(Afghanistan OR Bangladesh OR Benin OR Burkina Faso OR Burundi OR Cambodia OR Central African Republic OR Chad OR Comoros OR Congo, Dem. Rep OR Eritrea OR Ethiopia OR Gambia, The OR Guinea OR Guinea-Bisau OR Haiti OR Kenya OR Korea, Dem Rep OR Kyrgyz Republic OR Liberia OR Madagascar OR Malawi OR Mali OR Mozambique OR Myanmar OR Nepal OR Niger OR Rwanda OR Sierra Leone OR Somalia OR Tajikistan OR Tanzania OR Togo OR Uganda OR Zimbabwe)<br>*Databases=SCI-EXPANDED, SSCI, A&HCI Timespan=All Years*<br>*Lemmatization=On*
# 2	**380,742**	Topic=(Angola OR Armenia OR Belize OR Bhutan OR Bolivia OR Cameroon OR Cape Verde OR Congo, Rep OR C??te d'Ivoire OR Djibouti OR Egypt OR El Salvador OR Fiji OR Georgia OR Ghana OR Guatemala OR Guyana OR Honduras OR Indonesia OR India OR Iraq OR Kiribati OR Kosovo OR Lao PDR OR Lesotho OR Marshall Islands OR Mauritania OR Micronesia OR Moldova OR Mongolia OR Morocco OR Nicaragua OR Nigeria OR Pakistan OR Papua New Guinea OR Paraguay OR Philippines OR Samoa OR S??o Tom?? and Principe OR Senegal OR Solomon Islands OR Sri Lanka OR Sudan OR Swaziland OR Syria* OR Timor-Leste OR Tonga OR Turkmenistan OR Tuvalu OR Ukraine OR Uzbekistan OR Vanuatu OR Vietnam OR Gaza OR Yemen OR Zambia)<br>*Databases=SCI-EXPANDED, SSCI, A&HCI Timespan=All Years*<br>*Lemmatization=On*

# 1	**732,634**	Topic=(Albania OR Algeria OR Samoa OR Antigua OR Barbuda OR Argentina OR Azerbaijan OR Belarus OR Bosnia OR Herzegovina OR Botswana OR Brazil OR Bulgaria OR Chile OR China OR Colombia OR Costa Rica OR Cuba OR Dominica OR Dominican Republic OR Ecuador OR Gabon OR Grenada OR Iran OR Jamaica OR Jordan OR Kazakhstan OR Latvia OR Lebanon OR Libya OR Lithuania OR Macedonia OR Malaysia OR Maldives OR Mauritius OR Mayotte OR Mexico OR Montenegro OR Namibia OR Palau OR Panama OR Peru OR Romania OR Russian Federation OR SerbiaOR Seychelles OR South Africa OR St. Kitts and Nevis OR St. Lucia OR St. Vincent OR Grenadines OR Suriname OR Thailand OR Tunisia OR Turkey OR Uruguay OR Venezuela)<br>*Databases=SCI-EXPANDED, SSCI, A&HCI Timespan=All Years*<br>*Lemmatization=On*

.

Popline search strategy

This database does not allow complicated searching. Searches are limited to 1 line with limited Boolean options. The subject search option was therefore used as this has a range of maternal and child health options . Phrases and subjects are listed below

1 Subject pregnancy and childbirth complications
2. Safe motherhood
3. "postpartum hemorrhage"
4. Antenatal care –
5. Post-partum care – post partum women
6. Maternal care (limits -– developing countries – health services)
7. Maternal care (limits– developing countries – delivery of health services)
8. Maternal care (limits treatment – developing countries)
9. Maternal care (limits evaluation – developing countries)
10. Maternal mortality (limits developing countries)
11. Post-partum care
12. Contraception for post-partum women
13. Early detection (limits developing countries)
14. Danger signs (limits developing countries and socioeconomic factors)

LILACS

A combination of search terms were used all of which were limited to items about pregnancy. Search terms were: anemia or anaemia, birth attendant, hemorrhage or haemorrhage, infections terms, intrauterine infection, intrapartum care, maternal infection, maternal mortality, miscarriage, pre-eclampsia.

Other sources

Not all databases and registers of research allow the user to use sophisticated approaches for searching. Some may not use index terms, and incorporate limited options for combining terms and search strings. They may also be limited in the manner in which search results can be saved, if at all. It is therefore more difficult to record search strategies and results of screening for these sources

Annex 3. Study resources

Equity extension of PRISMA guidelines

Website references

EPPI-Reviewer 4 web based systematic review software
http://eppi.ioe.ac.uk/cms/Default.aspx?alias=eppi.ioe.ac.uk/cms/er4

EPPI-Centre Health Promotion and Public Health Reviews Facility
http://eppi.ioe.ac.uk/cms/Default.aspx?tabid=73

EPPI Centre website and list of all systematic reviews:
http://eppi.ioe.ac.uk/cms/Default.aspx?tabid=62

EPPI Centre Teaching and Learning

http://eppi.ioe.ac.uk/cms/Default.aspx?tabid=168

Publications

Methods for the thematic synthesis of qualitative research in systematic reviews
http://www.biomedcentral.com/1471-2288/8/45

BMJ paper example of equity review – healthy eating mixed methods
http://es.scribd.com/doc/40588135/Integrating-Qualitative-Research-with-Trials-in-systematic-reviews

Chapter on mixed methods synthesis
https://portal.ioe.ac.uk/http/onlinelibrary.wiley.com/doi/10.1002/9781119959847.ch6/summary

Map of inequalities in young people's health example of equity review
http://eppi.ioe.ac.uk/cms/LinkClick.aspx?fileticket=mVu6mYHcwbc%3d&tabid=2410&mid=4471

Reflections on developing and using PROGRESS-Plus

http://equity.cochrane.org/Files/Equity_Update_Vol2_Issue1.pdf

Annex 4. List of low- and middle-income countries

http://data.worldbank.org/about/country-classifications/country-and-lending-groups (6)

East Asia and Pacific		
American Samoa	Malaysia	Samoa
Cambodia	Marshall Islands	Solomon Islands
China	Micronesia, Fed. Sts	Thailand
Fiji	Mongolia	Timor-Leste
Indonesia	Myanmar	Tuvalu
Kiribati	Palau	Tonga
Korea, Dem. Rep.	Papua New Guinea	Vanuatu
Lao PDR	Philippines	Vietnam
**Europe and Central Asia**		
Albania	Kosovo	Russian Federation
Armenia	Kyrgyz Republic	Serbia
Azerbaijan	Latvia	Tajikistan
Belarus	Lithuania	Turkey
Bosnia and Herzegovina	Macedonia, FYR	Turkmenistan
Bulgaria	Moldova	Ukraine
Georgia	Montenegro	Uzbekistan
Kazakhstan	Romania	
**Latin America and the Caribbean**		
Antigua and Barbuda	Dominican Republic	Nicaragua
Argentina	Ecuador	Panama
Belize	El Salvador	Paraguay
Bolivia	Grenada	Peru
Brazil	Guatemala	St. Kitts and Nevis
Chile	Guyana	St. Lucia
Colombia	Haiti	St. Vincent and the Grenadines
Costa Rica	Honduras	Suriname
Cuba	Jamaica	Uruguay
Dominica	Mexico	Venezuela, RB
**Middle East and North Africa**		
Algeria	Jordan	Tunisia
Djibouti	Lebanon	West Bank and Gaza
Egypt, Arab Rep.	Libya	Yemen, Rep.
Iran, Islamic Rep.	Morocco	
Iraq	Syrian Arab Republic	
**South Asia**		
Afghanistan	India	Pakistan
Bangladesh	Maldives	Sri Lanka
Bhutan	Nepal	
**Sub-Saharan Africa**		
Angola	Gambia, The	Nigeria
Benin	Ghana	Rwanda
Botswana	Guinea	São Tomé and Principe
Burkina Faso	Guinea-Bissau	Senegal
Burundi	Kenya	Seychelles
Cameroon	Lesotho	Sierra Leone
Cape Verde	Liberia	Somalia
Central African Republic	Madagascar	South Africa
Chad	Malawi	South Sudan
Comoros	Mali	Sudan
Congo, Dem. Rep.	Mauritania	Swaziland

Congo, Rep	Mauritius	Tanzania
Côte d'Ivoire	Mayotte	Togo
Eritrea	Mozambique	Uganda
Ethiopia	Namibia	Zambia
Gabon	Niger	Zimbabwe

Low-income economies ($1,005 or less)

Afghanistan	Gambia, The	Myanmar
Bangladesh	Guinea	Nepal
Benin	Guinea-Bisau	Niger
Burkina Faso	Haiti	Rwanda
Burundi	Kenya	Sierra Leone
Cambodia	Korea, Dem Rep.	Somalia
Central African Republic	Kyrgyz Republic	Tajikistan
Chad	Liberia	Tanzania
Comoros	Madagascar	Togo
Congo, Dem. Rep	Malawi	Uganda
Eritrea	Mali	Zimbabwe
Ethiopia	Mozambique	

Lower-middle-income economies ($1,006 to $3,975)

Angola	India	São Tomé and Principe
Armenia	Iraq	Senegal
Belize	Kiribati	Solomon Islands
Bhutan	Kosovo	Sri Lanka
Bolivia	Lao PDR	Sudan
Cameroon	Lesotho	Swaziland
Cape Verde	Marshall Islands	Syrian Arab Republic
Congo, Rep.	Mauritania	Timor-Leste
Côte d'Ivoire	Micronesia, Fed. Sts.	Tonga
Djibouti	Moldova	Turkmenistan
Egypt, Arab Rep.	Mongolia	Tuvalu
El Salvador	Morocco	Ukraine
Fiji	Nicaragua	Uzbekistan
Georgia	Nigeria	Vanuatu
Ghana	Pakistan	Vietnam
Guatemala	Papua New Guinea	West Bank and Gaza
Guyana	Paraguay	Yemen, Rep.
Honduras	Philippines	Zambia
Indonesia	Samoa	

Upper-middle-income economies ($3,976 to $12,275)

Albania	Ecuador	Namibia
Algeria	Gabon	Palau
American Samoa	Grenada	Panama
Antigua and Barbuda	Iran, Islamic Rep.	Peru
Argentina	Jamaica	Romania
Azerbaijan	Jordan	Russian Federation
Belarus	Kazakhstan	Serbia
Bosnia and Herzegovina	Latvia	Seychelles
Botswana	Lebanon	South Africa
Brazil	Libya	St. Kitts and Nevis
Bulgaria	Lithuania	St. Lucia
Chile	Macedonia, FYR	St. Vincent and the Grenadines
China	Malaysia	Suriname
Colombia	Maldives	Thailand
Costa Rica	Mauritius	Tunisia
Cuba	Mayotte	Turkey
Dominica	Mexico	Uruguay

Dominican Republic	Montenegro	Venezuela, RB

Annex 5. List of key coding examples

Screening on title and abstract

Not maternal health

Impact of comorbidities on time in therapeutic range in patients with nonvalvular atrial fibrillation

2012

Choi J C; Damaraju C ; Mills R M; Wildgoose P ; Fields L ; Schein J ; Nelson W W;

OBJECTIVES: Time in therapeutic range (TTR) may be a quality indicator for anticoagulation. Previous studies have demonstrated that heart failure (HF) and other comorbidities are associated with poorer anticoagulation control; however, this association was not studied in a representative US population. The objective was to determine the association between HF, other comorbidities, patient characteristics, and TTR among patients with nonvalvular atrial fibrillation (NVAF). METHODS: We analyzed longitudinal patient-level anticoagulation management records collected between 2006 and 2010 by decision support software, Coag- Clinic. Adult patients with NVAF who used warfarin over 12 months with no gap >60 days between visits were identified. The Rosendaal method was used to cal- culate TTR, and TTR <55% was defined as "lower TTR". CHADS2>=2 was defined as "higher CHADS2". Logistic regression analyses were conducted to determine the association between comorbidities and TTR. RESULTS: We identified 23,425 patients. The mean (+/-SD) age was 74.8+/-9.7 years, with 84.8% >=65 years. The most common comorbidities were hypertension (41.7%), diabetes (24.1%), HF (11.7%), and stroke (11.1%). The mean (+/-SD) TTR was 67.3+/-14.4; 18.7% of patients had "lower TTR". In multivariable analyses, using age, gender, hypertension, diabetes, stroke, and region as covariates, HF was associated with "lower TTR" [adjusted OR (95%CI) = 1.41 (1.28, 1.56); p<.001]. Diabetes [1.28 (1.19, 1.38); p<.001], and stroke [1.15 (1.04, 1.27); p<.001] were also associated with "lower TTR". In the second multivariable analyses, using gender, and region as covariates, "higher CHADS2" was associated with "lower TTR" [adjusted OR (95%CI) = 1.11 (1.04, 1.18); p<.001]. CONCLUSIONS: Common comorbidities that accompany NVAF are associated with "lower TTR". HF was associated with the greatest likelihood of a "lower TTR", followed by diabetes, then stroke. Anticoagulation control is more challenging for patients with these conditions. Novel agents offering a predictable dose-response may benefit these patients.

Not maternal health

Implementation of computerized provider order entry in a neonatal intensive care unit: Impact on admission workflow

2012

Chapman A K; Lehmann C U; Donohue P K; Aucott S W;

Objective: The study objective was to determine if computerized provider order entry (CPOE) systems impaired or enhanced workflow in the neonatal intensive care unit (NICU) by comparing the timing of administration of the first dose of antibiotics before and after CPOE system implementation. Methods: We conducted a pre-post intervention comparative study of the length of time between admission and administration of initial antibiotics in neonates before and after a CPOE system was implemented. Clinical information and timing of antibiotic administration were collected on all inborn infants, who were admitted to the NICU in the first 4. h of life and treated with antibiotics, for one year prior to the implementation of computerized order entry and for one year after the implementation. Results: Infants admitted to the NICU were similar in both periods (mean birth weight 2183. g vs. 2091. g, gestational age 33.3 weeks vs. 33.0 weeks). There was no significant difference in mean length of time from admission to antibiotic administration in the pre-CPOE group (131. min [CI 124-139]) compared to the post-CPOE group (125. min [CI 116-133]) (p=0.07). The mean time to pharmacy verification for a subset of patients was significantly shorter for patients in the post-CPOE group (61 +/- 58. min) compared to the pre-CPOE group (88 +/- 76. min) (p=<0.001). Conclusions: While the introduction of a CPOE system in the NICU did not significantly improve antibiotic administration times, the timeliness of an important aspect of the medication process, time to pharmacy verification, was improved. These findings imply other factors are impeding workflow. Further studies are needed to evaluate how CPOE systems combined with patient care activities affect workflow and overall patient care. 2011 Elsevier Ireland Ltd.

Not maternal health

Childhood attention-deficit/hyperactivity disorder and future substance use disorders: Comparative meta-analyses

2011

Charach A ; Yeung E ; Climans T ; Lillie E ;

Objective In recent years cohort studies have examined childhood attention-deficit/hyperactivity disorder (ADHD) as a risk factor for substance use disorders (SUDs) in adolescence and young adulthood. The long-term risk is estimated for development of alcohol, cannabis, combined alcohol and psychoactive SUDs, combined SUDs (nonalcohol), and nicotine use disorders in children with ADHD. Method MEDLINE, CINHAL, PsycINFO, and EMBASE were searched through October 2009; reference lists of included studies were hand-searched. Prospective cohort studies were included if they compared children with ADHD to children without, identified cases using standardized criteria by mean age of 12 years, followed participants until adolescence (nicotine use) or young adulthood (psychoactive substance use disorder, with and without alcohol, alcohol use disorder, cannabis use disorder), and reported SUD outcomes. Two

independent reviewers examined articles and extracted and cross-checked data. Effects were summarized as pooled odds ratios (ORs) in a random effects model. Results Thirteen studies were included. Only two of five meta-analyses, for alcohol use disorder (N = 3,184) and for nicotine use (N = 2,067), estimated ORs showing stability when evaluated by sensitivity analyses. Childhood ADHD was associated with alcohol use disorder by young adulthood (OR = 1.35, 95% confidence interval = 1.11-1.64) and with nicotine use by middle adolescence (OR = 2.36, 95% confidence interval = 1.71-3.27). The association with drug use disorder, nonalcohol (N = 593), was highly influenced by a single study. Conclusions Childhood ADHD is associated with alcohol and drug use disorders in adulthood and with nicotine use in adolescence. 2011 American Academy of Child and Adolescent Psychiatry.

Single Clinical Intervention

Management of a pregnant patient with Graves' disease complicated by propylthiouracil induced agranulocytosis.

2005

Cho YY ; Shon HS ; Yoon HD ;

Relapse and exacerbation of Graves' disease during pregnancy is rare, and thionamide induced agranulocytosis is an uncommon side effect. We report a case of a pregnant woman in her 24th week of gestation that experienced a relapse of Graves' disease that was complicated by propylthiouracil induced agranulocytosis. Following the discontinuation of propylthiouracil and administration of a broad-spectrum of antibiotics, agranulocytosis subsided within 10 days. A total thyroidectomy to avoid any future relapse was planned and a short course of a beta-adrenergic blocker and Lugol solution were prescribed before the operation. At the 28th week of gestation, a total thyroidectomy was performed without complications and thyroxine replacement therapy was commenced. At the 40th week of gestation, labor was induced and a 3,370 g healthy male infant was born without clinical features of thyrotoxicosis. We report herein on the patient and the treatment options for this rare and complicated case.

Single Clinical Intervention

Laparoscopic cornuotomy using a temporary tourniquet suture and diluted vasopressin injection in interstitial pregnancy.

Choi YS ; Eun DS ; Choi J ; Shin KS ; Choi JH ; Park HD ;

2009

OBJECTIVE: To evaluate the efficiency of laparoscopic cornuotomy. DESIGN: Retrospective case review. SETTING: An urban medical center. PATIENT(S): Eight patients with interstitial pregnancy who have undergone laparoscopic cornuotomy. INTERVENTION(S): Laparoscopic cornuotomy was performed using a temporary tourniquet suture and the injection of diluted vasopressin around the cornual mass. The tourniquet suture was removed completely after repairing the cornu. MAIN OUTCOME MEASURE(S): Operating time, hemorrhage, beta-hCG levels. RESULT(S): The estimated blood loss was 50 +/- 22 mL (mean +/- SD), and the operating time was 58 +/- 16 minutes. The serum beta-hCG level returned to within the normal range approximately 4 weeks postoperatively in all patients. There were no major postoperative complications, such as hemorrhage, and no postoperative adjuvant therapy was required. CONCLUSION(S): Laparoscopic cornuotomy is a safe and effective method in interstitial pregnancy, and we believe that it has the advantage of preserving reproductive capacity over cornual resection.

Single clinical intervention

Labor induction at term: a comparison of the effects of 50 microg and 25 microg vaginal misoprostol.

2007

Eroglu D ; Oktem M ; Yanik F ; Kuscu E ;

PURPOSE OF INVESTIGATION: To compare the effects of 50 microg of vaginal misoprostol with 25 microg for labor induction at term. METHODS: One hundred and forty-seven pregnant women with indications for labor induction and cervical Bishop's score of < or = 6 were randomly assigned to receive either 50 microg (n = 74) or 25 microg (n = 73) of vaginal misoprostol every four hours until either a Bishop's score of > or = 8 or adequate uterine contraction frequency had been achieved. Induction-to-vaginal-delivery time was considered the primary outcome measure. RESULTS: Mean induction-to-vaginal-delivery time was significantly shorter in the 50-microg group than in the 25-microg group (526 +/- 141 min vs 745 +/- 218 min, respectively); oxytocin was administered to 65.8% of the patients in the 25-microg group and to 35.1% in the 50-microg group (p < .05). The incidence of tachysystole was significantly higher in the 50-microg group than in the 25-microg group (12% vs 2.7%, p < .05). We found no statistically significant difference between the two groups with respect to the rate of primary cesarean section, incidence of hyperstimulation syndrome, or neonatal outcome (p > .05). CONCLUSION: Fifty micrograms of vaginally administered misoprostol is an effective and inexpensive means of inducing labor at term. Uterine tachysystole may be associated more frequently with a 50-microg dose of vaginal misoprostol than with a 25-microg dose. Clinicians must accurately document the frequency and intensity of uterine contractions before every 50-microg dose of misoprostol is administered.

Single clinical intervention or no intervention

Laparoscopic management of a primary omental pregnancy after clomiphene induction.

2009

Esin S ; Yildirim H ; Tanzer F

OBJECTIVE: To describe the successful laparoscopic management of a primary omental pregnancy. DESIGN: Case report. SETTING: Department of Obstetrics and Gynecology, Dr. Sami Ulus Obstetrics, Gynecology and Children's Hospital, Ankara, Turkey. PATIENT(S): A 22-year-old patient with an omental pregnancy. INTERVENTION(S): Laparoscopic partial omentectomy. MAIN OUTCOME MEASURE(S):

Successful laparoscopic management of an omental pregnancy. RESULT(S): A 22-year-old woman presented to the emergency room with abdominal pain and vaginal spotting. She was undergoing clomiphene (CC) induction for infertility and had a positive urine pregnancy test at home. A right adnexal ectopic pregnancy was reported by ultrasonography. Due to increasing pain, laparoscopy was performed. The uterus and fallopian tubes appeared normal without any signs of pregnancy. A well-vascularized intact omental gestational sac was discovered in the right adnexal region in close proximity to the right ovary. By laparoscopy, the sac was resected with partial omentectomy. A primary omental pregnancy was confirmed by beta-hCG-positive trophoblast cells among omental fat cells. CONCLUSION(S): Omental pregnancy is rather difficult to identify due to localization. When in close proximity to the adnexal region, it may mimic a tubal ectopic pregnancy. Laparoscopy offers a minimally invasive method for diagnosis and therapy.

No Intervention/Outcome

Barriers to utilization of prenatal care services in Turkey.

2003

Erci B ;

PURPOSE: To identify barriers to utilization of prenatal care services in Turkey, including pregnant women's attitudes toward pregnancy and prenatal care. DESIGN: Descriptive. The population was Turkish women who lived in Erzurum and had delivered their infants but were still hospitalised. METHODS: The sample of 446 women had or had not received prenatal care, had no complications during pregnancy, carried their pregnancies to term, and were considered to have normal deliveries. Attitudes toward pregnancy and prenatal care and barriers to prenatal care services were measured by use of a questionnaire. FINDINGS: Low education of pregnant women and unwanted pregnancy were barriers to use of prenatal care services. Additional barriers were negative attitudes toward pregnancy and attitudes toward prenatal care. These barriers decreased frequency of use and delayed early initiation of prenatal care. The most important barrier reported by the women was being too busy at home to seek care. CONCLUSIONS: Although this sample was limited, the findings indicate barriers for attention by health care providers to ensure appropriate prenatal care and maternal and infant health.

No Intervention or outcome

A case of Mallory-Weiss syndrome complicating pregnancy in a patient with scleroderma.

2003

Cho KH ; Heo SW ; Chung SH ; Kim CG ; Kim HG ; Choe JY ;

The majority of patients with scleroderma have gastrointestinal involvement, and a few experience gastrointestinal hemorrhage, however, gastrointestinal hemorrhage due to Mallory-Weiss syndrome is very rare. We report upon a 24-year-old pregnant woman with scleroderma who had gastrointestinal hemorrhage due to Mallory-Weiss syndrome.

No Intervention or outcome

Obesity and periodontal disease in diabetic pregnant women.

2005

Chapper A ; Munch A ; Schermann C ; Piacentini CC ; Fasolo MT ;

This cross-sectional study investigated the impact of pregestational overweight and obesity on periodontal status of patients with gestational diabetes mellitus (GDM). Sixty pregnant women with gestational diabetes mellitus (GDM) were recruited for the study. According to the pregestational body mass index (BMI), patients were classified into 3 groups: normal, overweight or obese. The periodontal assessment parameters were the presence of gingival bleeding (GB) and bleeding on probing (BOP) per tooth. Clinical attachment loss (CAL) was assessed per tooth and classified according to following values: 1) absence of attachment loss; 2) between 1 and 2 mm, 3) between 3 and 5 mm; and 4) CAL > or = 6 mm. The means of individual percentage of teeth with GB and BOP and the means of the individual classified values of CAL were compared through ANOVA. Differences between the groups were established through post hoc Bonferroni test for multiple comparisons ($p < 0.05$). The analysis revealed significant differences between the normal group and the obese group considering GB (52.76% +/- 27.99% and 78.85% +/- 27.44%, respectively) and CAL (2.21 +/- 0.41 and 2.61 +/- 0.54, respectively). Although an increase was found in BOP as the BMI increased (ranging from 55.65% to 75.31%), no statistically significant differences were found among the groups. Patients with GDM and pregestational obesity had significantly more gingivitis and periodontal attachment loss that those with normal pregestational BMI. Periodontal treatment should be considered in the establishment of future recommendations for metabolic control for this special group of patients.

No intervention or outcome (If this was about overall service use then could be coded as service utilisation)

MEN IN MATERNAL CARE: EVIDENCE FROM INDIA

2012

Chattopadhyay A ;

Men's supportive stance is an essential component for making women's world better. There are growing debates among policymakers and researchers on the role of males in maternal health programmes, which is a big challenge in India where society is male driven. This study aims to look into the variations and determinants of maternal health care utilization in India and in three demographically and socioeconomically disparate states, namely Uttar Pradesh, West Bengal and Maharashtra, by husband's knowledge, attitude, behaviour towards maternal health care and gender violence, using data from the National Family Health Survey III 2005-06 (equivalent to the Demographic and Health Survey in India). Women's antenatal care visits, institutional delivery and freedom in health care decisions are

looked into, by applying descriptive statistics and multivariate models. Men's knowledge about pregnancy-related care and a positive gender attitude enhances maternal health care utilization and women's decision-making about their health care, while their presence during antenatal care visits markedly increases the chances of women's delivery in institutions. From a policy perspective, proper dissemination of knowledge about maternal health care among husbands and making the husband's presence obligatory during antenatal care visits will help primary health care units secure better male involvement in maternal health care.

Health Systems
+ Other evaluation design
Determinants of reduction in maternal mortality in Matlab, Bangladesh: a 30-year cohort study.
No year given
Chowdhury ME ; Botlero R ; Koblinsky M ; Saha SK ; Dieltiens G ; Ronsmans C ;
BACKGROUND: Research on the effectiveness of strategies to reduce maternal mortality is scarce. We aimed to assess the contribution of intervention strategies, such as skilled attendance at birth, to the recorded reduction in maternal mortality in Matlab, Bangladesh. We examined and compared trends in maternal mortality in two adjacent areas over 30 years, by separate analyses of causes of death, underlying sociodemographic determinants, and areas and time periods in which interventions differed. METHODS: We analysed survey data that was routinely collected between 1976 and 2005 for about 200 000 inhabitants of Matlab, in Bangladesh, in adjacent areas served by either the International Centre for Diarrhoeal Disease Research, Bangladesh (ICDDR,B) or by the government. We used logistic regression to assess time trends in maternal mortality. We separately analysed deaths due to direct obstetric causes, abortion-related causes, and other causes. FINDINGS: Maternal mortality fell by 68% in the ICDDR,B service area and by 54% in the government service area over 30 years. Maternal mortality remained stable between 1976 and 1989 (crude annual OR 1.00 [0.98-1.01]) but decreased substantially after 1989 (OR 0.95 [0.93-0.97]). The speed of decline was faster after the skilled-attendance strategy was introduced in the ICDDR,B service area in 1990 (p=0.09). Abortion-related mortality fell sharply from 1990 onwards (OR 0.91 [0.86-0.95]). Educational differentials for mortality were substantial; the OR for more than 8 years of schooling compared with no schooling was 0.30 (0.21-0.44) for maternal mortality and 0.09 (0.02-0.37) for abortion mortality. INTERPRETATION: The fall in maternal mortality over 30 years occurred despite a low uptake of skilled attendance at birth. Part of the decline was due to a fall in abortion-related deaths and better access to emergency obstetric care; midwives might also have contributed by facilitating access to emergency care. Investment in midwives, emergency obstetric care, and safe pregnancy termination by manual vacuum aspiration have clearly been important. However, additional policies, such as those that bring about expansion of female education, better financial access for the poor, and poverty reduction, are essential to sustain the successes achieved to date.

Health systems
+other evaluation design
Postpartum care survey results from Sub-Saharan Africa.
2008
Charurat E ; Nash-Mercado A ;
This report assembles survey results conducted between March and June of 2008 to identify, document, and share information on the status of postpartum care services implemented through USAID and our partners. The survey results indicate a number of opportunities to integrate postpartum family planning with many programs. A total of 37 projects in sub-Saharan Africa responded to the survey; most were working in family planning, HIV/AIDS, child survival/child health and maternal and newborn health. Training, service delivery, behavior change communication and community approaches were the main intervention areas of the projects surveyed. Since most of the projects work with women of reproductive age and children under five years, there are opportunities to integrate postpartum family planning (PPFP) with routine immunization, well-child and sick-child visits. Opportunities to include postpartum family planning (PPFP) in trainings also exist in a number of the projects. Survey results indicated that there are a number of opportunities to integrate postpartum family planning (PPFP) with many programs. Recommendations include: 1) Utilize community-based volunteers in PPFP interventions; 2) Emphasize the Lactational Amenorrhea Method (LAM) as a transition method; and 3) Advocate for policies that effectively promote PPFP.

Health Systems
+ Impact evaluation
Potential for reducing child and maternal mortality through reproductive and child health intervention programmes: an illustrative case study from India.
2006
Choe MK ; Chen J ;
In this paper, the authors first examine patterns of major correlates of under-five mortality rate and maternal mortality ratios, as well as the progress towards meeting the Goals of reducing under-five mortality rate and maternal mortality ratio among the countries in the Asian and Pacific region. Doing so, one hopes to get a better understanding of why some countries are progressing well towards meeting some of the Goals while some are lagging behind. It is followed by an in-depth analysis of estimating potential for reducing under-five mortality through reproductive and child health intervention programmes including family planning, antenatal care and child immunization, using India as an illustrative example. (excerpt)

Maternal HIV/STIs
+ Impact Evaluation
Changes in vertically transmitted human immunodeficiency virus infection Chile
2007
Chávez P ; Ana ; Alvarez P ; Ana M ; Wu H ; Elba ; Peña D ; Anamaría ; Vizueta R ; Eloísa ;
La identificación de diversos factores que inciden en el riesgo de la transmisión madre-hijo del virus de inmunodeficiencia humana (VIH), permitió diseñar estrategias dirigidas a disminuir su transmisión, entre ellas, medidas destinadas a disminuir la carga viral de la madre, disminuir la exposición del niño al VIH durante el parto y eliminar la exposición al mismo a través de la leche materna. Destaca la administración de anti-retrovirales durante el embarazo, parto y en el recién nacido, inicialmente, como protocolo PACTG 076 que utilizaba zidovudina y, posteriormente, el uso de trite-rapia. De esta manera, en las madres incorporadas en protocolos de profilaxis de transmisión vertical (TV) del VIH se logró reducir la transmisión de este virus, inicialmente, a 9,5 por ciento y en la última evaluación, realizada entre 1998 y julio 2005, a 2 por ciento. Sin embargo, han continuado naciendo niños infectados hijos de madres en las que no se conocía su condición serológica, lo que reafirma que la medida fundamental para disminuir los casos de infección por VIH en niños, es la pesquisa universal de la infección en las mujeres embarazadas, de manera que accedan en forma oportuna a protocolos de profilaxis, lo que se espera lograr con la nueva norma de prevención de TV del VIH, promulgada en agosto de 2005, por la Comisión Nacional del SIDA del Ministerio de Salud.(AU) The identification of various risk factors of vertical human immunodeficiency virus (HIV) transmission resulted in the development of strategies whose aim was to decrease the mother's viral load, to reduce her child's exposure to it during delivery, and to avoid the subsequent viral exposure due to breastfeeding. The administration of antiretroviral treatment during pregnancy, delivery and to the neonate (PACTG 076) proved to be useful. At a first stage, zidovudine was used. A triple combination therapy was then administered. Initially, the viral transmission in mothers who were enrolled in protocols for vertically transmitted HIV prophylaxis was reduced to 9.5 percent, whereas the last measurement carried out between 1998 and 2005, the initial figure was brought down to 2 percent. Nevertheless, the delivery of infected children whose mother's HIV status was unknown is still considered likely to happen. The main step to be taken to reduce HIV infection among children is to perform universal HIV tests during pregnancy, so that HIV positive pregnant patients conveniently receive proper prophylaxis. We look forward to achieving this by following the new prevention guidelines of vertically-transmitted HIV infection, developed by the Comisión Nacional del SIDA of the Chilean Health Ministry.(AU)

Maternal HIV/STIs
+ Other design
Comparison of mother-to-child transmission rates in Ugandan women with subtype A versus D HIV-1 who received single-dose nevirapine prophylaxis: HIV Network For Prevention Trials 012.
2005
Eshleman SH ; Guay LA ; Mwatha A ; Brown E ; Musoke P ; Mmiro F ; Jackson JB ;
OBJECTIVE: To compare the rate of mother-to-child transmission (MTCT) in women with subtype A versus D HIV-1 who received single-dose nevirapine (NVP). METHODS: The MTCT rates were compared in women with subtype A versus D at birth and at 8 weeks and 18 months of age of the infants. The rate of late MTCT (after 8 weeks of age) was also analyzed. RESULTS: HIV-1 subtypes were determined for 300 of 306 women who received NVP in the HIV Network for Prevention Trials 012 study (158 women with subtype A and 105 women with subtype D). Infant infection status was known for 297 women. The cumulative rate of MTCT at 18 months was 13.2% for subtype A and 18.3% for subtype D (P=0.34). The rate of late transmission was 3.8% for subtype A and 7.6% for subtype D (P=0.28). Maternal baseline viral load was a significant predictor of MTCT, but maternal baseline CD4 cell count and subtype were not. CONCLUSIONS: No significant difference was observed in the rate of MTCT in women with subtype A versus D. There was a trend toward a higher rate of MTCT among women with subtype D, however, which was also apparent among women whose infants were infected after 8 weeks of age.

Health Systems and Maternal HIV/STIs
+ process evaluation
A paediatric and perinatal HIV/AIDS leadership initiative in Kingston, Jamaica
2004
Christie C D;
BACKGROUND AND PURPOSE: In Jamaica 1-2 of pregnant women are HIV-positive; 876 HIV-positive pregnant women will deliver and at least 283 newly infected HIV-infected infants will be born in 2003; HIV/AIDS is the leading cause of death in children aged one to four years. We describe a collaborative [quot]Town and Gown[quot] programme to address the paediatric and perinatal HIV epidemic in Kingston. METHOD: A team of academic and government healthcare personnel, comprising paediatricians, obstetricians, public health practitioners, nurses, microbiologists, data management and information technology personnel collaborated to address this public health emergency. RESULT: A five-point plan was implemented This comprised leadership and training of a core group of paediatric/perinatal HIVprofessionals to serve Greater Kingston and St Catherine and be a model for the rest of Jamaica. Mother-to-child transmission of HIV/AIDS is prevented by counselling and HIV-testing women in the antenatal clinics, giving azidothymidine (AZT) to HIV pregnant women beginning at 28 weeks gestation, throughout labour and to the HIV-exposed infants for the first six weeks of life. A unified parallel programme for identifying the HIV-infected infant and delivering paediatric HIV care at the major paediatric centres was implemented In three years, over 30,000 pregnant women are being tested for HIV; 600 HIV-exposed babies are being identified and about 140 paediatric

HIV infections will be prevented The team is building research capacity which emphasizes a strong outcomes-based research agenda and implementation of clinical trials. We are collaborating, locally, regionally and internationally. CONCLUSION: Collaboratively, the mission of reducing mother-to-child transmission of HIV/AIDS and improving the quality of life for those already living and affected by HIV/AIDS can be achieved

BP/Hypertension
+ Other evaluation design
Doppler ultrasound screening during the first trimester of pregnancy for preeclampsia: a cohort study: Bogotá, Colombia 2007 -2008 2009
Cortés-Yepes Hernán
Objetivos: determinar la utilidad diagnóstica y el poder de detección del índice de pulsatilidad anormal de las arterias uterinas durante el primer trimestre del embarazo en relación con la aparición de preeclampsia en una población de bajo riesgo. Metodología: estudio de cohorte prospectivo, en el cual se midió el índice de pulsatilidad de las arterias uterinas en 444 pacientes que asistieron a control prenatal normal entre las semanas 11 y 14 de gestación. Se evaluó de manera prospectiva la aparición de preeclampsia o hipertensión gestacional y preeclampsia severa y se determinaron las características operativas de esta prueba a diferentes puntos de corte. Resultados: en total, 30 pacientes presentaron preeclampsia o hipertensión gestacional (7,8%) y 6 desarrollaron preeclampsia severa (1,5%). El índice de pulsatilidad de las arterias uterinas durante el primer trimestre fue significativamente más alto en las mujeres que luego desarrollaron preeclampsia que en aquellas que no la presentaron (1,9 - 1,45, p=0,0001). Asimismo, este índice mostró un mejor desempeño para la detección de preeclampsia severa. Conclusión: el presente estudio demuestra que un Doppler anormal durante el primer trimestre se asocia de manera significativa con el desarrollo de preeclampsia. De este modo, esta prueba puede ser una herramienta útil para seleccionar a las mujeres que se beneficiarían de una vigilancia más estrecha durante el control prenatal.(AU) Objectives: this prospective study was aimed at determining the diagnostic usefulness and detection power of the abnormal pulsatility index in the uterine arteries during the first trimester of pregnancy related to the appearance of preeclampsia in a low-risk population. Methodology: this was a prospective cohort study of the uterine artery pulsatility rate in 444 patients who attended normal prenatal checkups between 11 to 14 weeks of pregnancy. It prospectively assessed the onset of preeclampsia or gestational hypertension and severe preeclampsia. This test's operative characteristics were determined at different cut-off points. Results: thirty patients suffered from gestational preeclampsia or gestational hypertension (7.8%) and six patients developed severe preeclampsia (1.5%). Uterine artery pulsatility rate during the first trimester was significantly higher in women who later developed preeclampsia than those who did not suffer (1.9 - 1.45, p=0.0001). Uterine artery pulsatility rate presented a better function for determining severe preeclampsia. Conclusions: the present study demonstrated that an abnormal Doppler result during the first trimester of pregnancy was significantly associated with developing preeclampsia. This test may be a useful tool for selecting women who could benefit from closer attention during prenatal checkups.(AU)

Health System
+ Policy review
Impact of organizational change on the delivery of reproductive services: a review of the literature.
2005
Ensor T ; Ronoh J ;
In order to understand the impact of specific maternal health interventions, it is necessary to understand the likely effect of the health system structure. An important aspect of this structure is the organizational culture. Many systems in low-income countries have been based on a centrally planned and financed system. In recent years a series of organizational changes have been introduced into many systems and these substantially alter the way in which the system operates and impacts on reproductive health care provision. The main changes reviewed in this paper are: (i) decentralization, (ii) privatization and (iii) integration and sector wide approaches. Each of these changes is seen to have important implications for reproductive health. In each case it is clear that the nature of the impact depends crucially on the way it is implemented. Quantifying the impact of these changes remains extremely difficult given the many different ways they can be introduced and the many confounding factors that affect the overall impact. The literature does, however, point to a number of key issues that impinge on the way in which change is likely to affect reproductive health initiatives. (author's)

Health System
+ Policy review
What drives health policy formulation: insights from the Nepal maternity incentive scheme?
2009
Ensor T ; Clapham S ; Prasai DP
Although maternal health outcomes have improved considerably in Nepal, continued low levels of skilled attendance and unequal access to safe emergency obstetric care continues to be central policy concern. The financial costs of delivery exacerbated are thought to continue to represent a major barrier to care to accessing services. Policy interest in this area moved swiftly. Skilled birth attendance came under the spotlight in 2001 while research on costs was commissioned in 2003. The resulting conclusions suggested substantial costs particularly on the demand side in the form of transport costs. After the research was completed the Government moved quickly to develop policy on financial barriers to skilled attendance leading to the Maternity Incentive Scheme that was implemented in 2005. We explored the reasons for policy acceptance and implementation based on recent studies in this area and a series of key informant interviews in the country. A variety of reasons can be shown to be important in ensuring that the research was utilised quickly. The

conduct of the research process was importance, particularly by ensuring that results were communicated widely in a way that responded to both technical and political policy-making concerns. A convergence of political interests that meant that the policy became an ideal vehicle for improving the flagging fortunes of the government was also seen as crucial in expediting policy change although it also meant that the policy had to be adjusted to cater to political rather purely technical concerns. The experience also underlines the importance of political champions within or close to government in advocating a strong policy line through channels that researchers can rarely access.

References

1. Medical Research Council United Kingdom. Developing and evaluating complex interventions: new guidance2009: Available from: www.mrc.ac.uk/complexinterventionsguidance.
2. Manandhar DS, Osrin D, Shrestha BP, Mesko N, Morrison J, Tumbahangphe KM, et al. Effect of a participatory intervention with women's groups on birth outcomes in Nepal: cluster-randomised controlled trial. Lancet. 2004 Sep 11-17;364(9438):970-9.
3. Harris RP, Helfand M, Woolf SH, Lohr KN, Mulrow CD, Teutsch SM, et al. Current methods of the US Preventive Services Task Force: a review of the process. Am J Prev Med. 2001 Apr;20(3 Suppl):21-35.
4. Anderson LM, Petticrew M, Rehfuess E, Armstrong R, Ueffing E, Baker P, et al. Using logic models to capture complexity in systematic reviews. Research Synthesis Methods;2(1):33-42. http://onlinelibrary.wiley.com/doi/10.1002/jrsm.32/abstract. 2011.
5. Blaauw D, Penn-Kekana M. Maternal Health. In: Fonn S, Padarath A, editors. South African Health Review 2010. Durban: Health Systems Trust; 2010. URL: http://www.hst.org.za/publications/876.
6. The World Bank. Country and Lending Groups: Low- and middle-income economies2012: Available from: http://data.worldbank.org/about/country-classifications/country-and-lending-groups.
